# Bandgap‐Engineered Pt_x_Te_y_:Ag_2_Te Composite Quantum Dots Enable Programmable NIR‐II Imaging and Photothermal‐Immune Synergistic Therapy

**DOI:** 10.1002/advs.75777

**Published:** 2026-05-20

**Authors:** Jing Liu, Rui Xu, Quan Wu, Tian‐Xin Xiao, Lai‐Xi Zhao, Lei Sun, Ya‐Wen Zheng, Liang Dong, Zhi‐Quan Tian, Haibo Wang

**Affiliations:** ^1^ Zhejiang Cancer Hospital Hangzhou Institute of Medicine Chinese Academy of Sciences Hangzhou China; ^2^ College of Chemistry and Molecular Sciences Wuhan University Wuhan China; ^3^ College & Hospital of Stomatology Anhui Medical University Anhui Province Key Laboratory of Oral Diseases Research Hefei China

**Keywords:** cancer therapy, materials science, molecular imaging, nanotechnology, photothermal therapy, quantum dot

## Abstract

Real‐time monitoring of tumor spatiotemporal dynamics and precise modulation of the tumor immune microenvironment are pivotal for effective cancer treatment. However, current platforms lack the ability to integrate quantitative molecular imaging with spatially confined immune modulation. Here, we report a nanotheranostic platform Pt_2_Te_3_:Ag_2_Te‐αPD‐L1 (PAPD) based on bandgap‐engineered Pt_x_Te_y_:Ag_2_Te quantum dots. Platinum‐induced electronic reconfiguration enables programmable bandgap modulation, yielding tunable near‐infrared IIb emission spanning from 1550 to 2200 nm. Pt_2_Te_3_:Ag_2_Te quantum dots exhibit stable emission at 1730 nm and controllable mild photothermal effects. Conjugation with αPD‐L1 antibodies endows the PAPD platform with dual tumor‐targeting capability through EPR‐mediated passive accumulation and PD‐L1‐specific active recognition. Crucially, near‐infrared IIb molecular imaging enables quantitative monitoring of tumor targeting and therapeutic response, meanwhile mild photothermal modulation induces immune reprogramming that sensitizes tumors to immune checkpoint immunotherapy in vivo. This work establishes a generalizable quantum‐engineered nanotheranostic platform, offering a scalable strategy for precision cancer treatment.

## Graphical abstract

A bandgap‐engineered Pt_x_Te_y_:Ag_2_Te quantum dot platform enables tunable NIR‐IIb emission for deep‐tissue imaging and mild photothermal therapy. Conjugation with αPD‐L1 confers dual tumor targeting and real‐time monitoring of PD‐L1 dynamics. Mild photothermal modulation induces immune reprogramming, enhancing checkpoint blockade efficacy and achieving synergistic antitumor therapy.

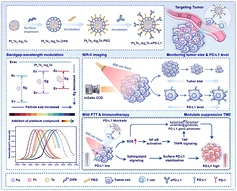

## 1 Introduction

The dynamic evolution of the tumor immune microenvironment (TIME) critically determines responsiveness to immunotherapy. Programmed death‐ligand 1 (PD‐L1), a central immune checkpoint molecule, suppresses cytotoxic T‐cell activation and facilitates tumor immune evasion through ligand‐receptor interactions with programmed cell death protein 1 (PD‐1) on T cells [1, 2]. Its expression exhibits marked spatial heterogeneity across tumor niches and temporal fluctuations driven by oncogenic signaling, inflammatory stimuli, metabolic stress, and therapeutic interventions. These dynamic changes govern the extent of T‐cell engagement and define the therapeutic window during which anti‐PD‐1/PD‐L1 therapies exert maximal efficacy [3, 4]. Consequently, real‐time, quantitative monitoring of PD‐L1 and associated immune signals is indispensable for guiding treatment decisions, capturing emerging resistance mechanisms, and identifying optimal intervention windows [5]. However, static or single time point measurements provide only a limited view of this evolving landscape and cannot reliably inform how the TIME will respond over the course of therapy [6].

Moreover, effective immunotherapy often requires deliberate and controlled modulation of the TIME to create permissive conditions for checkpoint inhibitors to function optimally [7]. Mild photothermal therapy (mild PTT, 39°C–45°C) has emerged as a non‐destructive approach to immunologically reprogram the TIME [8, 9, 10]. Controlled hyperthermia preserves tissue integrity while eliciting heat‐shock protein induction, pro‐inflammatory signaling, and enhanced antigen cross‐presentation and other key events that promote T‐cell priming and infiltration [11, 12]. Recent progress in photothermal immunotherapy has demonstrated that the combination of photothermal therapy and immune checkpoint blockade can effectively enhance immune responses against tumors [13, 14, 15]. Yet, current technologies largely lack the capacity to integrate real‐time molecular imaging with spatially and temporally precise immune modulation. This critical gap impedes the design of adaptive immunotherapy regimens tailored to the changing immune context of solid tumors [16, 17].

With the rapid advancement of nanomaterials and nanotechnology, an increasing number of nanotheranostic platforms, including semiconductor quantum dots (QDs), two‐dimensional nanostructures, and metal organic frameworks, have been explored for tumor imaging and therapy [18, 19, 20, 21]. However, these platforms remain largely disconnected from the molecular events that govern immunotherapy response. Most nanoplatforms provide structural or vascular contrast rather than real‐time readouts of immune‐regulatory pathways such as PD‐L1, and their thermal outputs are often fixed rather than tunable within biologically relevant ranges [22, 23]. Recent progress in second near‐infrared (NIR‐II, 1000–1700 nm) fluorescence imaging has enabled deep‐tissue and high‐contrast visualization of tumors with reduced scattering and autofluorescence [24, 25]. However, most NIR‐II emitters rely on heavy metals (e.g., Pb, Cd) or exhibit fixed emission bands, limiting their biological applicability and translational potential [26]. There is an urgent need for a theranostic platform that integrates quantitative PD‐L1 monitoring with spatially controlled mild photothermal modulation, thereby coupling immune surveillance with immune reprogramming [27].

**FIGURE 1:**
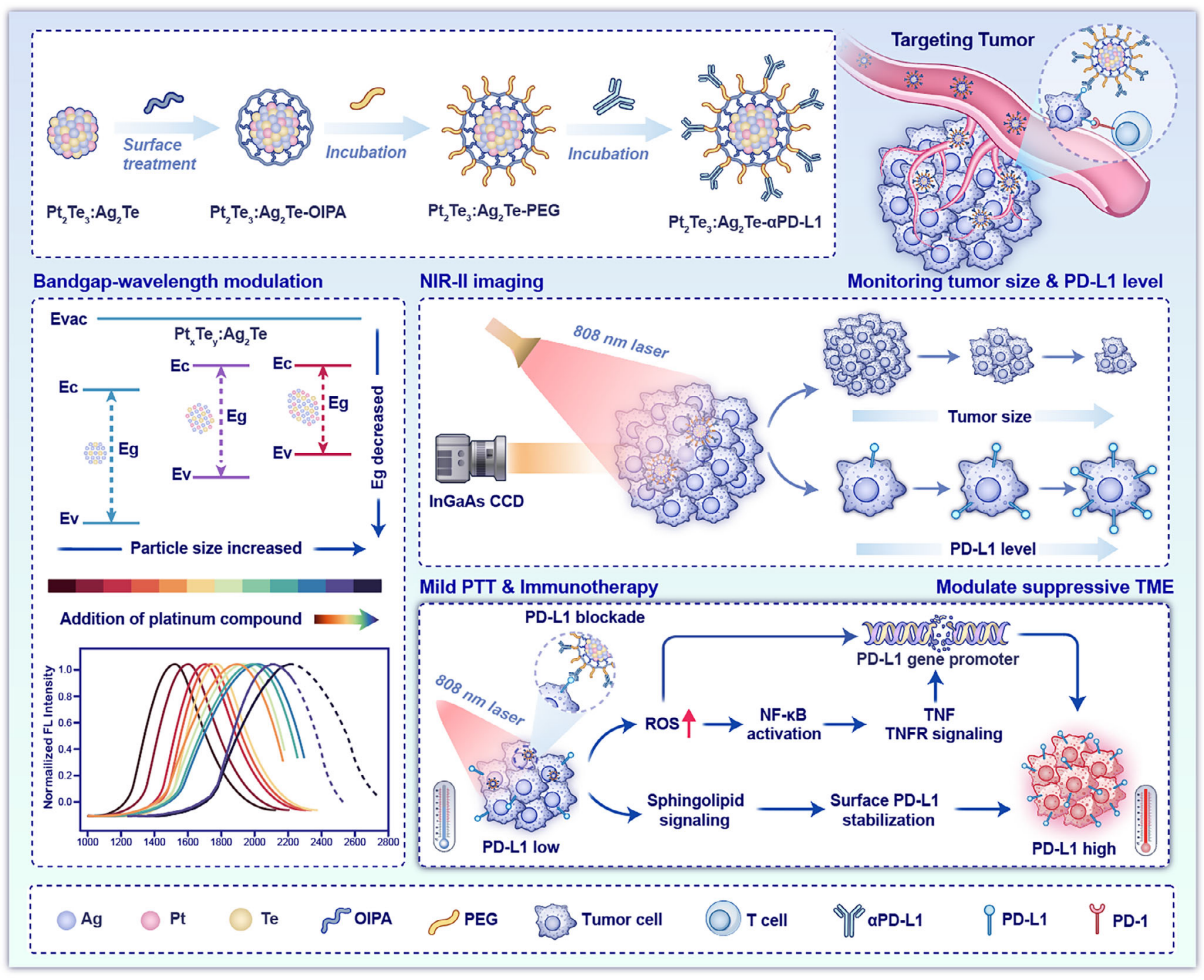
Bandgap‐engineered NIR‐IIb composite quantum dot PAPD platform for tumor imaging and immune‐modulated therapy.

Here, we developed an integrated nanotheranostic platform PAPD (Pt_2_Te_3_:Ag_2_Te‐αPD‐L1) based on bandgap‐engineered Pt_x_Te_y_:Ag_2_Te composite QDs (Figure 1). Platinum (Pt) incorporation introduces new electronic states and alters exciton recombination pathways, resulting in intrinsic bandgap modulation and programmable NIR‐IIb emission spanning 1550–2200 nm. The resulting Pt_2_Te_3_:Ag_2_Te QDs exhibit stable emission at 1730 nm and externally tunable mild photothermal heating within the sub‐ablative window, while maintaining excellent biocompatibility. Upon conjugation with αPD‐L1 antibodies, the PAPD construct achieves dual tumor targeting via enhanced permeability and retention (EPR)‐mediated accumulation and PD‐L1‐specific binding. Importantly, this platform enables simultaneous, real‐time visualization of PD‐L1 dynamics and targeted modulation of the TIME via mild photothermal stimulation. Such thermally induced immune rewiring not only enhances PD‐L1 expression but also promotes robust T‐cell and NK cell infiltration, thereby potentiating response to anti‐PD‐L1 therapy. Together, these findings provide a unified, adaptable framework for precision cancer immunotherapy where diagnosis, therapy, and immune modulation are intrinsically coupled through quantum engineering.

## 2 Results and Discussions

### 2.1 Composition‐Dependent Structural and Optical Properties of Pt_x_Te_y_:Ag_2_Te Composite Quantum Dots

**FIGURE 2:**
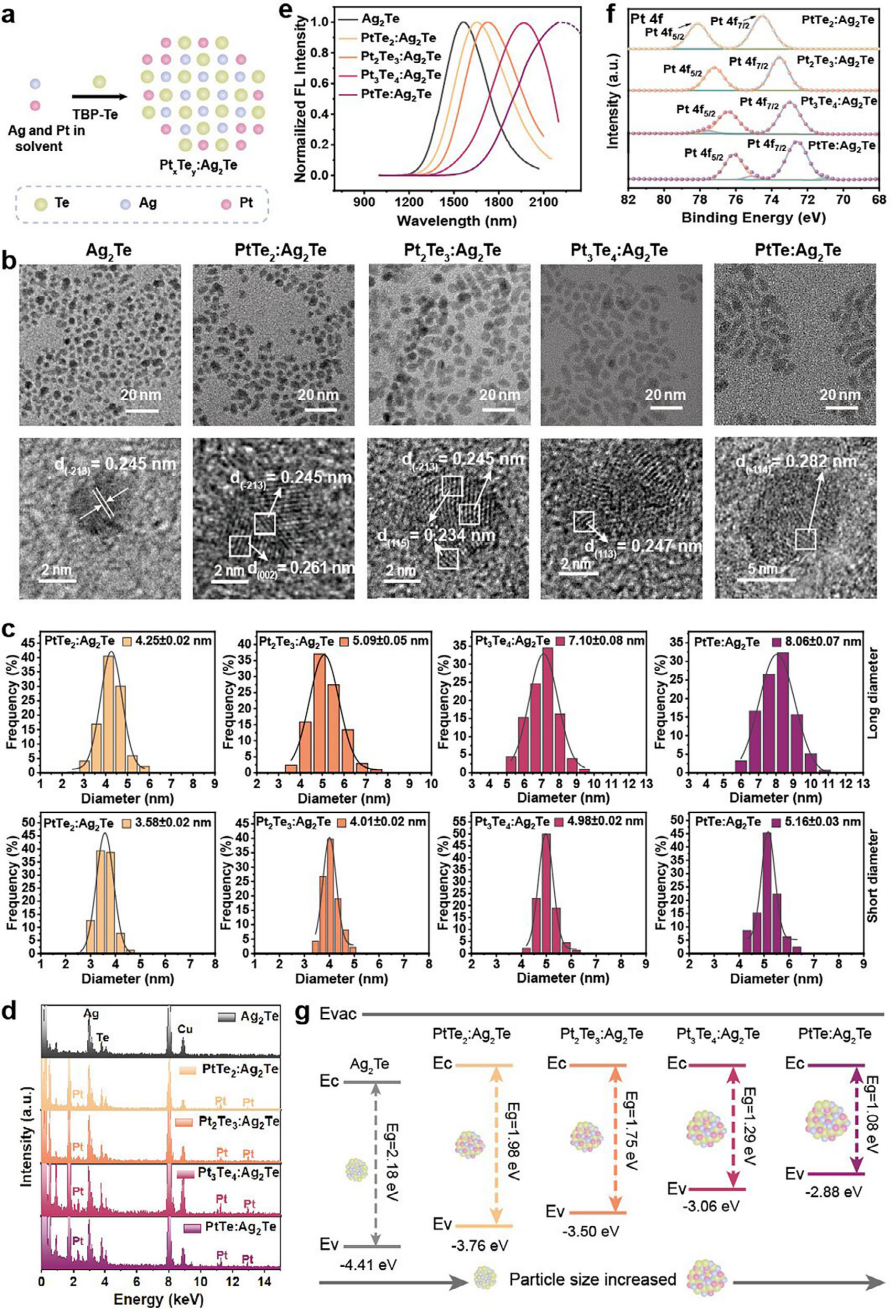
Synthesis and modulation mechanism of Pt_x_Te_y_:Ag_2_Te composite quantum dots. (a), Schematic illustration of the synthesis of Pt_x_Te_y_:Ag_2_Te composite QDs. Ag, Pt, and TBP‐Te precursors were combined in solution to yield ternary nanocrystals with tunable stoichiometry and composition. (b), TEM (top) and HRTEM (bottom) images of Ag_2_Te, PtTe_2_:Ag_2_Te, Pt_2_Te_3_:Ag_2_Te, Pt_3_Te_4_:Ag_2_Te, and PtTe:Ag_2_Te. Lattice fringes of various interplanar spacings are indicated, revealing crystalline domains and structural differences induced by varying Pt and Te doping levels. Scale bars: top row = 20 nm; bottom row = 2–5 nm. (c), Particle size distribution histograms for long and short diameters of each QD type (n ≥ 300 particles), fitted with a Gaussian distribution. Size tunability is observed across the different compositions. (d), EDS spectra acquired alongside TEM showed the elemental composition (Ag, Te, and Pt) of Ag_2_Te, PtTe_2_:Ag_2_Te, Pt_2_Te_3_:Ag_2_Te, Pt_3_Te_4_:Ag_2_Te, and PtTe:Ag_2_Te. The corresponding table summarizes the atomic percentage and estimated Pt content (mol%) for each formulation. (e), Normalized NIR‐II PL emission spectra of Ag_2_Te, PtTe_2_:Ag_2_Te, Pt_2_Te_3_:Ag_2_Te, Pt_3_Te_4_:Ag_2_Te, and PtTe:Ag_2_Te, demonstrating composition‐dependent emission tuning from 1550 to 2200 nm. (f), High‐resolution XPS spectra of the Pt 4f region for PtTe_2_:Ag_2_Te, Pt_2_Te_3_:Ag_2_Te, Pt_3_Te_4_:Ag_2_Te, and PtTe:Ag_2_Te, confirming the oxidation state of Pt in different samples. (g), Schematic band structure evolution of Ag_2_Te and Pt_x_Te_y_:Ag_2_Te QDs, showing progressive changes in E_v_ and E_g_ with increasing Pt content. The E_v_ and E_g_ values were derived from UV–vis–NIR DRS and UPS data.

Pt_x_Te_y_:Ag_2_Te composite QDs were synthesized by reacting Ag, Te, and varying amounts of Pt precursors (Figure 2a, Table S1). Morphological and structural analyses based on transmission electron microscopy (TEM) and high‐resolution TEM (HRTEM) revealed that Pt incorporation altered both lattice architecture and particle geometry. In detail, Ag_2_Te QDs exhibited uniform spherical morphology with an average diameter of 3.31 ± 0.01 nm (Figure 2b; Figure S1). HRTEM displayed well‐defined lattice fringes of 0.245 nm, corresponding to the (−213) plane of Ag_2_Te, while energy‐dispersive X‐ray spectroscopy (EDS) confirmed the presence of Ag and Te (Figure 2d; Table S2) [28].

Upon introduction of Pt, the nanocrystals transitioned from Ag_2_Te spheres to Ag_2_Te‐centered composite structures with distinct Pt─Te crystalline domains emerging at the particle periphery. At low Pt content, the resulting PtTe_2_:Ag_2_Te QDs exhibited two lattice regions in HRTEM: an inner 0.245 nm fringe associated with Ag_2_Te and an outer 0.261 nm fringe characteristic of the (002) plane of PtTe_2_ (Figure 2b) [29], with EDS validating Pt incorporation (Pt:Ag = 4.9%). Increasing the Pt precursor ratio yielded Pt_2_Te_3_:Ag_2_Te nanocrystals adopting elongated rice‐grain morphologies (average length 5.09 ± 0.05 nm, width 4.00 ± 0.09 nm, Figure 2c).

HRTEM revealed Ag_2_Te‐associated lattice spacing near the central region alongside newly formed 0.234 nm fringes at the edge, assigned to the (115) plane of Pt_2_Te_3_, indicating directional outward growth of Pt_2_Te_3_ domains. Further Pt incorporation resulted in Pt_3_Te_4_:Ag_2_Te with more pronounced anisotropic structures (Figure 2b), where peripheral lattice fringes of 0.247 nm ((113) facet of Pt_3_Te_4_) were contrasted with blurred central regions, consistent with substantial Pt─Te overgrowth partially obscuring the Ag_2_Te lattice [30, 31]. At a higher Pt incorporation, PtTe:Ag_2_Te QDs were obtained, characterized by dominant 0.282 nm lattice fringes corresponding to the (−202) facet of PtTe at the periphery [29]. While the interior region exhibited attenuated lattice contrast, likely resulting from extensive Pt─Te overgrowth on the Ag_2_Te during prolonged reaction.

X‐ray diffraction (XRD) analysis confirmed the presence of Ag_2_Te and Pt‐Te phases across all compositions, corroborating the HRTEM‐based structural identification, with peak broadening attributable to the nanoscale effects (Figure S2). Quantitative size analysis via TEM showed a monotonic increase in both long and short axes with rising Pt incorporation, consistent with the epitaxial growth of Pt─Te subdomains on Ag_2_Te (Figure 2c). X‐ray photoelectron spectroscopy (XPS) further corroborated the compositional evolution (Figure 2f; Figure S3). PtTe_2_:Ag_2_Te exhibited Pt^4^^+^‐dominant features (74.5/78.1 eV), whereas Pt_2_Te_3_:Ag_2_Te showed intermediate binding energies (73.7/77.1 eV), indicative of mixed Pt^2^^+^/Pt^4^^+^ states. With further Pt incorporation, Pt_3_Te_4_:Ag_2_Te displayed additional shifts toward lower binding energies (73.0/76.4 eV), and PtTe:Ag_2_Te featured predominantly Pt^2^^+^ species (72.6/76.0 eV), confirming a stepwise reduction of Pt species and sequential formation of PtTe_2_ → Pt_2_Te_3_ → Pt_3_Te_4_ → PtTe domains [32].

Optical characterization demonstrated a pronounced composition‐dependent modulation of photoluminescence (PL). Ag_2_Te QDs exhibited an emission peak at 1550 nm, whereas progressive Pt incorporation induced a systematic redshift, enabling continuous tuning of the PL wavelength across the 1550–2200 nm range (Figure 2e). A strong correlation between Pt ratio and emission wavelength was observed with constant Ag and Te inputs (Figure S4), highlighting the dominant contribution of Pt‐induced excitonic state reconstruction. Ultraviolet–visible–near–infrared (UV–vis–NIR) spectra revealed additional absorption bands in the 800–1000 nm region for Pt‐containing samples (Figure S5), attributable to Pt─Te‐related electronic transitions. To further elucidate the altered electronic structure, ultraviolet photoelectron spectroscopy (UPS) measurements were performed in conjunction with UV–vis–NIR diffuse reflectance spectroscopy (DRS)‐derived bandgap analysis (Figure S6). With increasing Pt incorporation, the valence band maximum (E_v_) progressively shifted upward, accompanied by a concomitant narrowing of the bandgap (E_g_) from 2.18 to 1.08 eV, as summarized in Figure 2g [33, 34].

These observations are consistent with partial electron transfer from Ag─Te orbitals into Pt‐d states, leading to localized charge redistribution and hybridization between Ag_2_Te and Pt_x_Te_y_ electronic domains. Collectively, these results demonstrate that systematic Pt incorporation drives hierarchical structural evolution and continuous electronic reconfiguration in Pt_x_Te_y_:Ag_2_Te composite QDs [35, 36]. This composition‐structure‐property coupling enables programmable bandgap modulation and underpins the broad tunability of NIR‐II emission from 1550 to 2200 nm.

### 2.2 Optical, Structural, and Functional Characterization of Pt_2_Te_3_:Ag_2_Te Quantum Dots for NIR‐IIb Imaging and Photothermal Therapy

Among the synthesized Pt_x_Te_y_:Ag_2_Te composite QDs, PL emission spans the NIR‐II window from 1550 to 2200 nm. Considering the signal‐to‐noise ratio and tissue scattering, Pt_2_Te_3_:Ag_2_Te QDs with a peak emission at 1730 nm were selected for subsequent NIR‐IIb imaging and photothermal investigations, as this wavelength lies within the optimal NIR‐IIb subwindow that minimizes tissue scattering and autofluorescence, while maintaining relatively strong emission intensity [20, 37]. As shown in Figures S7 and S8, QDs emitting at longer wavelengths (1970 and 2200 nm) fall outside the effective detection range of the current NIR‐II imaging system, while shorter‐wavelength QDs (1550 and 1650 nm) exhibit substantial background signals in biological tissues, resulting in unsatisfactory contrast and resolution. In contrast, Pt_2_Te_3_:Ag_2_Te (1730 nm) achieved a better balance between signal intensity, imaging contrast, and tissue penetration. In addition, photothermal measurements (Figure S9) further confirm its superior heating performance among the tested compositions.

**FIGURE 3:**
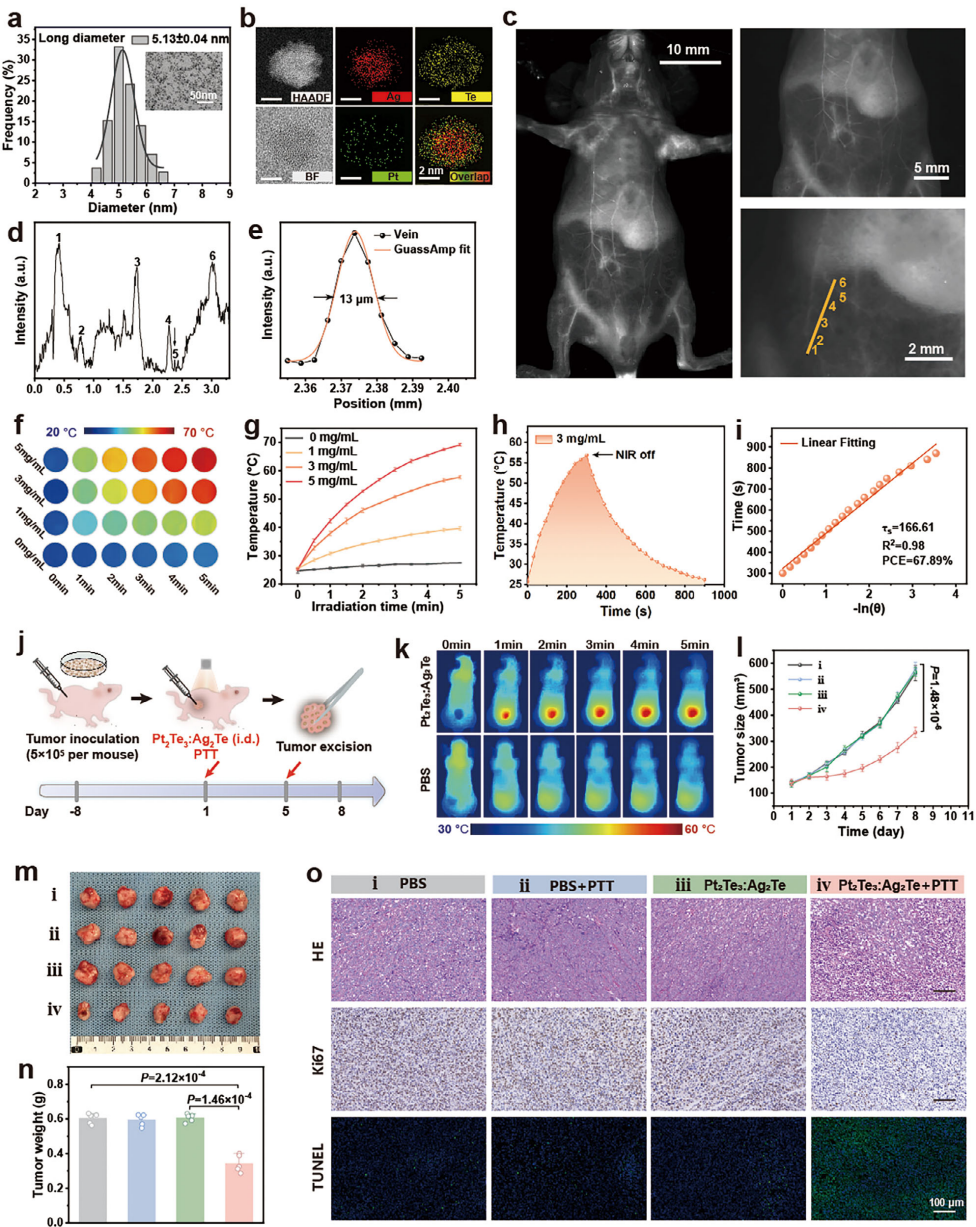
Structural, optical, and theranostic evaluation of Pt_2_Te_3_:Ag_2_Te composite QDs. (a), Size distribution histogram and TEM image inset of synthesized Pt_2_Te_3_:Ag_2_Te QDs, with an average long diameter of 5.13 ± 0.04 nm (n ≥ 300 particles). (b), HAADF‐STEM and corresponding EDS elemental mapping confirming the distribution of Ag (red), Te (yellow), and Pt (green) in the Pt_2_Te_3_:Ag_2_Te composite QD. (c), NIR‐II in vivo fluorescence image of vessels in the BALB/c nude mouse (6 weeks) and magnified fluorescence image of abdominal vessels. Fluorescence imaging was performed using a NIR‐II imaging system under a laser excitation power density of 80 mW/cm^2^ with a 1300 nm LP filter (200 ms exposure time). Scale bars: 2–10 mm. (d), Fluorescence cross‐sectional intensity profile measured along the orange line in panel c. (e), Gaussian fitting analysis of the smallest vessel (indicated by the arrow) with a spatial resolution of ∼13 µm. (f), Photothermal infrared thermal images of Pt_2_Te_3_:Ag_2_Te‐OIPA at different concentrations (0–5 mg/mL) under 808 nm laser irradiation (0.28 W/cm^2^) for 5 min, demonstrating concentration‐dependent photothermal conversion. (g), Photothermal heating curves of Pt_2_Te_3_:Ag_2_Te‐OIPA dispersions at varying concentrations (0, 1, 3, and 5 mg/mL) under 808 nm laser irradiation (0.28 W/cm^2^, 5 min). Data are presented as mean ± s.d. from three independent samples (*n* = 3). (h), Temperature changes of Pt_2_Te_3_:Ag_2_Te‐OIPA aqueous dispersion (3 mg/mL) during one laser on/off cycle under 808 nm laser irradiation (0.28 W/cm^2^). (i), Linear fitting of the cooling time data for Pt_2_Te_3_:Ag_2_Te‐OIPA (3 mg/mL) vs. −lnθ, used to calculate PCE. (j), Schematic illustration of the photothermal therapy procedure in tumor‐bearing nude mice. Mice were subcutaneously inoculated with MOC‐1 tumor cells and treated with intratumoral injection of Pt_2_Te_3_:Ag_2_Te followed by PTT: subcutaneous injection of 1 × 10^6^ tumor cells on Day −8, followed by intratumoral injection (i.d.) of Pt_2_Te_3_:Ag_2_Te on Day 1 and Day 5 with 808 nm laser irradiation, and tumor resection on Day 8. (k), Infrared photothermal images of MOC‐1 tumors in mice after injection with 30µL Pt_2_Te_3_:Ag_2_Te‐OIPA (3 mg/mL) or PBS under 808 nm laser irradiation (0.28 W/cm^2^, 5 min). (l), Tumor growth curves of mice in different treatment groups (i: PBS, ii: PBS+PTT, iii: Pt_2_Te_3_:Ag_2_Te, and iv: Pt_2_Te_3_:Ag_2_Te+PTT) over 8 days. Data are presented as mean ± s.d. (*n* = 5 per group). Two‐way ANOVA revealed a significant effect of Group (F (3, 128) = 474.7, P = 3.82 × 10^−^^6^ ^9^), Time (F (7, 128) = 1501.4, P = 1.42 × 10^−^^1^ ^1^ ^9^), and Group × Time interaction (F (21, 128) = 40.8, P = 1.36 × 10^−^^4^ ^6^). Post hoc Welch's *t*‐test between PBS and Pt_2_Te_3_:Ag_2_Te+PTT at day 8 showed a significant difference (P = 1.48 × 10^−^^6^). (m), Images of excised tumors harvested at the end of treatment (Day 8) from four groups (*n* = 5 per group). (n), Tumor weights of mice on day 8 after treatment. Data are presented as mean ± s.d. (*n* = 5 per group). Statistical significance was determined using one‐way ANOVA (F (3, 16) = 65.53, P = 3.32 × 10^−^^9^). Post hoc analysis was performed using Tukey's multiple comparison test (PBS vs. Pt_2_Te_3_:Ag_2_Te+PTT, P = 2.12 × 10^−4^; Pt_2_Te_3_:Ag_2_Te vs. Pt_2_Te_3_:Ag_2_Te+PTT, P = 1.46 × 10^−4^). (o), Histological and immunohistochemical analysis of tumor sections from the four groups: H&E staining (top row), Ki‐67 immunohistochemistry (middle row) for cell proliferation, and TUNEL staining (bottom row) for apoptosis. Scale bars: 100 µm.

As shown in Figure 3a, Pt_2_Te_3_:Ag_2_Te QDs exhibit a grain‐like morphology with an average long diameter of 5.13 ± 0.04 nm, while the corresponding short‐axis size distribution (4.00 ± 0.09 nm) was shown in Figure S10. High‐angle annular dark‐field scanning transmission electron microscopy (HAADF‐STEM) analysis revealed a distinct spatial distribution of constituent elements, with Pt enriched at the particle periphery, Ag predominantly localized in the central region, and Te uniformly distributed throughout the QD (Figure 3b). This elemental arrangement supports continued Pt_2_Te_3_ growth on the Ag_2_Te surface and is consistent with the outward formation of Pt‐rich domains. The photophysical properties of Pt_2_Te_3_:Ag_2_Te QDs were further evaluated. Time‐resolved fluorescence decay measurements revealed a marked prolongation of the PL lifetime from 4.82 ns for Ag_2_Te to 14.20 ns for Pt_2_Te_3_:Ag_2_Te (Figure S11a and Table S3), indicating modified exciton recombination dynamics induced by Pt incorporation. In addition, Pt_2_Te_3_:Ag_2_Te QDs exhibited an absolute photoluminescence quantum yield of 1.03% at an emission of 1730 nm (Figure 11b), demonstrating efficient emission within the NIR‐IIb region.

To ensure stable dispersion and colloidal stability in aqueous media, Pt_2_Te_3_:Ag_2_Te QDs were surface‐modified with oleylamine‐grafted polyacrylic acid (OIPA) [38]. In vitro fluorescence imaging in phosphate‐buffered saline (PBS) showed concentration‐dependent signal enhancement and a near‐linear relationship between fluorescence intensity and QD concentration (Figure S12a–b). Following tail‐vein injection of Pt_2_Te_3_:Ag_2_Te–OIPA (200 µL, 5 mg/mL), in vivo NIR‐II imaging clearly visualized the vascular network with minimal tissue autofluorescence and strong penetration depth (Figure 3c). Notably, a small blood vessel with a width of approximately 13 µm was resolved by Gaussian fitting of the cross‐sectional intensity profile (Figure 3d–e), indicative of the high spatial resolution achievable with the QDs.

To determine the representative irradiation condition, we systematically investigated the effect of laser power density (0.14, 0.28, and 0.42 W/cm^2^) under 808 nm on photothermal heating behavior (Figure S13). Based on these results, the temperature range achieved at 0.28 W/cm^2^ was considered suitable for controllable photothermal heating. Therefore, the photothermal performance of Pt_2_Te_3_:Ag_2_Te‐OIPA QDs at different concentrations was further evaluated under this condition. Thermal imaging revealed a concentration‐dependent temperature increase in the range of 0 to 5 mg/mL. The concentration of Pt_2_Te_3_:Ag_2_Te‐OIPA QDs was optimized at 3 mg/mL based on a comprehensive comparison of all experimental results. Under these conditions, a moderate and controllable temperature increase from 25.6°C to 57.7°C was achieved within 5 min (Figure 3f–g), which falls within the effective temperature range for high‐temperature photothermal ablation. The QDs further exhibited excellent photothermal stability over five heating‐cooling cycles (Figure S14). And it has a high photothermal conversion efficiency of 67.89% (Figure 3h–i), which is higher than that of comparable photothermal nanomaterials (Table S4).

The in vivo biosafety of Pt_2_Te_3_:Ag_2_Te‐OIPA was systematically evaluated prior to therapeutic studies [39]. The results showed no significant hematological or serum biochemical abnormalities by day 14 (Figures S15 and S16). Inductively coupled plasma (ICP) analysis showed a gradual decrease in blood Ag levels over time (Figure S17). Hematoxylin and eosin (H&E) staining of major organs revealed no pathological abnormalities (Figure S18), confirming favorable in vivo biocompatibility.

The photothermal therapeutic efficacy of Pt_2_Te_3_:Ag_2_Te‐OIPA (3 mg/mL) was further assessed in murine oral carcinoma 1 (MOC‐1) tumor‐bearing mice. Two rounds of laser irradiation were applied (808 nm, 0.28 W/cm^2^, 5 min) and tumors were excised on day 8 (Figure 3j). Tumors treated with Pt_2_Te_3_:Ag_2_Te‐OIPA exhibited a rapid temperature increase from 35.9°C to 56.5°C, whereas tumors in the PBS control group showed only a slight increase from 35.9°C to 38.3°C (Figure 3k; Figure S19). This localized hyperthermia resulted in pronounced tumor growth suppression, as evidenced by reduced tumor volumes and final tumor weights (Figure 3l–n). Histological analyses confirmed the therapeutic efficacy. Hematoxylin and eosin (H&E) staining showed extensive necrosis in treated tumors, Ki‐67 antigen (Ki‐67) staining was markedly reduced, and terminal deoxynucleotidyl transferase dUTP nick end labeling (TUNEL) analysis revealed widespread apoptosis (Figure 3o) [40].

Collectively, these results establish Pt_2_Te_3_:Ag_2_Te as a biocompatible, photothermally responsive, and NIR‐IIb emissive nanoplatform with tunable optical output and reproducible photothermal activity. Its long‐wavelength emission enables high‐contrast deep‐tissue imaging with micrometer‐scale vascular resolution, while its robust and reproducible photothermal performance supports effective tumor ablation under controlled irradiation, providing a solid foundation for subsequent tumor‐responsive nanotheranostic designs.

### 2.3 Photothermally Induced PD‐L1 Upregulation and Immune Reprogramming

Given the high photothermal conversion efficiency and precise temperature controllability of Pt_2_Te_3_:Ag_2_Te‐OIPA QDs, we investigated whether nanoparticle‐mediated mild photothermal stimulation could be achieved in a controllable manner.

**FIGURE 4:**
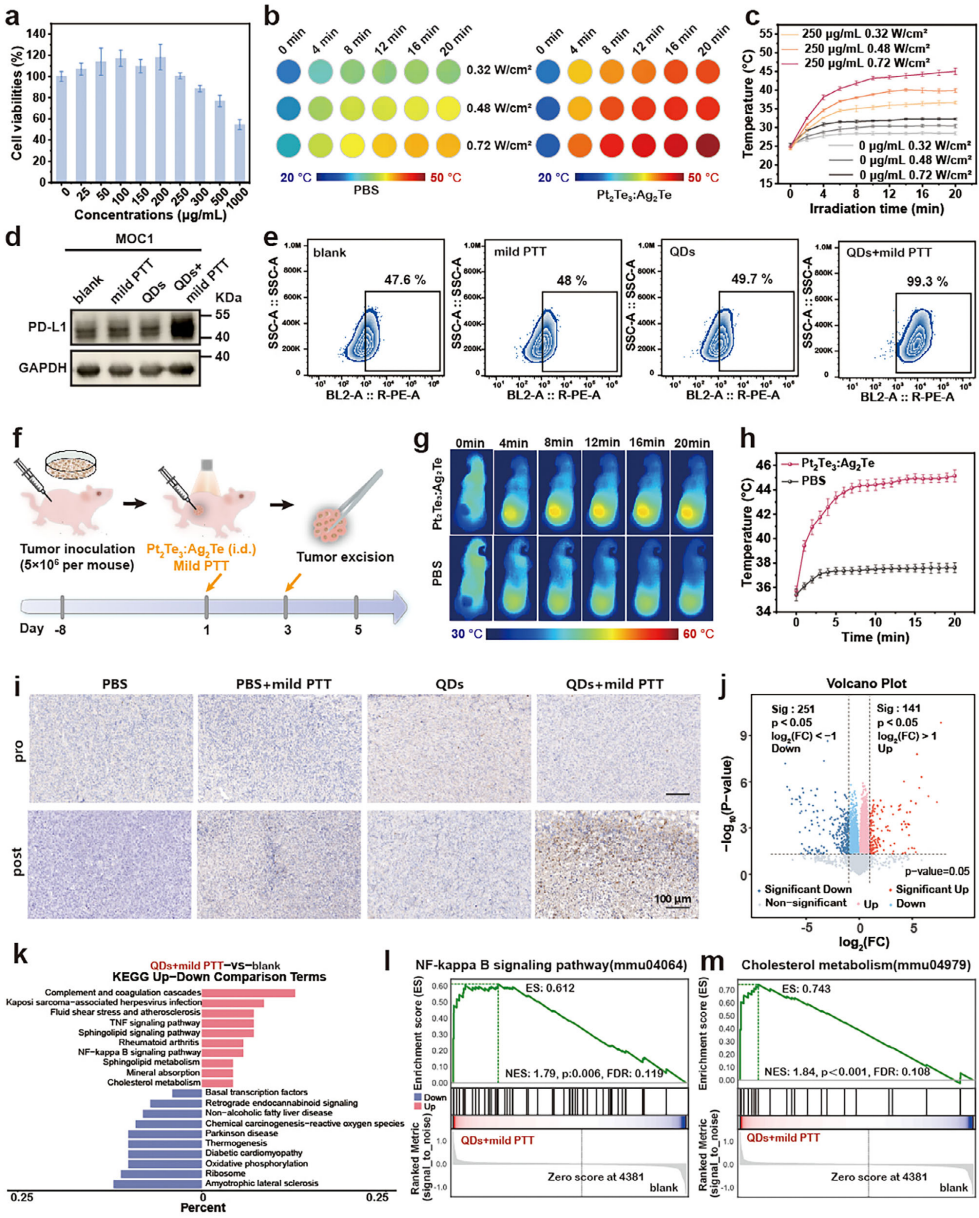
Pt_2_Te_3_:Ag_2_Te QDs‐mediated mild photothermal treatment enhances PD‐L1 expression and modulates the tumor immune microenvironment. (a), Cell viability of human umbilical vein endothelial cells (HUVECs) treated with varying concentrations (0–1000 µg/mL) of Pt_2_Te_3_:Ag_2_Te QDs after 24 h, assessed by CCK‐8 assay. Results are shown as mean ± s.d. (*n* = 3). (b), Infrared thermal images of PBS and Pt_2_Te_3_:Ag_2_Te QDs (250 µg/mL) exposed to 808 nm laser irradiation (0.32, 0.48, and 0.72 W/cm^2^) at different time points (0–20 min). (c), Quantitative temperature elevation curves of PBS and Pt_2_Te_3_:Ag_2_Te QDs (250 µg/mL) under varying power densities (0.32, 0.48, and 0.72 W/cm^2^) of 808 nm laser irradiation for 20 min. Data are shown as mean ± s.d. (n = 3). (d), Western blot analysis of PD‐L1 protein expression in MOC‐1 cells following different treatments: untreated control (Blank), laser irradiation alone (mild PTT), Pt_2_Te_3_:Ag_2_Te QDs (QDs), and combined Pt_2_Te_3_:Ag_2_Te QDs + PTT (QDs+ mild PTT) treatment. GAPDH was used as a loading control. Protein lysates were harvested at 48 h after treatment initiation. Mild photothermal stimulation was applied twice, at 0 and 24 h, to assess its effect on PD‐L1 upregulation. (e), Flow cytometric analysis of PD‐L1 expression on MOC‐1 tumor cells from the same treatment groups as in (d), confirming increased PD‐L1 surface levels in the QDs + mild PTT group. Cells were collected at 48 h after initial treatment, with mild photothermal stimulation administered at 0 and 24 h. (f), Schematic representation of the in vivo therapeutic protocol. BALB/c nude mice were subcutaneously inoculated with MOC‐1 cells (1 × 10^6^ cells per mouse). On day 1 and day 3, mice received intratumoral injection of Pt_2_Te_3_:Ag_2_Te QDs followed by mild PTT. Tumors were excised on day 5 for subsequent immunological evaluation. (g), Representative infrared thermal images showing tumor temperature changes in BALB/c nude mice during 20 min of mild PTT treatment (808 nm laser, 0.64 W/cm^2^) after intratumoral injection with 60 µL of Pt_2_Te_3_:Ag_2_Te QDs (250 µg/mL) or PBS. (h), Temperature elevation curves of tumor sites in BALB/c nude mice during mild PTT treatment (808 nm laser, 0.64 W/cm^2^, 20 min), following intratumoral injection with 60 µL of Pt_2_Te_3_:Ag_2_Te QDs (250 µg/mL) or PBS. Tumor surface temperature was recorded using an infrared thermal camera at multiple time points. Data are presented as mean ± s.d. (*n* = 3). (i), Representative immunohistochemical (IHC) staining of PD‐L1 expression in tumor sections before and after different treatments. Scale bar: 100 µm. (j), Proteomic profiling reveals differential protein expression. Volcano plot illustrating differentially expressed proteins (DEPs) between the QDs + mild PTT group and untreated control tumors. Significantly upregulated proteins (log_2_FC > 1, *p* < 0.05) are shown in red, and downregulated proteins (log_2_FC < −1, *p* < 0.05) in blue. (k), Enrichment of immune and metabolic pathways after treatment. (l, m), Protein set enrichment supports immune activation and lipid remodeling. GSEA plots demonstrating positive enrichment of NF‐κB signaling (l) and cholesterol metabolism (m) pathways in tumors from the QDs + mild PTT group.

Following confirmation of photothermal performance, the cytocompatibility of Pt_2_Te_3_:Ag_2_Te‐OIPA QDs was first assessed in the absence of laser irradiation. CCK‐8 assays showed that cell viability remained above ∼90% over a wide concentration range (25–300 µg/mL), with no significant cytotoxicity observed at the working concentrations used in subsequent experiments (Figure 4a). The tunability of photothermal output was evaluated by varying both nanoparticle concentration and laser power density. Temperature elevation profiles recorded under identical laser irradiation (0.72 W/cm^2^, 20 min) showed a clear concentration‐dependent heating behavior (Figure S20). At the concentration of 250 µg/mL, increasing laser power densities (0.32, 0.48, and 0.72 W/cm^2^) resulted in stable temperature plateaus of approximately 41°C–43°C, 45°C–47°C, and ∼50°C respectively, while PBS controls remained near baseline (Figure 4b–c). Pt_2_Te_3_:Ag_2_Te‐OIPA QDs further exhibited excellent mild photothermal stability over five successive on/off irradiation cycles (Figure S21). Consistent heating behavior was also observed when the dispersions were tested in cell culture plates, confirming that mild hyperthermia could be reliably achieved under biologically relevant conditions (Figure S22). Collectively, these results establish that Pt_2_Te_3_:Ag_2_Te‐OIPA enables precise and stable photothermal modulation within the immune‐relevant mild hyperthermia window without inducing direct thermal ablation.

We then investigated whether such mild photothermal stimulation could modulate immune checkpoint expression. Western blotting revealed a marked elevation of PD‐L1 protein levels in MOC‐1 tumor cells following nanoparticle‐mediated mild photothermal treatment (Figure 4d; Figure S23) [41]. Flow cytometry analysis confirmed this finding, showing a pronounced rightward shift in PD‐L1 fluorescence intensity compared with untreated controls (Figure 4e; Figure S24). The regulatory effect was further validated in vivo. Tumor‐bearing mice received intratumoral injections of Pt_2_Te_3_:Ag_2_Te‐OIPA QDs followed by localized mild photothermal irradiation (Figure 4f). Thermal imaging and quantitative temperature mapping showed that only the Pt_2_Te_3_:Ag_2_Te group achieved stable intratumoral temperatures in the 42°C–45°C range, whereas the PBS group remained at ∼37°C–39°C (Figure 4g–h). Immunohistochemistry of tumor sections confirmed significant PD‐L1 upregulation in treated tumors, consistent with the in vitro results (Figure 4i).

To delineate the molecular mechanism underlying the photothermal‐immune response, unbiased quantitative proteomic profiling of MOC‐1 cells exposed to different treatments was performed. Volcano plot analysis identified 141 significantly upregulated and 251 downregulated proteins (*p* < 0.05; |log_2_FC| > 1) in Pt_2_Te_3_:Ag_2_Te‐OIPA QDs group compared with the untreated group (Figure 4j). Kyoto Encyclopedia of Genes and Genomes (KEGG) analysis showed upregulated enrichment of inflammatory and immune pathways (including the NF‐κB signaling pathway, TNF signaling pathway, sphingolipid metabolism, and complement‐coagulation cascades), whereas downregulated enrichment was observed in mitochondrial energy metabolism pathways, including oxidative phosphorylation and thermogenesis (Figure 4k), indicating an immuno‐metabolic rewiring under mild photothermal stress. Gene set enrichment analysis (GSEA) further highlighted a coordinated activation of NF‐κB signaling (Figure 4l; Figure S25) and cholesterol metabolism pathways (Figure 4m; Figure S26), indicating a concomitant inflammatory activation and lipid/metabolic rewiring after mild photothermal stimulation. Notably, the GO term “positive regulation of reactive oxygen species (ROS) metabolic process” also displayed a strong enrichment score (Figure S27), supporting redox remodeling as a prominent feature of the photothermal response that may act upstream to couple oxidative‐inflammatory signaling with PD‐L1 induction.

To experimentally assess this redox response, we further measured intracellular ROS levels in MOC‐1 cells using a fluorescent ROS probe followed by flow cytometry. Neither QDs alone nor mild photothermal treatment alone caused an obvious increase in intracellular ROS levels compared with the control group. In contrast, QDs combined with mild photothermal treatment induced a pronounced increase in ROS fluorescence intensity (Figure S28). These results indicate that Pt_2_Te_3_:Ag_2_Te‐OIPA QDs do not directly generate substantial ROS under basal conditions, whereas the combined QDs‐mediated mild photothermal treatment triggers intracellular ROS accumulation.

Mechanistically, several of the enriched pathways converge on known PD‐L1 regulatory circuits: NF‐κB activation enhances PD‐L1 promoter activity through p65/RelA binding [42]; TNF signaling synergizes with IFN‐γ to reinforce PD‐L1 transcription via JAK‐STAT/IRF1 cascades [43]; sphingolipid signaling modulates membrane microdomains to stabilize PD‐L1 surface retention [44]; and complement coagulation activation triggers cytokine release that sustains STAT3/NF‐κB cross‐talk [45]. Together with the observed ROS accumulation, these results delineate an ROS‐associated inflammatory stress‐response axis linking mild photothermal stress to PD‐L1 upregulation and immune reprogramming.

Among these pathways, NF‐κB represents a central and pharmacologically tractable node closely associated with PD‐L1 transcriptional regulation. To further test the contribution of NF‐κB signaling to mild photothermal treatment‐induced PD‐L1 upregulation, MOC‐1 cells were pretreated with an NF‐κB inhibitor before exposure to Pt_2_Te_3_:Ag_2_Te‐OIPA QDs combined with mild photothermal treatment, PD‐L1 protein expression was assessed by western blotting at 48 h after treatment. Consistent with the above results, QDs‐mediated mild photothermal treatment markedly increased PD‐L1 expression compared with untreated controls. Notably, NF‐κB inhibitor pretreatment substantially attenuated this PD‐L1 upregulation, whereas inhibitor treatment alone did not obviously alter basal PD‐L1 expression (Figure S29). These data provide pharmacological evidence that NF‐κB signaling contributes, at least in part, to PD‐L1 induction triggered by QDs‐mediated mild photothermal stress.

Collectively, these results showed that Pt_2_Te_3_:Ag_2_Te‐OIPA QDs enable finely tunable mild photothermal stimulation within the immune‐modulatory temperature range, leading to PD‐L1 upregulation in tumor cells both in vitro and in vivo. Proteomic analyses revealed the involvement of NF‐κB/TNF‐centered inflammatory signaling, metabolic rewiring, and ROS‐associated stress responses in this photothermal‐immune coupling. Further ROS detection and NF‐κB inhibition experiments confirmed intracellular redox remodeling and demonstrated that NF‐κB signaling contributes, at least in part, to mild photothermal treatment‐induced PD‐L1 upregulation. These findings establish a mechanistic basis for combining mild PTT modulation with PD‐L1 checkpoint blockade in subsequent therapeutic designs.

### 2.4 Construction and Tumor‐Targeting Evaluation of the PAPD Nanoplatform

To enable tumor‐targeted imaging and synergistic mild photothermal immunotherapy, we constructed a multifunctional theranostic nanoplatform (PAPD) by conjugating αPD‐L1 antibodies onto hydrophilized Pt_2_Te_3_:Ag_2_Te QDs, thereby integrating NIR‐II fluorescence, photothermal capability, and immune checkpoint targeting within a single platform.

**FIGURE 5:**
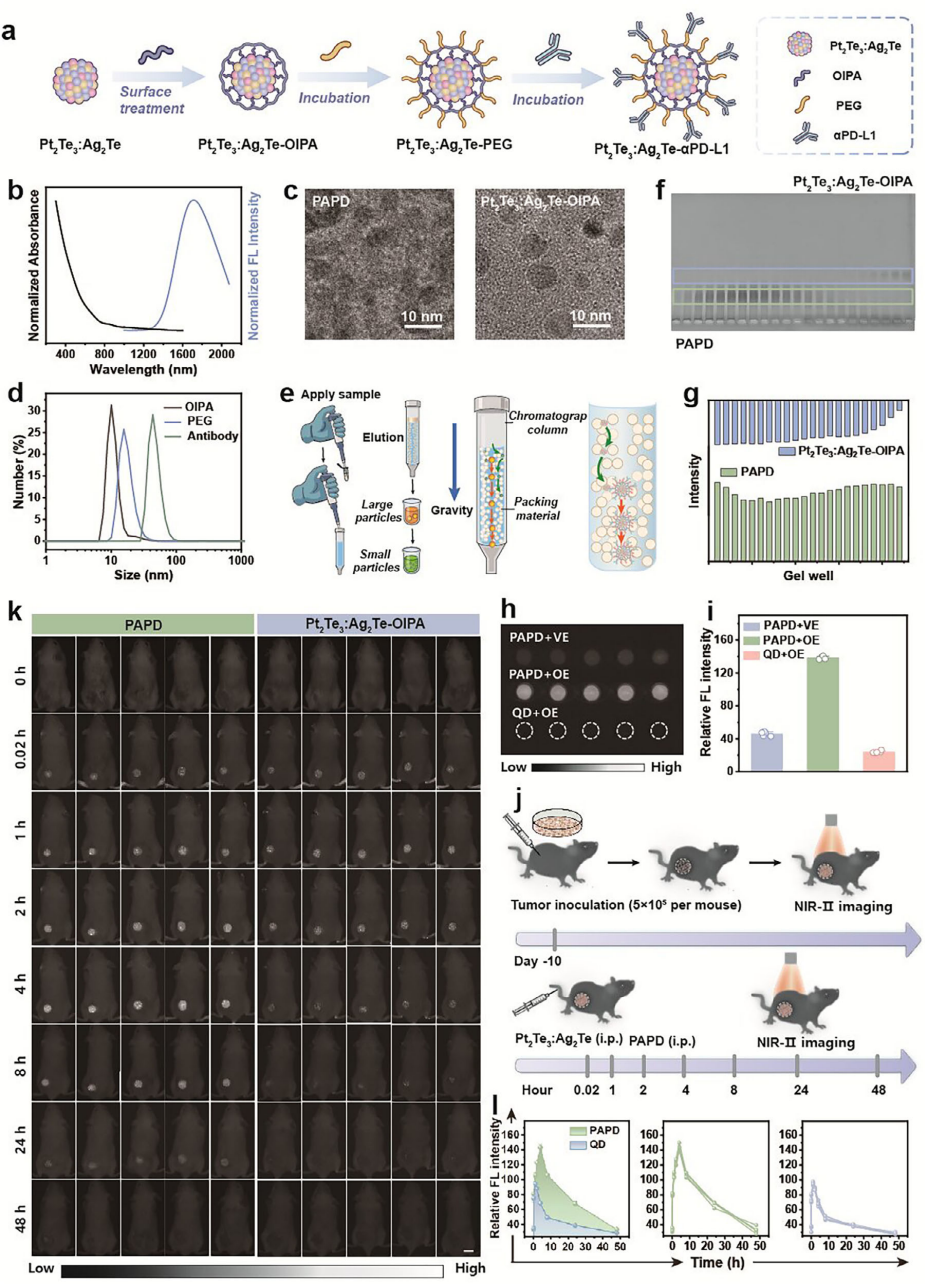
Tumor‐targeted NIR‐II fluorescence imaging enabled by PAPD. (a), Schematic illustration of the surface modification steps of Pt_2_Te_3_:Ag_2_Te QDs and their assembly into PAPD (Pt_2_Te_3_:Ag_2_Te‐αPD‐L1). (b), Normalized absorption and NIR‐II fluorescence emission spectra of PAPD in aqueous solution. The sample was excited with an 808 nm laser and detected using a 1300 nm long‐pass (LP) filter. (c), TEM images of Pt_2_Te_3_:Ag_2_Te‐OIPA QDs and PAPD nanoplatform showing morphological differences. Phosphotungstic acid was used as a negative stain. Scale bars, 10 nm. (d), DLS analysis showing hydrodynamic diameters of the nanoplatform after each modification step (OIPA, PEG, and antibody), all measurements were conducted in PBS at 25°C. (e), Schematic representation of gravity‐driven size exclusion chromatography using Superdex 200 prep‐grade resin for purification of the PAPD. (f), Purification of PAPD using size exclusion chromatography, followed by validation with agarose gel electrophoresis. The clear separation between Pt_2_Te_3_:Ag_2_Te‐OIPA (blue) and functionalized PAPD (green) confirmed high‐purity nanoplatform isolation. (g), Quantitative analysis of fluorescence intensity based on the agarose gel bands in panel (f). Fluorescence intensity was extracted by densitometric analysis of gel lanes using ImageJ. (h), In vitro NIR‐II fluorescence imaging of PD‐L1‐OE cells and PD‐L1‐VE cells after incubation with PAPD or control QDs. Cells (2 × 10^5^ per well) were incubated with 250 µg/mL PAPD in serum‐free medium for 4 h at 37°C. Imaging was performed under 808 nm excitation, using a 1300 nm LP filter and 500 ms exposure time. (i), Quantification of cellular NIR‐II fluorescence signals from panel (h). Data are presented as mean ± s.d. (*n* = 5). (j), Schematic of the in vivo imaging protocol. C57BL/6 mice were subcutaneously inoculated with MOC‐1 tumor cells (5 × 10^5^ cells per mouse) on Day −10. On day 0, mice received intravenous injection of 200 µL of PAPD or Pt_2_Te_3_:Ag_2_Te‐OIPA (5 mg/mL) via the tail vein. NIR‐II fluorescence imaging was conducted at multiple time points post‐injection (0.02, 1, 2, 4, 8, 24, and 48 h). (k), NIR‐II fluorescence images of tumor‐bearing C57BL/6 mice at different time points (0.02, 1, 2, 4, 8, 24, and 48 h) following intravenous injection of PAPD or Pt_2_Te_3_:Ag_2_Te‐OIPA QDs (200 µL, 5 mg/mL) via the tail vein. Imaging was performed under 808 nm laser excitation using a 1300 nm LP filter; fluorescence and bright‐field exposure times were 200 and 5 ms respectively. Scale bar: 1 cm. (l), Time‐resolved quantification of fluorescence intensity at the tumor site. Data represent mean ± s.d. (*n* = 5 per group).

As illustrated in Figure 5a, the fabrication of PAPD involves hydrophilic surface modification of Pt_2_Te_3_:Ag_2_Te QDs, activation of surface functional groups, and subsequent antibody conjugation, yielding a stable water‐dispersible nanoplatform. UV–vis–NIR absorption and PL spectra confirmed that NIR‐II fluorescence was well preserved after antibody conjugation (Figure 5b), indicating that the intrinsic band structure and optical performance remained uncompromised. TEM revealed distinct morphological changes upon surface modification. While hydrophilic Pt_2_Te_3_:Ag_2_Te QDs displayed uniform dispersion with well‐defined edges, PAPD exhibited a faint halo surrounding the particles and progressively blurred boundaries (Figure 5c), consistent with the presence of surface‐conjugated biomolecules. Dynamic light scattering (DLS) showed a stepwise increase in hydrodynamic diameter from 10.1 nm (QDs) to 43.8 nm (PAPD), placing it within the optimal EPR range (20−200 nm) (Figure 5d). In parallel, the zeta potential shifted from −38.9 to −6.02 mV (Figure S30a), reflecting gradual surface charge neutralization [46]. Moreover, agarose gel electrophoresis demonstrated reduced electrophoretic mobility of PAPD compared with unmodified QDs (Figure S30b), consistent with the zeta potential changes and supporting successful antibody conjugation.

PAPD was subsequently purified by size‐exclusion chromatography (SEC), as depicted in Figure 5e. Post separation agarose gel electrophoresis showed a clear migration shift of PAPD relative to bare QDs (Figure 5f), and fluorescence intensity quantification of gel bands confirmed enrichment of the target conjugate (Figure 5g). Fourier‐Transform Infrared (FTIR) spectroscopy further validated surface chemical modification. In addition to characteristic C═O (1600–1650 cm^−^^1^) and O─H (3400 cm^−^^1^) stretching vibrations observed in both PAPD and bare QDs, new absorption bands at ∼1050 and ∼1250 cm^−^^1^ were assigned to C─O stretching and amide III C─N stretching vibrations respectively, confirming successful antibody conjugation (Figure S31) [47, 48]. Moreover, the colloidal stability of PAPD was evaluated over 30 days, both the hydrodynamic diameter and zeta potential remained largely unchanged (Figures S32 and S33), ensuring long‐term functionality.

With the structural and physicochemical properties validated, the tumor‐targeting capability of PAPD was next evaluated. In vitro fluorescence imaging was performed using tumor cells with different PD‐L1 expression levels. The fluorescence intensity in PD‐L1‐overexpressing (OE) cells far exceeded that in vector‐expressing (VE) controls (Figure 5h–i), indicating that the tumor‐targeting capability of PAPD was mediated by the specific recognition. The in vivo tumor‐targeting performance of PAPD was further evaluated in murine tumor xenograft models. Following tail‐vein injection of PAPD or Pt_2_Te_3_:Ag_2_Te QDs (Figure 5j), NIR‐II fluorescence imaging revealed initial tumor accumulation in both groups within the first hour (Figure 5k). Notably, only the PAPD group exhibited sustained and enhanced tumor retention, with fluorescence intensity peaking at 4 h and remaining detectable for up to 24 h. Fluorescence from bare QDs became diffuse and declined rapidly and nearly disappearing by 8 h. Quantitative analysis confirmed significantly higher and prolonged tumor signal retention in the PAPD group compared to the Pt_2_Te_3_:Ag_2_Te‐OIPA group (Figure 5l), suggesting that active targeting significantly enhances probe accumulation at the tumor site beyond the EPR effect. To further assess targeting specificity, xenograft models derived from PD‐L1‐OE and PD‐L1‐VE oral squamous carcinoma cells were established. At 4 h post‐injection, the PD‐L1‐OE tumors exhibited significantly stronger signals than PD‐L1‐VE tumors (Figure S34), with an approximately 1.6‐fold increase in intensity. This result demonstrates the PD‐L1‐dependent targeting specificity of PAPD, consistent with the in vitro observations.

Collectively, these results confirm the successful construction of the PAPD nanoplatform with preserved NIR‐II emission and effective tumor targeting in vitro and in vivo, providing a foundation for subsequent imaging and therapeutic studies.

### 2.5 In Vivo Evaluation of PAPD Platform for Tumor Imaging, Mild Photothermal Therapy, and Immunotherapy Sensitization

**FIGURE 6:**
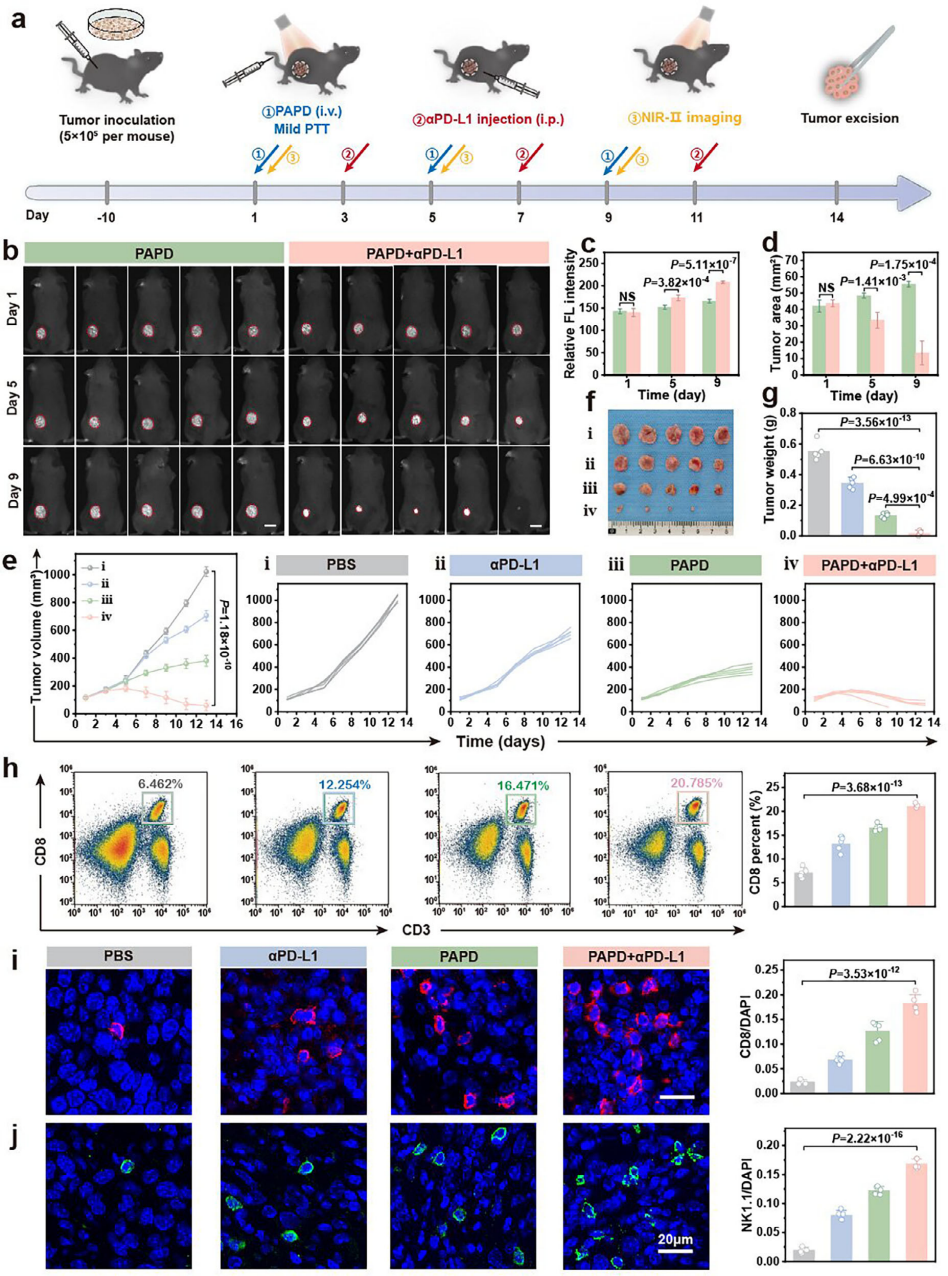
Evaluation of synergistic antitumor efficacy of PAPD‐mediated mild photothermal therapy combined with αPD‐L1 immunotherapy. (a), Schematic of the in vivo treatment and imaging schedule. Mice were randomly assigned to four treatment groups: (i) PBS control (no intervention); (ii) αPD‐L1 monotherapy (intraperitoneal injection of αPD‐L1 antibodies on days 3, 7 and 11); (iii) PAPD‐only group (intravenous injection of PAPD on days 1, 5 and 9, followed by NIR‐II imaging and mild PTT at 4 h post‐injection); and (iv) combination group (receiving both PAPD and αPD‐L1 treatment at the same respective time points). C57BL/6 mice were subcutaneously inoculated with 5 × 10^5^ MOC‐1 tumor cells on day −10. On days 1, 5, and 9, mice received a single intravenous injection of PAPD (200 µL, 5 mg/mL) followed by mild PTT using an 808 nm laser (0.64 W/cm^2^, 20 min) after 4 h of injection, and NIR‐II fluorescence imaging was also performed 4 h post‐injection. αPD‐L1 antibodies were administered intraperitoneally (i.p.) on days 3, 7, and 11 (200 µg per dose). Tumors were excised on day 14 for further analysis. (b), NIR‐II fluorescence images showing platform accumulation and clearance in tumor regions of mice treated with PAPD alone or PAPD + αPD‐L1. Imaging was performed using 808 nm excitation, a 1300 nm LP filter, and a 200 ms fluorescence exposure time. Scale bar = 1 cm. (c,d), Quantitative analysis of (c) relative fluorescence intensity and (d) tumor area at days 1, 5, and 9 derived from the NIR‐II fluorescence imaging shown in panel (b). Regions of interest were selected at the tumor sites for fluorescence quantification. Data are presented as mean ± s.d. (*n* = 5 per group). Statistical significance between the PAPD (green) and PAPD + αPD‐L1 (pink) groups at each time point was determined using a two‐tailed Welch's *t*‐test. Exact P values: (c) day 1, NS, not statistically significant (P > 0.05); day 5, P = 3.82 × 10^−^^4^; day 9, P = 5.11 × 10^−^^7^; (d) day 1, NS, not statistically significant (P > 0.05); day 5, P = 1.41 × 10^−^^3^; day 9, P = 1.75 × 10^−^^4^. (e), Tumor growth curves of mice in different treatment groups (i: PBS, ii: αPD‐L1, iii: PAPD, and iv: PAPD + αPD‐L1) over 14 days. Data are presented as mean ± s.d. (*n* = 5 per group). Two‐way ANOVA revealed a significant effect of Group (F (3, 112) = 1297.4, P = 8.68 × 10^−^^8^ ^7^), Time (F (6, 112) = 734.5, P = 1.87 × 10^−^^8^ ^7^), and Group × Time interaction (F (18, 112) = 202.9, P = 1.16 × 10^−^^7^ ^6^). Post hoc pairwise comparisons between PBS and PAPD + αPD‐L1 were performed using two‐tailed Welch's *t*‐test, showing the most significant difference on day 13 (P = 1.18 × 10^−^^10^). (f), Images of excised tumors from all groups on day 14. (g), Ex vivo tumor weights at day 14 post‐treatment. Data are presented as mean ± s.d. (*n* = 5 per group). Statistical significance was determined using one‐way ANOVA, which revealed a significant overall difference among groups (F (3, 16) = 221.67, P = 3.07 × 10^−^^13^). Post hoc analysis was performed using Tukey's multiple comparison test (PBS vs. PAPD + αPD‐L1, P = 3.56 × 10^−13^; αPD‐L1 vs. PAPD+αPD‐L1, P = 6.63 × 10^−10^; PAPD vs. PAPD+αPD‐L1, P = 4.99 × 10^−4^). (h), Flow cytometry analysis of tumor‐infiltrating lymphocytes. Single‐cell suspensions were prepared from the tumor‐draining lymph nodes of C57BL/6 mice across four treatment groups on day 14 post‐treatment. CD3^+^ T cells and CD8^+^ T cells were evaluated via flow cytometry. Left: Representative flow cytometry dot plots for each group. Right: Quantification of CD8^+^ T cells as a percentage of CD3^+^ populations (mean ± s.d., *n* = 5 per group). Statistical significance was determined using one‐way ANOVA, which revealed a significant overall difference among groups (F (3, 16) = 160.9, P = 3.70 × 10^−^^1^ ^2^). Post hoc analysis was performed using Tukey's multiple comparison test, with the most significant difference observed between PBS and PAPD + αPD‐L1 (P = 3.68 × 10^−^^1^ ^3^). (i), Immunofluorescence analysis of intratumoral CD8^+^ T cell infiltration. Tumor tissue sections from C57BL/6 mice were collected on day 14 after treatment and subjected to immunofluorescence staining for CD8^+^ T cells (red). Nuclei were counterstained with DAPI (blue). Left: Representative immunofluorescence images of tumor tissues from each group. Scale bar, 20 µm. Right: Quantification of CD8^+^/DAPI‐positive cell ratios, calculated from five randomly selected fields (mean ± s.d., *n* = 5 per group). Statistical significance was determined using one‐way ANOVA, which revealed a significant overall difference among groups (F (3, 16) = 127.8, P = 2.17 × 10^−^^1^ ^1^). Post hoc analysis was performed using Tukey's multiple comparison test, with the most significant difference observed between PBS and PAPD+αPD‐L1 (P = 3.53 × 10^−^^1^ ^2^). (j), Immunofluorescence analysis of NK1.1^+^ cell infiltration in tumor tissues. Tumor sections from C57BL/6 mice were harvested on day 14 post‐treatment and stained for NK1.1^+^ cells (green). Nuclei were counterstained with DAPI (blue). Left: Representative immunofluorescence images showing NK1.1^+^ cell infiltration in tumor tissues. Scale bar, 20 µm. Right: Quantitative analysis of NK1.1^+^/DAPI‐positive cell ratios across different treatment groups based on five randomly selected fields (mean ± s.d., *n* = 5). Statistical significance was determined using one‐way ANOVA, which revealed a significant overall difference among groups (F (3, 16) = 399.0, P = 3.04 × 10^−^^1^ ^5^). Post hoc analysis was performed using Tukey's multiple comparison test, with the most significant difference observed between PBS and PAPD + αPD‐L1 (P = 2.22 × 10^−^^1^ ^6^).

To comprehensively assess the in vivo performance of the PAPD platform, a 14‐day therapeutic study was conducted using a murine MOC‐1 oral squamous cell carcinoma xenograft model. The full treatment schedule and timeline are illustrated in Figure 6a.

Throughout the treatment course, NIR‐II fluorescence imaging was performed at three time points to monitor platform accumulation and tumor progression in vivo. As shown in Figure 6b, mice treated with PAPD exhibited well‐defined fluorescence signals at the tumor sites, with gradually increasing intensity, indicating stable platform retention, and excellent signal‐to‐noise characteristics. The combination group (PAPD + αPD‐L1) displayed even stronger fluorescence signals compared to the PAPD‐only group, reflecting enhanced tumor accumulation and retention. Quantitative analysis of signal intensity (Figure 6c) confirmed this observation, demonstrating progressive enhancement in both groups. Notably, the combination group exhibited the most pronounced increase, suggesting a potential synergistic effect. Additionally, analysis of tumor fluorescence area (Figure 6d) showed a slight increase over time in the PAPD group, whereas the combination group exhibited a marked reduction in signal area, indicative of suppressed tumor growth. These findings support the use of PAPD not only for high‐contrast imaging but also as a potential real‐time indicator of therapeutic response.

Tumor volume was monitored throughout the treatment period across all four groups (Figure 6e). The combination therapy group (PAPD + αPD‐L1) exhibited the most pronounced tumor growth inhibition, followed by the PAPD and αPD‐L1 monotherapy groups, whereas rapid tumor progression was observed in the PBS control group. Body weight measurements remained stable across all groups (Figure S35), indicating the absence of overt systemic toxicity. At the end of the treatment cycle, excised tumors from the combination group were visibly smaller than those from the other groups (Figure 6f). Tumor mass quantification further confirmed the superior antitumor efficacy of the combined treatment (Figure 6g).

To further characterize the immunomodulatory effects of treatment, we first analyzed PD‐L1 expression in tumor tissues (Figure S36). All treatment groups exhibited increased PD‐L1 expression relative to the PBS control. While the αPD‐L1 monotherapy group showed only a moderate elevation, the PAPD group demonstrated a more pronounced increase. Strikingly, the combination group exhibited the highest PD‐L1 upregulation, underscoring the synergistic effect of mild photothermal stimulation and checkpoint blockade in remodeling the tumor microenvironment [41, 49]. Tumor‐draining lymph nodes were subsequently harvested for flow cytometric analysis. As shown in Figure 6h (with full datasets provided in Figure S37), the proportions of CD8^+^ T cells were significantly increased in both the PAPD and combination groups, with the highest levels observed in the combination group. This suggests that PAPD‐mediated photothermal stimulation enhanced antigen release and presentation, thereby promoting effector T cell priming and activation [7, 50].

Immunofluorescence staining of tumor tissues (Figure 6i–j) revealed markedly increased infiltration of CD8^+^ T lymphocytes in the PAPD and combination groups, corroborating their enhanced tumor‐specific killing capacity. In parallel, NK1.1^+^ cells were also more abundant in these groups, indicating improved activation of innate immunity (quantitative analysis was performed using five independent fields of view from each group, and all raw images are presented in Figures S38 and S39). Together, these data support that PAPD‐mediated mild photothermal stimulation, particularly when combined with PD‐L1 blockade, remodels the tumor immune microenvironment by promoting PD‐L1 expression, and enhancing infiltration of CD8^+^ T cells and NK1.1^+^ cells.

To further evaluate whether the increased lymphocyte infiltration was accompanied by enhanced cytotoxic effector activity, we performed additional immunofluorescence staining for Granzyme B (GZMB) and IFN‐γ in tumor sections. Compared with the PBS control group, both GZMB and IFN‐γ signals were markedly increased in the PAPD and combination groups, with the highest levels observed in the PAPD + αPD‐L1 group (Figures S40 and S41). These results are consistent with the increased CD8^+^ T‐cell and NK1.1^+^ cell infiltration observed in Figure 6i–j and support enhanced cytotoxic immune activity within the tumor microenvironment.

We further examined macrophage‐associated immune remodeling by immunofluorescence staining for CD86 and iNOS. The PAPD and PAPD + αPD‐L1 groups showed increased CD86^+^ and CD86^+^+iNOS^+^ signals in tumor tissues, with the most pronounced increase observed in the combination group (Figure S42). These findings suggest that PAPD‐mediated mild photothermal treatment also promotes a pro‐inflammatory macrophage phenotype, thereby contributing to broader remodeling of the tumor immune microenvironment.

Collectively, these in vivo results demonstrate that the PAPD platform enables high‐sensitivity NIR‐IIb tumor imaging, effective tumor growth suppression via mild PTT, and markedly enhanced antitumor efficacy when combined with PD‐L1 checkpoint blockade. The therapeutic synergy is associated with photothermally induced PD‐L1 upregulation and increased infiltration of CD8^+^ T cells and NK1.1^+^ cells, indicating effective remodeling of the tumor immune microenvironment. These findings establish PAPD as a versatile theranostic platform integrating imaging, therapy, and immunomodulation, with strong potential for clinical translation in precision oncology.

## 3 Conclusion

In this work, we developed a theranostic platform PAPD that integrates targeted molecular imaging with mild photothermal sensitized tumor immunotherapy. The platform was built on bandgap‐engineered Pt_x_Te_y_:Ag_2_Te composite QDs, in which programmable NIR‐II emission and controllable mild photothermal modulation underpin deep‐tissue imaging and immunotherapy sensitization. Antibody conjugation endowed PAPD with dual tumor‐targeting capability through EPR‐mediated passive accumulation and PD‐L1‐specific active recognition. Through mild photothermal modulation, PAPD upregulated tumor PD‐L1 expression, and reshaped the TIME, thereby enhancing the efficacy of immune checkpoint blockade and inducing pronounced antitumor immune activation. Overall, this work presents a versatile nanotheranostic platform that bridges deep‐tissue NIR‐II imaging and photothermal immunotherapy sensitization, providing a promising strategy for precision cancer diagnosis and treatment.

## 4 Methods

### 4.1 Synthesis of Pt_x_Te_y_:Ag_2_Te Composite Quantum Dots

To obtain Pt_x_Te_y_:Ag_2_Te, 0.067 g of AgAc (0.4 mmol), varying masses of PtCl_4_, 15mL of ODE, and 5 mL of OT were loaded into a 50 mL three‐neck flask and heated to 120°C under high‐purity argon gas flow. Subsequently, 1 mL of TBP‐Te (0.1 mmol) was injected into the reaction solution under stirring for 30 min. The products were then washed with acetone and centrifuged at 7000 rpm for 5 min. The resulting precipitate was dispersed in tetrachloroethylene for further characterization.

### 4.2 Water‐Dispersible Modification of Pt_x_Te_y_: Ag_2_Te

The prepared Pt_x_Te_y_:Ag_2_Te QDs were hydrophobic due to organic ligands on the surface. To render them hydrophilic, we employed amphiphilic polymers for hydrosoluble modification of the as‐prepared QDs. Oleylamine‐grafted polyacrylic acid (OIPA) was synthesized according to previously published methods, with details provided in the Supporting Information. For the synthesis of hydrophilic Pt_2_Te_3_:Ag_2_Te, the purified hydrophobic Pt_2_Te_3_:Ag_2_Te QDs (20 mg) and OIPA (50 mg) were dissolved in chloroform (50 mL) and sonicated for 30 min. The white powder gradually dissolved and the mixture transitioned to a clear brown solution. The chloroform was then removed by rotary evaporator. Subsequently, 20 mL of Britton‐Robinson (BR) buffer (0.05 M, pH = 12) was added to redisperse the product. Precipitates were removed by centrifugation at 7000 rpm for 15 min and excess OIPA micelles were separated by fast density gradient centrifugation.

### 4.3 Construction of the PAPD (Pt_2_Te_3_:Ag_2_Te‐αPD‐L1) Nanoplatform

The obtained Pt_x_Te_y_:Ag_2_Te‐OIPA QDs were first modified with NH_2_‐PEG‐NH_2_ for further PEGylation. 20 mg of the as‐prepared Pt_2_Te_3_:Ag_2_Te‐OIPA QDs, 30 mg of EDC·HCl and 30 mg of NH_2_‐PEG‐NH_2_ were dissolved in 20 mL of borate‐buffered saline (BBS) buffer (20 mm, pH = 7.4). The PEGylated QDs were obtained after shaking at room temperature for 2 h. The Pt_2_Te_3_:Ag_2_Te‐PEG QDs were purified by using a 100 kDa filter (Millipore) to remove excess PEG. The obtained PEG‐modified QDs were washed five times with 1 × PBS buffer and dispersed in 1 × PBS for storage at 4°C. 2 mg of Sulfo‐SMCC was dissolved in BBS buffer (0.05m, pH = 8.4) and then mixed with 1 mg Pt_2_Te_3_:Ag_2_Te‐PEG QDs, the activation was carried out for 1 h at 25°C, 150 rpm on a thermostatic oscillator. Then the activated Pt_2_Te_3_:Ag_2_Te‐PEG QDs with maleimide moieties were purified by ultrafiltration using 50 kDa ultrafiltration tubes and added to BBS buffer (0.05m, pH = 8.4). 3 mg DTT was dissolved in PBS buffer, and 1 mg α‐mouse PD‐L1 (B7‐H1) antibody was mixed with the DTT solution. The reaction was carried out at 150 rpm and 25°C for 0.5h. The reduced PD‐L1 antibody was purified by ultrafiltration with PBS. The activated Pt_2_Te_3_:Ag_2_Te‐PEG QDs were dispersed and mixed with the reduced PD‐L1 antibody in PBS buffer, and then reacted at a speed of 150 rpm and a temperature of 25°C for 1 h. The Pt_2_Te_3_:Ag_2_Te‐αPD‐L1 biofluorescence detection platform was concentrated and purified using 50 kDa ultrafiltration tubes. The PAPD was loaded onto a gravity‐driven size‐exclusion chromatography column after concentration and purification using an ultrafiltration tube. PBS was used as the eluent to separate unreacted QDs, yielding the purified PAPD.

### 4.4 NIR‐II Fluorescence Imaging

For in vitro imaging, centrifuge tubes and cell plates were placed in a dedicated NIR‐II small animal imaging system. Fluorescence images were acquired using LP filters. All samples were aligned under the same optical field to ensure image comparability. For in vivo imaging, SPF mice were intravenously injected with QD solution (dispersed in PBS). Following injection, mice were anesthetized using a gas anesthesia system with isoflurane, and positioned on the imaging stage. NIR‐II fluorescence imaging was performed using a 1300 nm LP filter and 808 nm excitation laser.

### 4.5 Cell Culture

Murine oral squamous carcinoma cell lines (MOC‐1 and MOC‐2) were purchased from Meisen CTCC, and human umbilical vein endothelial cells (HUVECs) were purchased from the American Type Culture Collection (ATCC). All cell lines were cultured under standard conditions. Cells were maintained in high‐glucose Dulbecco's Modified Eagle Medium (DMEM; Gibco, USA) supplemented with 10% fetal bovine serum (FBS; Gibco), 1% penicillin streptomycin (100 U/mL penicillin and 100 µg/mL streptomycin), and 1% L‐glutamine. Cells were incubated at 37°C in a humidified atmosphere containing 5% CO_2_.

When cultures reached approximately 80% confluence, cells were detached using 0.25% trypsin‐EDTA (Gibco), and neutralized with phosphate‐buffered saline (PBS). The resulting single‐cell suspensions were collected by centrifugation at 1000 rpm for 5 min, resuspended in fresh complete medium, and subcultured at a 1:3 ratio. All experiments were conducted using cells in the logarithmic growth phase and in good physiological condition to ensure experimental reproducibility and consistency.

### 4.6 Western Blot

MOC‐1 cells in the logarithmic growth phase were seeded into 6‐well plates (2 × 10^5^ cells per well) and divided into control and photothermal treatment groups. For the experimental group, cells were incubated with Pt_2_Te_3_:Ag_2_Te‐OIPA QDs (250 µg/mL) for 4 h, followed by irradiation with an 808 nm laser (0.84 W/cm^2^) for 20 min. Photothermal stimulation was performed twice at 24 h intervals to induce mild hyperthermia. Control cells were maintained under identical conditions without nanoparticle treatment or irradiation.

At 48 h after the initial treatment, cells were washed twice with cold phosphate‐buffered saline (PBS) and lysed on ice using RIPA buffer (Beyotime, China) containing protease and phosphatase inhibitors for 30 min. Cell lysates were centrifuged at 12 000 × g for 10 min at 4°C, and supernatants were collected for protein quantification using a BCA Protein Assay Kit (Thermo Fisher Scientific). Equal amounts of total protein (30 µg) were resolved by SDS‐PAGE and electrotransferred onto polyvinylidene difluoride (PVDF) membranes (Millipore). Membranes were blocked with 5% nonfat milk in TBST (20 mm Tris, 150 mm NaCl, 0.1% Tween‐20, pH 7.4) for 1 h at room temperature, followed by overnight incubation at 4°C with primary antibodies against PD‐L1 (Abcam, 1:1000) and GAPDH (Abclonal, 1:10 000) as a loading control. After washing three times with TBST, membranes were incubated with HRP‐conjugated secondary antibodies (1:10 000, CST) for 1 h at room temperature. Protein bands were visualized using an enhanced chemiluminescence (ECL) detection kit (Thermo Fisher Scientific) and imaged with a ChemiDoc MP Imaging System (Bio‐Rad, USA). Densitometric quantification of band intensities was performed using ImageJ software (NIH, USA).

### 4.7 Animals

Female C57BL/6 mice (4–6 weeks old, 16–18 g) were purchased from GemPharmatech Co., Ltd. (Jiangsu, China), and female BALB/c nude mice (4–6 weeks old, 14–16 g) were purchased from Vital River Laboratory Animal Technology Co., Ltd (Beijing, China). All mice were maintained in a specific pathogen‐free (SPF) facility at the Hangzhou Institute of Medicine, Chinese Academy of Sciences. All animal procedures were approved by the Institutional Animal Care and Use Committee of the Hangzhou Institute of Medicine (Ethics Approval No. AP2024‐08‐0158) and performed in accordance with institutional guidelines. Humane endpoints were applied throughout, and mice were euthanized by CO_2_ asphyxiation at the conclusion of experiments.

### 4.8 H&E Staining

Organ or tumor tissues were harvested and immediately fixed in 4% paraformaldehyde solution for no less than 24 h. The fixed tissues were subsequently dehydrated, cleared, embedded in paraffin, and sectioned into thin slices. H&E staining was performed, where hematoxylin was used to stain nuclei and eosin to stain the cytoplasm. After dehydration and mounting, histological analysis was conducted using an optical microscope.

### 4.9 Immunohistochemistry (IHC)

Tumor tissues were fixed in 4% paraformaldehyde for at least 24 h, followed by paraffin embedding, sectioning, and mounting on slides. After deparaffinization and hydration, antigen retrieval was performed in citrate buffer using a heat‐mediated method. Endogenous peroxidase activity was blocked with 3% hydrogen peroxide for 10 min. Sections were then incubated with primary antibodies overnight at 4°C, followed by HRP‐conjugated secondary antibody incubation and DAB visualization. Hematoxylin was used for nuclear counterstaining. Images were captured using a bright‐field optical microscope. The primary antibodies used in this study were αPD‐L1 for evaluating immune marker expression in tumor tissues.

### 4.10 Immunofluorescence Staining

Tumor tissues were fixed in 4% paraformaldehyde for at least 24 h, followed by routine paraffin embedding, sectioning, and mounting on slides. After deparaffinization and hydration, antigen retrieval was performed using heat‐mediated treatment in citrate buffer (pH 6.0). Endogenous peroxidase activity was blocked with 3% hydrogen peroxide, and nonspecific binding sites were blocked with 5% normal goat serum. Slides were incubated overnight at 4°C with primary antibodies to assess cell proliferation and immune cell infiltration. For apoptosis analysis, TUNEL staining was performed using a commercial kit according to the manufacturer's instructions. On the following day, slides were washed and incubated with appropriate Alexa Fluor‐conjugated secondary antibodies for 1 h at room temperature in the dark. Nuclei were counterstained with DAPI. After mounting, images were acquired using a fluorescence or confocal microscope. Quantitative analysis of fluorescence intensity or the ratio of marker‐positive cells was performed using ImageJ or Image Pro Plus software.

### 4.11 Proteomic Analysis

MOC‐1 tumor cells were seeded in 6‐well plates and treated with Pt_2_Te_3_:Ag_2_Te‐OIPA QDs (250 µg/mL) for mild photothermal stimulation. The cells were exposed to an 808 nm laser for 20 min, maintaining the temperature between 40°C–45°C. The treatment was performed twice at 24 h intervals. Control cells were cultured under identical conditions but without nanoparticle addition or laser irradiation. After the final treatment, cells were washed twice with cold PBS and lysed using RIPA buffer (containing protease and phosphatase inhibitors) on ice for 30 min. The lysates were centrifuged at 12000 × g for 15 min at 4°C to remove debris, and the supernatants were collected for protein quantification using the BCA assay.

Equal amounts of protein from each sample were reduced, alkylated, and digested with trypsin overnight at 37°C. The resulting peptides were desalted using C18 spin columns, vacuum‐dried, and reconstituted in 0.1% formic acid for LC‐MS/MS analysis. The digested peptides were analyzed on a high‐resolution mass spectrometer (Thermo Scientific Orbitrap Astral) coupled with a nanoLC system. All raw DIA mass spectrometry data were processed and integrated using DIA‐NN software for database searching and quantitative proteomic analysis. Differentially expressed proteins were identified based on a fold‐change threshold (|log_2_FC| > 1) and adjusted *p* < 0.05. Bioinformatic analyses, including GO, KEGG, and GSEA enrichment, were performed to explore functional pathways associated with mild photothermal stimulation.

### 4.12 Flow Cytometry Analysis

#### 4.12.1 In Vitro Detection of PD‐L1 Expression

To evaluate the effect of mild photothermal treatment on PD‐L1 expression in tumor cells, MOC‐1 cells were seeded in 6‐well plates and treated with 250 µg/mL of Pt_2_Te_3_:Ag_2_Te QDs. The cells were irradiated with an 808 nm laser for 20 min to maintain the temperature at 40°C–45°C, once per day for two consecutive days. Control cells were treated with culture medium alone. After treatment, cells were collected, washed with PBS, and incubated with fluorescently labeled αPD‐L1 antibody at 4°C for 30 min in the dark. After washing, PD‐L1 expression was analyzed using flow cytometry.

#### 4.12.2 Detection of CD8^+^ T Cells in Draining Lymph Nodes

To assess the immune response following combination therapy, inguinal draining lymph nodes were harvested from mice after treatment. The tissues were gently ground through a 70 µm cell strainer using a sterile syringe plunger to obtain single‐cell suspensions. After red blood cell lysis and PBS washing, cells were incubated with fluorophore‐conjugated anti‐mouse CD8 antibody for 30 min at 4°C in the dark. After washing, the proportion of CD8^+^ T cells was analyzed by flow cytometry.

### 4.13 In Vivo Antitumor Study in MOC‐1 Tumor‐Bearing Mice

To establish the subcutaneous tumor model, 5 × 10^5^ MOC‐1 cells suspended in 150 µL culture medium were injected into the dorsal flank of 4‐week‐old female C57BL/6 mice. Once the tumor volume reached 100–150 mm^3^, mice were randomly divided into four groups (*n* = 5 per group) and received treatments as illustrated in Figure 6a. For the PAPD + αPD‐L1 and PAPD alone groups, NIR‐II fluorescence imaging was conducted on days 1, 5, and 9 post‐treatment. 4 h after intravenous injection of the PAPD (200 µL, 5 mg/mL), mice were anesthetized and imaged using a NIR‐II imaging system under 808 nm laser excitation (80 mW/cm^2^), with a 1300 nm LP filter and 200 ms exposure time. Immediately following imaging, tumors were irradiated with an 808 nm laser at a power density of 0.64 W/cm^2^ for 20 min. αPD‐L1 antibody (200 µg/mouse) was administered intraperitoneally on days 3, 7, and 11 in both the combination group (PAPD + αPD‐L1) and the αPD‐L1 monotherapy group. The PBS control group received PBS injection at the corresponding time points.

Tumor volumes and body weights were recorded every other day from day 1 until the end of the study. Tumor volume was calculated using the formula: volume = length × width^2^ × 0.5. On day 14, all mice were euthanized. Tumors were excised and weighed, and tumor‐draining lymph nodes were collected and processed into single‐cell suspensions for flow cytometric analysis of CD8^+^ T cells. Tumor tissues were also harvested for immunofluorescence analysis of CD8^+^ T cells, NK1.1^+^ cells, and PD‐L1 expression to evaluate immune activation within the tumor microenvironment.

### 4.14 Statistical Analysis

Data are presented as mean ± standard deviation (s.d.) as indicated. For normally distributed data sets with equal variances, one‐way ANOVA followed by Tukey's post‐hoc test was used to compare multiple groups. For experiments involving two factors, two‐way ANOVA followed by Tukey's post‐hoc test was applied to assess the effects of treatment and time. Unpaired Student's *t*‐tests were used for comparisons between two groups where applicable. In all analyses, statistical significance was defined as P ≤ 0.05. Exact P values are reported in the figure legends. Statistical analyses were performed using Python 3.11 with the statsmodels library.

### 4.15 Schematics

Schematics were created with Adobe Illustrator.

## Author Contributions

J.L., R.X., Z.‐Q.T., and H.W. conceived the project and designed the experiments. J.L., Q.W., and Y.‐W.Z. synthesized materials. J.L. and R.X. performed in vitro cell experiments. J.L., R.X., Q.W., T.‐X.X., Y.‐W.Z., and L.D. contributed to data collection and analysis. J.L., R.X., L.‐X.Z., L.S., and T.‐X.X. performed in vivo experiments. J.L., R.X., Z.‐Q.T., and H.W. cowrote the manuscript. H.W., Z.‐Q.T., and R.X. acquired funding. All authors discussed the results and reviewed the manuscript.

## Conflicts of Interest

The authors declare no conflicts of interest.

## Supporting information




**Supporting File**: advs75777‐sup‐0001‐SuppMat.docx.

## Data Availability

Data Availability Statement.
