# Supplementary material for: Bandgap‐Engineered PtxTey:Ag2Te Composite Quantum Dots Enable Programmable NIR‐II Imaging and Photothermal‐Immune Synergistic Therapy
Source: Adv Sci (Weinh). 2026 May 20:e75777. Online ahead of print. doi: 10.1002/advs.75777 (PMC13335840; doi:10.1002/advs.75777)
Supplement: Supplementary file 1 — Supporting File: advs75777‐sup‐0001‐SuppMat.docx. [file ADVS-9999-e75777-s001.docx]

Bandgap-Engineered Pt_x_Te_y_:Ag_2_Te Composite Quantum Dots Enable Programmable NIR-II Imaging and Photothermal-Immune Synergistic Therapy

Jing Liu ^1,2^^,4^, Rui Xu ^3,4^ ***^*^***, Quan Wu ^2^, Tian-Xin Xiao ^1^, Lai-Xi Zhao ^3^, Lei Sun ^3^, Ya-Wen Zheng ^2^, Liang Dong ^1^, Zhi-Quan Tian ^2^ ***^*^*** and Haibo Wang ^1^ ***^*^***

^1^ Zhejiang Cancer Hospital, Hangzhou Institute of Medicine, Chinese Academy of Sciences, Hangzhou, 310000, China.

*^2^* College of Chemistry and Molecular Sciences, Wuhan University, Wuhan 430072, China.

*^3^* College & Hospital of Stomatology, Anhui Medical University, Anhui Province Key Laboratory of Oral Diseases Research, Hefei, 230032, China.

*^4^ These authors contributed equally: Jing Liu, Rui Xu.*

**^*^Corresponding Author**

Rui Xu ([dentistxr@fy.ahmu.edu.cn](mailto:dentistxr@fy.ahmu.edu.cn)**)**, Zhi-Quan Tian [(zqtian@whu.edu.cn),](mailto:(zqtian@whu.edu.cn),) Haibo Wang ([wanghaibo@him.cas.cn](mailto:wanghaibo@him.cas.cn))

**Table of contents**

**Supplementary Methods** **.............................................................................................................. S2**

**Supplementary Figures ................................................................................................................ S9**

**Supplementary Tables ................................................................................................................. S52**

**Supplementary References ......................................................................................................... S56**

**Supplementary methods**

**Materials**

Silver acetate (AgAc) was purchased from Shanghai Macklin Biochemical Co., Ltd. 1-octadecene (ODE,tech.90%)，tri-n-butylphosphine (TBP, tech.95%), 1-Octanethiol (OT,tech.98%), tellurium powder (Te, ≥99.99 %), polyacrylic acid (PAA,M.W~2000), oleylamine (OAM, 80-90%), Sodium 4-(N-maleimidomethyl) cyclohexane-1-carboxylate-3-thiosuccinimidyl ester (Sulfo-SMCC) and 1,4-Dithiothreitol (DTT) were purchased from Aladdin Reagent (Shanghai) Co., Ltd. Diamino Polyethylene glycol (NH_2_-PEG-NH_2_, MW: 3400) was purchased from Ponsure Biological. N-hexane, acetone, ethanol, dimethyl formamide (DMF), tetrachloroethylene, hydrochloric acid, chloroform, borax, boric acid and agarose were purchased from China National Pharmaceutical Group Corporation. N-(3-Dimethylaminopropyl)-N-ethyl carbodiimide hydrochloride (EDC·HCl) was purchased from Shanghai Medpep Co., Ltd. Platinum tetrachloride (≥99.9%, trace metals basis) was purchased from Sigma-Aldrich. Iohexol (60%) was purchased from Yuan pei Biotechnology Co., Ltd. (Shanghai, China). Phosphate-buffered saline (1 × PBS), 4% paraformaldehyde and 50 × TAE buffer was obtained from Servicebio (Wuhan, China). Dulbecco’s Modified Eagle Medium (DMEM), fetal bovine serum (FBS), trypsin-EDTA, and penicillin-streptomycin were purchased from Gibco (Thermo Fisher Scientific, USA). Isoflurane was obtained from Veteasy (Beijing, China). Polypropylene chromatography columns were purchased from PIERCE. Superdex 200 gel filtration media and NAP™-10 desalting columns were obtained from GE Healthcare. Ultrafiltration devices (Amicon Ultra-4, 50 kDa molecular weight cut-off) were purchased from Millipore. α-mouse PD-L1 (B7-H1) antibody (Cat# BE0101) was purchased from Bio X Cell (Lebanon, NH, USA). The PD-L1 antibody was purchased from Abcam (Cat# ab213480) and used for Western blot analysis at a dilution of 1:1000. Antibodies for Flow Cytometry: PE anti-mouse CD274 (B7-H1, PD-L1) antibody (Cat# 124308, BioLegend) was used for detecting PD-L1 expression. Anti-mouse CD3 (clone 17A2), BV605-conjugated (BioLegend, Cat# 100237), and anti-mouse CD8a (clone 53-6.7), FITC-conjugated (BioLegend, Cat# 100706) were employed for phenotyping CD8⁺ T cells isolated from tumor-draining lymph nodes.

**Synthesis of Ag₂Te quantum dots**

Tellurium precursor (TBP-Te): 1mmol (0.128 g) Te was dissolved in 10mL of TBP at 25 °C under sonicating for 15 minutes (min). The gray tellurium powder gradually dissolved and a clear colorless solution was obtained as the TBP-Te^[1]^.

Synthesis of Ag_2_Te quantum dots (QDs): In a typical synthesis reaction of Ag_2_Te QDs, 0.067g of AgAc (0.4mmol), 15mL of ODE and 5mL of OT were loaded in a 50mL three-neck flask under the high purity argon gas flow. The mixture was stirred and heated to 120 °C to obtain clarified light-yellow solution. Subsequently, 1 mL (0.1mmol) of TBP-Te was injected into the reaction solution under uniform stirring, the color of the mixture changed from clarified light-yellow to black. Ag_2_Te QDs were obtained by reacting for 30 min under these conditions. Then the products were mixed with acetone and centrifugated with 7000 rpm for 10 min to obtain a clearer product. The resulting precipitate was dispersed in tetrachloroethylene for further characterizations.

**Synthesis of OIPA**

10 mL of DMF was added to a 50 mL round bottom flask. 1.008 g of PAA and 1.342 g of EDC·HCl were weighed into the flask and dissolved with stirring at ambient temperature. After the solution is homogeneous and transparent, 1.42 mL of oleylamine was added to the system slowly dropwise. The reaction was stirred with 700 rpm/min at 38 °C for 12 hours. The solution was removed from DMF by vacuum evaporation after the reaction was completed. The liquid removed from DMF was dissolved with acetone and washed with 1M HCl to obtain a white solid. Then the white solid was dissolved in ethanol and washed with 1M HCl again, repeat three times. Finally, dissolve the white solid with ethanol and spin dry with rotary evaporator under vacuum to obtain the white powder as OIPA^[2]^.

**Instrumental characterization techniques**

Transmission electron microscopy (TEM) samples were prepared by dropping a diluted QDs in n-hexane onto Cu grids with carbon support film. We use FEI Talos F200X TEM (FEI Company, USA) at an accelerating voltage of 200kV to acquire the size, morphology and crystalline structure images of QDs. Energy-dispersive X-ray spectroscopy (EDS) measurements were performed simultaneously with TEM imaging by initiating electron beam scanning and collecting the EDS spectra. Images of element mapping and high-angle annular dark-field mode (HAADF-STEM) were examined through JEM ARM 200F (JEOL Japan Electronics) TEM operated at 200 kV equipped with automatic aberration correction system. X-ray photoelectron spectroscopy (XPS) measurements were performed using Thermo ESCALAB 250Xi (ThermoFisher Scientific, USA) with A1 target (1486.6 eV) as the excitation source, calibrated with C1s electron binding energy (284.84 eV). Ultroviolet Photoelectron Spectrometer (UPS) information was obtained on an electron spectrometer under 5 V bias. Samples for XPS and UPS were prepared on the silicon slice via dropping the QD solution onto it. UV-Vis-NIR diffuse reflectance spectroscopy (UV-Vis-NIR DRS) of hydrophobic quantum dots was performed using a UV-3600 spectrophotometer (Shimadzu, Japan) equipped with an integrating sphere. Solid QD samples obtained by rotary evaporation were pressed into the micro-sample holder, and spectra were recorded in the 200–2500 nm range. Fourier transform infrared (FTIR) spectroscopy was performed on liquid QD samples using an IRTracer-100 spectrometer (Shimadzu, Japan). Samples were directly measured in liquid form, and spectra were recorded over the range of 4000–400 cm^-1^. Powder X-ray diffraction (XRD) diagrams were acquired by Bruka D8 Advanced X-ray powder diffraction (Bruker, Germany) with a scan range of 20°–80° and a scan speed of 6°/min. UV absorption spectra were collected with UV-3600 UV-vis-NIR spectrophotometer (Shimadzu, Japan). A Fluorolog-3 fluorescence spectrophotometer (HORIBA, Jobin Yvon Inc.) equipped with an InGaAs detector was used to acquire NIR fluorescence emission spectra. All the samples were dispersed in tetrachloroethylene and excited by an 808 nm laser at room temperature. Transient fluorescence spectra and absolute quantum yield measurements of quantum dots were performed using purified QD samples dispersed in tetrachloroethylene. The solutions were diluted to an appropriate concentration and loaded into an infrared quartz cuvette. A QuantaMaster^TM^8000 spectrofluorometer equipped with an integrating sphere was employed to measure the absolute quantum yield and transient fluorescence spectra, with excitation provided by an 808 nm laser. Inductively coupled plasma-atomic emission spectroscopy (ICP-AES) for elemental analysis was examined on IRIS Intrepid П XSP type full spectrum (Thermoelectric Elements Inc, USA). The hydrated particle size and zeta potential were measured by Malvern Zetasizer Nano ZS nanoparticle size and surface potential analyzer (Malvern Instruments Ltd). Ultracentrifuge (Beckman Coulter Optima MAX-XP, MLA-130) was used for ultracentrifugation by using iodixanol as the gradient density centrifugation solution. NIR-II fluorescence and in vivo images were acquired using a NIR-II small animal imaging system (VanGogh IGS1000, Suzhou Yingrui Optical Technology Co., Ltd., China) equipped with an 808 nm fiber-coupled diode laser as the excitation source.

**Systemic toxicity and histopathological evaluation**

15 SPF C57BL/6 mice (4 weeks old) were randomly assigned into five groups (n = 3 per group), including a PBS control group and four experimental groups. Mice in the experimental groups received 200 μL of Pt_2_Te_3_:Ag_2_Te-OIPA QDs (5 mg/mL) via tail vein injection, while the control group was administered an equal volume of PBS. Mice were sacrificed at days 1, 3, 7 and 14 post injection, with three mice euthanized at each time point. At each designated time point, approximately 1 mL of whole blood was collected per mouse. Half (0.5 mL) was transferred into anticoagulant tubes for complete blood count (CBC), and the remaining 0.5 mL was collected in standard centrifuge tubes for serum separation and biochemical analysis. After blood collection, mice were immediately euthanized, and major organs including the liver, heart, lungs, spleen, and kidneys were harvested for histopathological evaluation. Tissues were fixed in 4% paraformaldehyde, embedded in paraffin, sectioned, and stained with hematoxylin and eosin (H&E). The PBS control group and day-1 experimental group were processed in parallel to ensure consistency. Hematological analysis was conducted using an automated veterinary hematology analyzer (BC-30Vet, Mindray). For biochemical assays, blood samples were allowed to clot at room temperature for 2 h and then centrifuged at 1500 × g for 10 min at 4 °C. The serum supernatant was collected and analyzed using a fully automated biochemical analyzer (Chemray 240, Shenzhen Rayto Life and Analytical Sciences Co., Ltd) to evaluate hepatic and renal function^[3,4]^. These assessments were used to systematically evaluate the in vivo biocompatibility and systemic safety of the quantum dots.

**Cytotoxicity assessment in normal endothelial cells using the CCK-8 assay**

To evaluate the cytotoxicity of Pt_2_Te_3_:Ag_2_Te QDs in normal cells, a standard CCK-8 assay was performed on human umbilical vein endothelial cells (HUVECs). Cells in logarithmic growth phase were harvested, counted, and seeded into 96-well plates at a density of approximately 1 × 10⁴ cells per well in 100 μL of complete medium. After overnight incubation at 37 °C with 5% CO₂ to allow for cell adherence, cells were treated with Pt_2_Te_3_:Ag_2_Te-OIPA QDs at varying concentrations (0, 25, 50, 100, 150, 200, 250, 300, 500, and 1000 μg/mL). Each condition was tested in triplicate. After 24 h of incubation, 10 μL of CCK-8 solution was added to each well and cells were incubated for an additional 1 h. Absorbance at 450 nm was measured using a microplate reader to determine cell viability.

**Photothermal performance evaluation**

In vitro conventional photothermal performance. To assess the conventional photothermal properties of Pt₂Te₃:Ag₂Te-OIPA QDs, 0.2 mL of QD solutions at varying concentrations (0, 1, 3, and 5 mg/mL) were placed in 0.2 mL centrifuge tubes and irradiated with an 808 nm laser (beam diameter: 1.5 cm, power density: 0.28 W/cm²) for 5 min. Temperature changes were monitored using an infrared thermal camera, and photothermal images were captured. To evaluate photothermal stability, QD solutions at 1, 3, and 5 mg/mL were subjected to five heating-cooling cycles under the same irradiation conditions (808 nm, 0.28 W/cm², 5 min), with samples allowed to return to room temperature between cycles.

In vivo conventional photothermal evaluation. BALB/c-nu mice (4 weeks old) were subcutaneously inoculated with 1 × 10⁶ MOC-1 tumor cells in 150 μL culture medium. When tumor volumes reached 100–150 mm³, 30 μL of Pt₂Te₃:Ag₂Te-OIPA QDs (3 mg/mL) were intratumorally injected. Under isoflurane anesthesia, tumors were irradiated with an 808 nm laser (beam diameter: 1.0 cm, power density: 0.013 W/cm²) for 5 min. Thermal images were acquired using an infrared camera. Tumors in the control group were injected with 30 μL PBS.

In vitro mild photothermal performance. To characterize mild photothermal behavior, 0.2 mL of Pt₂Te₃:Ag₂Te-OIPA QDs at 250 μg/mL were irradiated in centrifuge tubes under an 808 nm laser (beam diameter: 1.5 cm) at 0.32, 0.48, and 0.72 W/cm² for 20 min. Additionally, 1 mL of the same solution was placed in 12-well plates and irradiated with a 2 cm beam diameter under power densities of 0.32, 0.60 and 0.84 W/cm². PBS was used in control groups. Photothermal stability under mild conditions was assessed by performing five cycles of heating (808 nm, 0.84 W/cm², 5 min) followed by cooling to room temperature. Thermal profiles were recorded using an infrared thermal imager.

In vivo mild photothermal evaluation. For in vivo mild photothermal assessment, tumor-bearing BALB/c-nu mice received 60 μL intratumoral injections of 250 μg/mL QDs. Tumors were then irradiated using an 808 nm laser (power density: 0.64 W/cm²) for 20 min. Thermal images were recorded at different time points. Control mice received equal volumes (60 μL) of PBS.

**NIR-II fluorescence imaging for evaluating targeting ability**

In vitro NIR-II imaging. To evaluate the targeting specificity of PAPD, VE and OE MOC-2 cells were seeded in 24-well plates and incubated with 250 μg/mL PAPD at 4 °C for 2 h. After incubation, cells were washed twice with PBS and imaged using a small-animal NIR-II imaging system under 808 nm laser excitation (80 mW/cm²), 1300 nm LP filter, and 500 ms exposure time. Control cells were incubated with Pt₂Te₃:Ag₂Te QDs under the same conditions.

In vivo NIR-II imaging (tumor accumulation and dynamics). C57BL/6 female mice (4 weeks old) were subcutaneously inoculated with 5 × 10^5^ MOC-1 cells in 150 μL medium. When tumor volume reached 100–150 mm³, mice were anesthetized with isoflurane and injected via tail vein with 200 μL of PAPD (5 mg/mL). Fluorescence images were collected at 0, 0.02, 1, 2, 4, 8, 24, and 48 h post-injection using the NIR-II imaging system (808 nm laser, 80 mW/cm², 1300 nm LP filter, 200 ms exposure). Control mice received Pt₂Te₃:Ag₂Te QDs.

In vivo imaging for OE/VE tumor-bearing mice. To assess PD-L1-targeted imaging, two groups of mice were subcutaneously injected with OE or VE MOC-2 cells, respectively. When tumor volume reached 100–150 mm³, each mouse was intravenously administered 200 μL of PAPD (5 mg/mL). At 4 h post-injection, mice were anesthetized and subjected to NIR-II imaging under the same conditions. Tumors were excised post-mortem and ex vivo fluorescence imaging was performed.

**Agarose gel electrophoresis**

To prepare the agarose gel, 150 mg of agarose was dissolved in 15 mL of 0.5 × TAE buffer by microwave heating until fully liquefied. The solution was then poured into a gel casting mold and allowed to cool at room temperature to form a 1% agarose gel. The solidified gel was placed into an electrophoresis chamber (DYY-Ⅲ-8B model), and approximately 1 L of 0.5 × TAE buffer was added as the running buffer. Sample solutions were mixed with glycerol at a volume ratio of 4:3 to increase density, then carefully loaded into the wells of the agarose gel. Electrophoresis was performed under a constant voltage of 120 mV for 30 minutes.

**Determination of fluorescence lifetime**

Under 808 nm laser excitation, the time-resolved fluorescence decay curves of Ag₂Te and Pt₂Te₃:Ag₂Te composite quantum dots were recorded. The fluorescence lifetimes were obtained by fitting the decay curves using a biexponential function, according to equations (1-1)^[5,6]^.

$\text{I}\left( \text{t} \right)\text{=}\text{A}_{\text{1}}\exp\left( \text{-}\frac{\text{τ}}{\text{τ}_{\text{1}}} \right)\text{+}\text{A}_{\text{2}}\text{exp(-}\frac{\text{τ}}{\text{τ}_{\text{2}}}\text{)}$ （1-1）

Here, I(t) represent the fluorescence intensity as a function of time t; A₁ and A₂ are the exponential amplitudes corresponding to the initial fluorescence intensities; τ₁ and τ₂ are the two lifetime constants, where τ₁ denotes the fast decay component, typically associated with nonradiative recombination processes, and τ₂ describes the slow decay component, which is usually attributed to recombination processes occurring within the sample.

**Photothermal conversion efficiency calculation of Pt₂Te₃:Ag₂Te-OIPA QDs**

To evaluate the photothermal performance of Pt₂Te₃:Ag₂Te-OIPA QDs, an aqueous dispersion (3 mg/mL, 1 mL) was exposed to an 808 nm laser with a power density of 0.28 W/cm². The temperature variation during irradiation was recorded every 30 seconds using an infrared thermal camera until the solution reached a steady-state temperature. The laser was then turned off, and the solution was allowed to cool naturally to ambient temperature^[7]^.

The photothermal conversion efficiency (*η*) was calculated using the following equation:

$\text{η}\text{=}\frac{\text{hs}\text{(}\text{T}_{\text{max}}\text{-}\text{T}_{\text{surr}}\text{)-}\text{Q}_{\text{dis}}}{\text{I}\text{(1-}\text{10}^{\text{-}\text{A}_{\text{λ}}}\text{)}}$ （1-2）

Where *Tₘₐₓ* is the maximum steady-state temperature; *Tₛᵤᵣᵣ* is the ambient temperature; *h* is the heat transfer coefficient; *s* is the surface area of the vessel; *Q_dis_* represents the heat dissipation caused by light absorption of the container and solvent; $\text{A}_{\text{λ}}$is the absorbance value of the Pt₂Te₃:Ag₂Te-OIPA QDs at 808 nm and *I* is the laser power density.

*θ* is a dimensionless driving force temperature parameter defined as:

$\text{θ}\text{=}\frac{\text{T}\text{-}\text{T}_{\text{surr}}\text{(Δ}\text{T}\text{)}}{\text{T}_{\text{max}}\text{-}\text{T}_{\text{surr}}}$ （1-3）

*τₛ* is defined as the time constant of the sample system, the time constant *τₛ* was calculated according to the following equation:

$\text{τ}_{\text{s}}\text{=}\frac{\text{t}}{\text{-}\text{lnθ}}$ （1-4）

*hs* value can be calculated by the following equation:

$\text{hs}\text{=}\frac{\text{M}_{\text{d}}\text{C}_{\text{d}}}{\text{τ}_{\text{s}}}$ （1-5）

while *M_d_* and *C_d_* correspond to the mass (1 g) and specific heat capacity (4.2 J· g⁻¹ ·°C⁻¹)

of water, respectively. The system heat loss *Q_dis_* was calculated as follows:

$\text{Q}_{\text{dis}}\text{=-}\text{∑}_{\text{i}}\text{m}_{\text{i}}\text{c}_{\text{p}\text{,}\text{j}}\frac{\text{dT}}{\text{dt}}$ （1-6）

The photothermal conversion efficiency (PCE) can be calculated after measuring the absorbance value ($\text{A}_{\text{λ}}$).

**HOMO calculation**

The HOMO energy level of the product was evaluated based on the ultraviolet photoelectron spectroscopy (UPS) results using Equation (1-7). The HOMO energy is calculated as the difference between the photon energy of the ultraviolet light and the kinetic energy range required for electrons to escape from the sample surface, which corresponds to the interval between the onset energy and the cutoff energy in the UPS spectrum^[8,9]^.

$\text{E}_{\text{HOMO}}\text{=}\text{h}\text{ν}\text{-(}\text{E}_{\text{cutoff}}\text{-}\text{E}_{\text{onset}}\text{)}$ （1-7）

HOMO refers to the energy level of the highest occupied molecular orbital. Here, *h*$\text{ν}$ is the photon energy of the ultraviolet light source; *E_cutoff_* represents the secondary electron cutoff energy in the UPS spectrum, while *E_onset_* denotes the onset energy at which photoelectrons begin to escape from the material surface. The HOMO energy level is therefore calculated as the difference between the photon energy and the kinetic energy range.

**UV-Vis-NIR DRS and calculation of bandgap**

Quantum dot samples were filled into micro sample tanks and tested for UV-visible diffuse reflectance with an integrating sphere. The UV-visible diffuse reflectance was performed by the following equation^[10,11]^.

$\left( \text{α}\text{hν} \right)^{\text{1/}\text{m}}\text{=}\text{B}\text{ (}\text{hν}\text{-}\text{E}_{\text{g}}\text{)}$ （1-8）

*α*: absorption coefficient, *h*: Planck's constant, ν: photon frequency, *hν*: photon energy, *E_g_* is the semiconductor forbidden bandwidth and *B* is a constant. Where, *m* is related to the semiconductor type, *m* is taken as 1/2 for direct band gap semiconductors and *m* is taken as 2 for indirect band gap semiconductors. The obtained UV spectral data were used to calculate *ν*, *hν*, *αhν*, and *(αhν)^1/m^* in turn, and graphing with *hν* and *(αhν)^1/m^*. Take the straight-line part of the curve and extrapolate it to the X-axis to get the bandgap *E_g_*.

**Supplementary Figures**


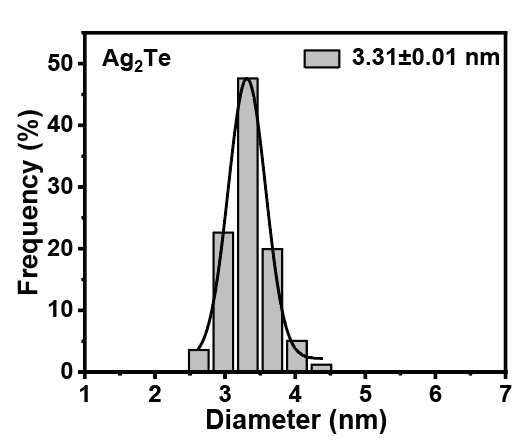


**Supplementary Fig. 1 | Structural, optical, and theranostic evaluation of Pt₂Te₃:Ag₂Te composite QDs.** Size distribution histogram of Ag_2_Te QDs based on TEM analysis. The particle diameters were measured and fitted with a Gaussian distribution, revealing an average size of 3.31 ± 0.01 nm (n≥300).


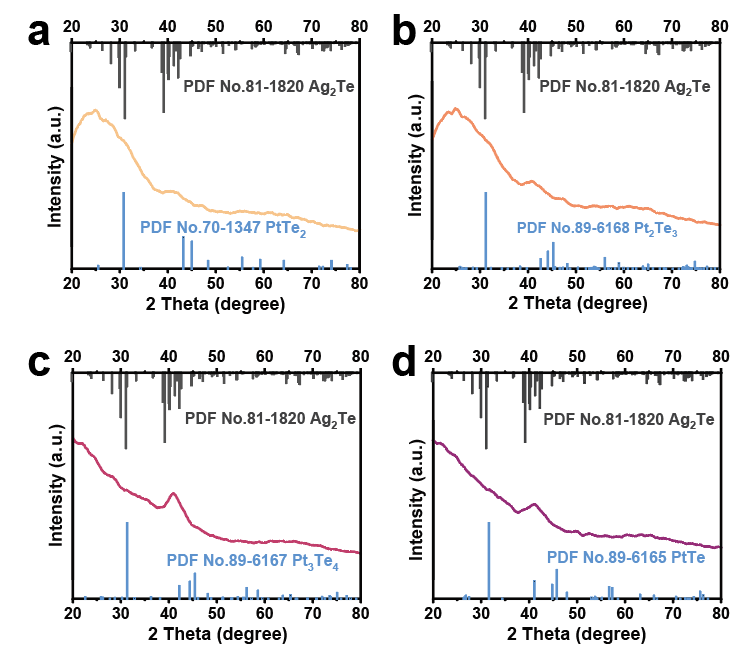


**Supplementary Fig. 2 |** XRD spectrum of Ag_2_Te and Pt_x_Te_y_:Ag_2_Te QDs. XRD spectra of Ag_2_Te QDs and a series of Pt_x_Te_y_:Ag_2_Te QDs with different Pt/Te compositions. **a**, Ag_2_Te and PtTe₂ (PDF No.70-1347). **b**, Ag_2_Te and Pt₂Te₃ (PDF No.89-6168). **c**, Ag_2_Te and Pt₃Te₄ (PDF No.89-6167). **d**, Ag_2_Te and PtTe (PDF No.89-6165). All samples indicate the successful incorporation of platinum and the formation of various platinum telluride phases during synthesis.


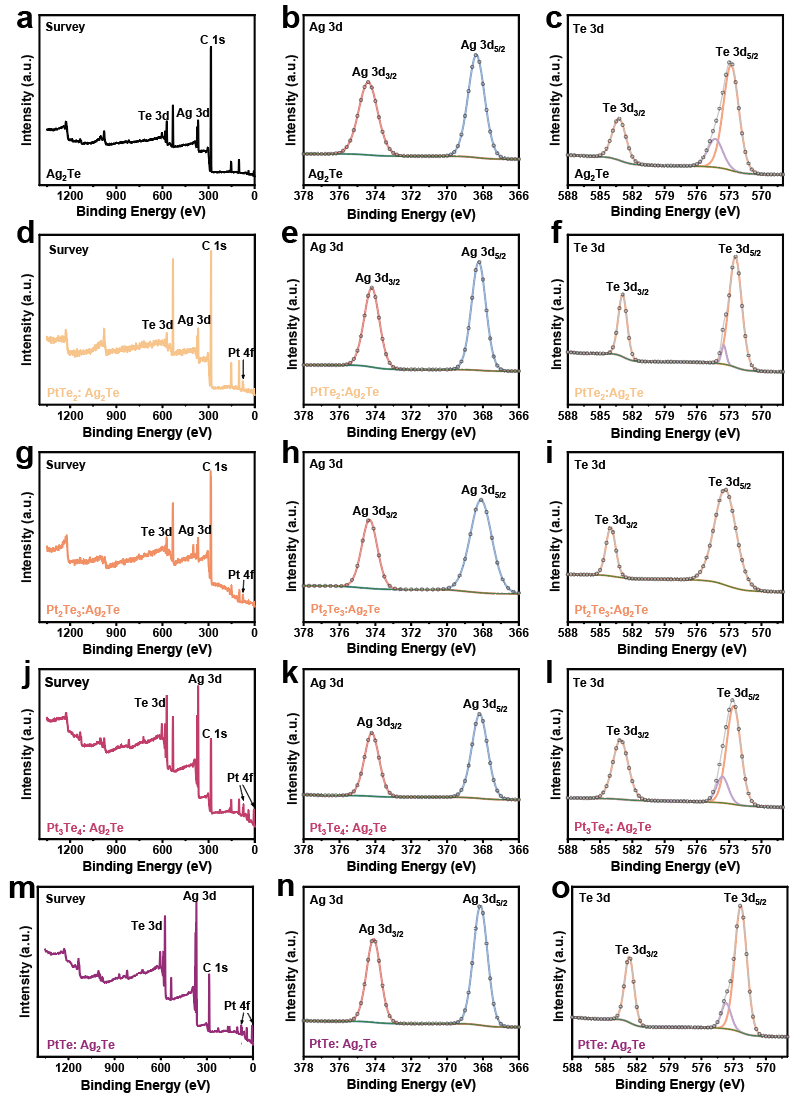


**Supplementary Fig. 3 |** Survey XPS spectra of the a), Ag_2_Te, d) PtTe_2_:Ag_2_Te, g) Pt_2_Te_3_: Ag_2_Te, j) Pt_3_Te_4_: Ag_2_Te, and m) PtTe:Ag_2_Te. High-resolution XPS of Ag 3d region of b) Ag_2_Te, e) PtTe_2_:Ag_2_Te, h) Pt_2_Te_3_: Ag_2_Te, k) Pt_3_Te_4_: Ag_2_Te, and n) PtTe:Ag_2_Te. High-resolution XPS of Te 3d region of c) Ag_2_Te, f) PtTe_2_:Ag_2_Te, i) Pt_2_Te_3_: Ag_2_Te, l) Pt_3_Te_4_: Ag_2_Te, and o) PtTe:Ag_2_Te.


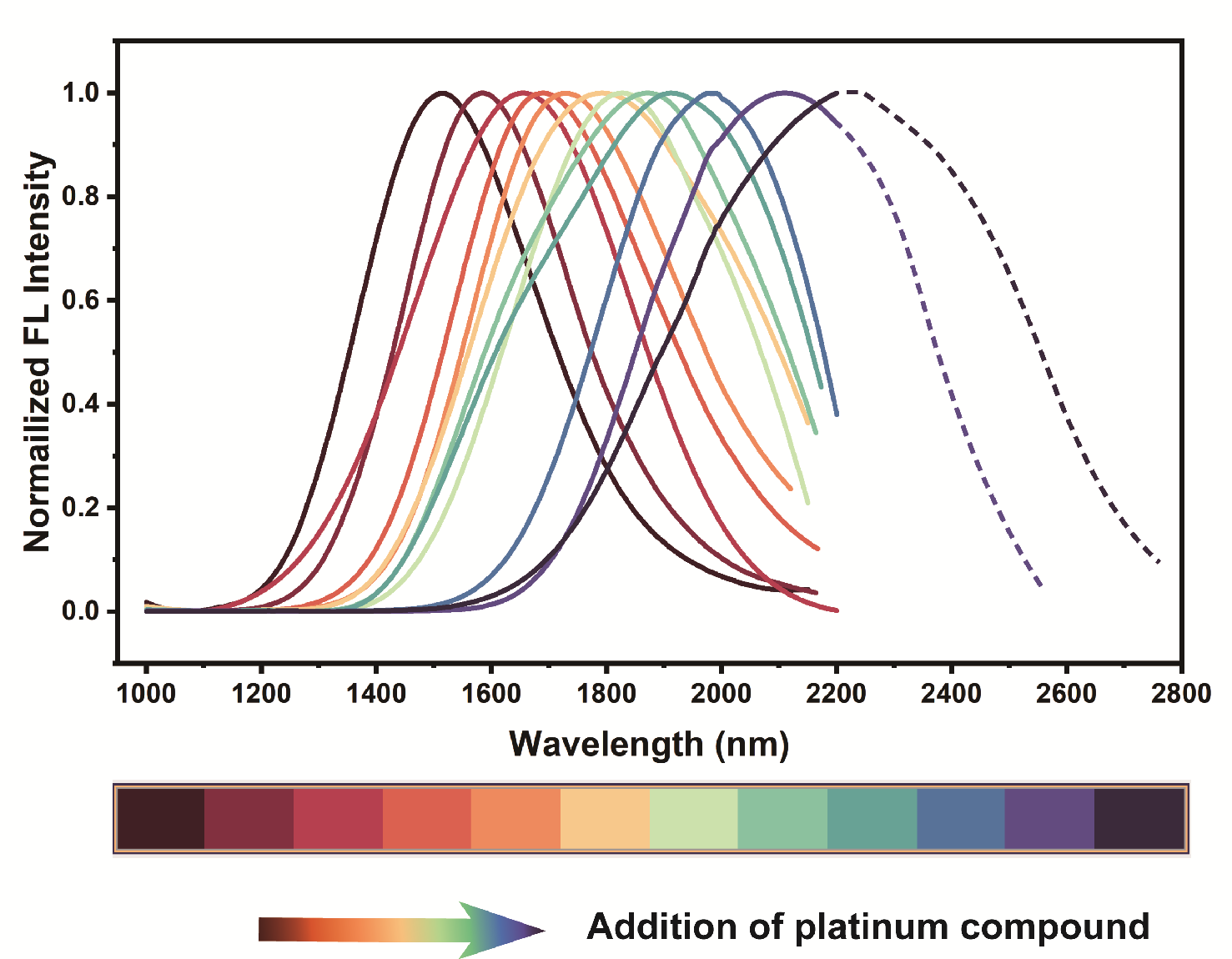


**Supplementary Fig. 4 |** Modulation of the fluorescence emission wavelength of Ag_2_Te and Pt_x_Te_y_:Ag_2_Te QDs under 808 nm excitation. Normalized fluorescence emission spectra of QDs exhibit a progressive redshift with increasing addition of platinum compounds. This indicates the successful modulation of bandgap and emission characteristics via Pt incorporation.


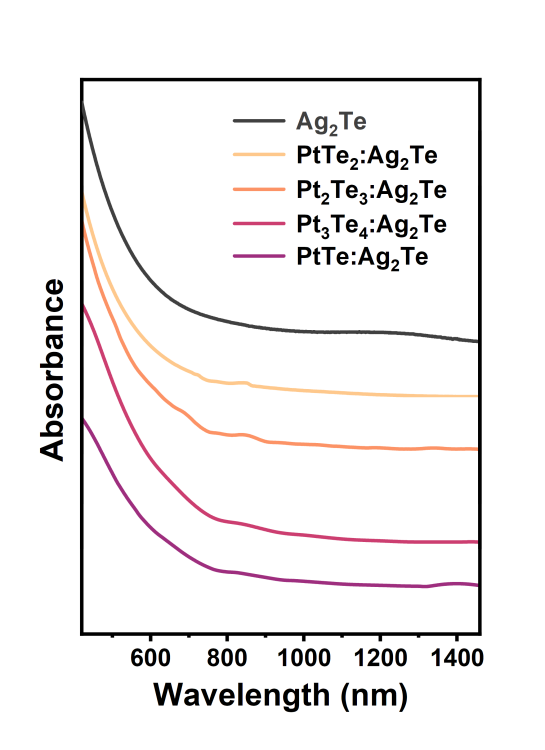


**Supplementary Fig. 5 |** UV-Vis-NIR absorption spectra of Ag_2_Te and Pt_x_Te_y_:Ag_2_Te composite QDs. Absorption spectra of Ag_2_Te, PtTe₂:Ag_2_Te, Pt₂Te₃:Ag₂Te, Pt₃Te₄:Ag₂Te, and PtTe:Ag₂Te QDs. Pt doping accompanied by subtle changes in the absorption profile, suggesting the modified electronic structure and light absorption characteristics of Pt_x_Te_y_:Ag₂Te nanocomposites.


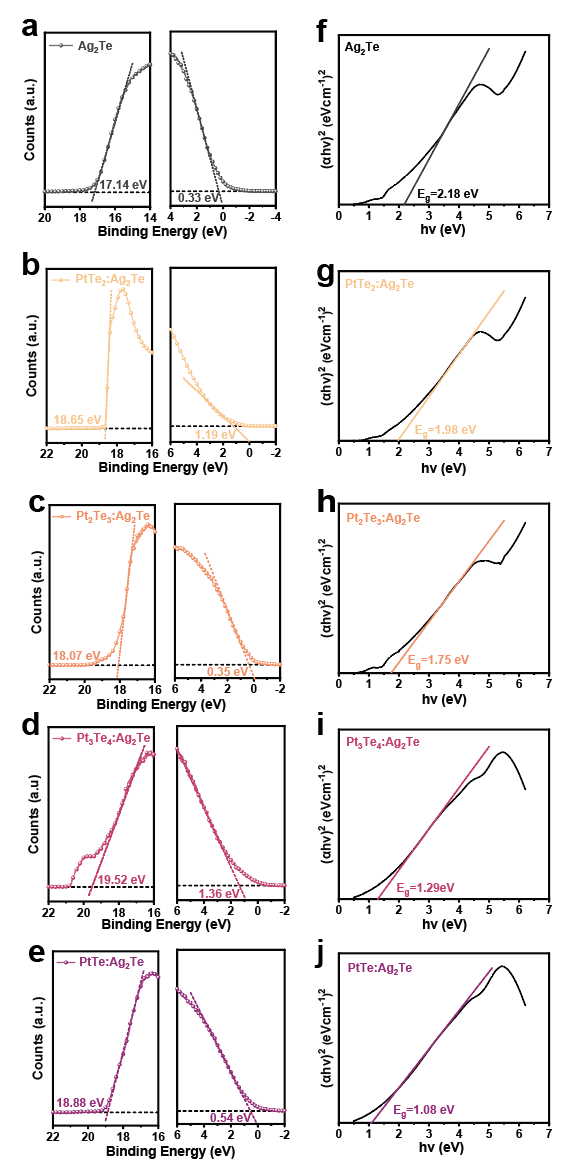


**Supplementary Fig. 6 |** UPS spectra of a) Ag_2_Te QDs, b) PtTe_2_:Ag_2_Te, c) Pt_2_Te_3_:Ag_2_Te, d) Pt_3_Te_4_:Ag_2_Te, e) PtTe:Ag_2_Te. The bandgap (E_g_) of f) Ag_2_Te QDs, g) PtTe_2_:Ag_2_Te, h) Pt_2_Te_3_:Ag_2_Te, i) Pt_3_Te_4_:Ag_2_Te, j) PtTe:Ag_2_Te.


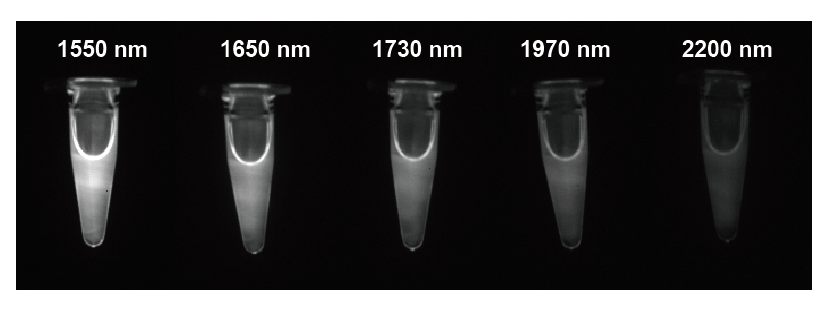


**Supplementary Fig. 7 |** NIR-II fluorescence imaging of Pt_x_Te_y_:Ag₂Te-OIPA QDs with different emission wavelengths. NIR-II fluorescence images of Pt_x_Te_y_:Ag₂Te-OIPA QDs with emission peaks at 1550 nm (Ag₂Te), 1650 nm (PtTe₂:Ag₂Te), 1730 nm (Pt₂Te₃:Ag₂Te), 1970 nm (Pt₃Te₄:Ag₂Te) and 2200 nm (PtTe:Ag₂Te) respectively. All samples were measured at a concentration of 1 mg/mL using an 808 nm excitation laser (power density: 80 mW/cm²) with a 1300 nm LP filter and an exposure time of 50 ms.


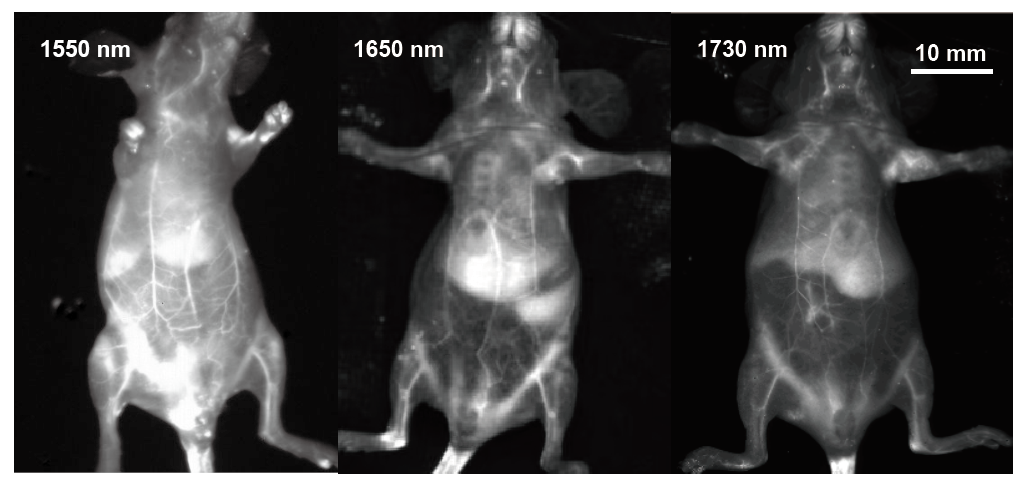


**Supplementary Fig. 8 |** NIR-II in vivo fluorescence imaging of BALB/c nude mice after intravenous injection of Pt_x_Te_y_:Ag₂Te-OIPA QDs with different emission wavelengths. NIR-II fluorescence images of mice acquired after tail vein injection (200 μL, 5 mg/mL) of QDs with emission peaks at 1550, 1650, and 1730 nm. Fluorescence imaging was performed using a NIR-II imaging system (with a 1300 nm LP filter) under 808 nm laser excitation at a power density of 80 mW/cm² with an exposure time of 200 ms. Scale bars: 10 mm.


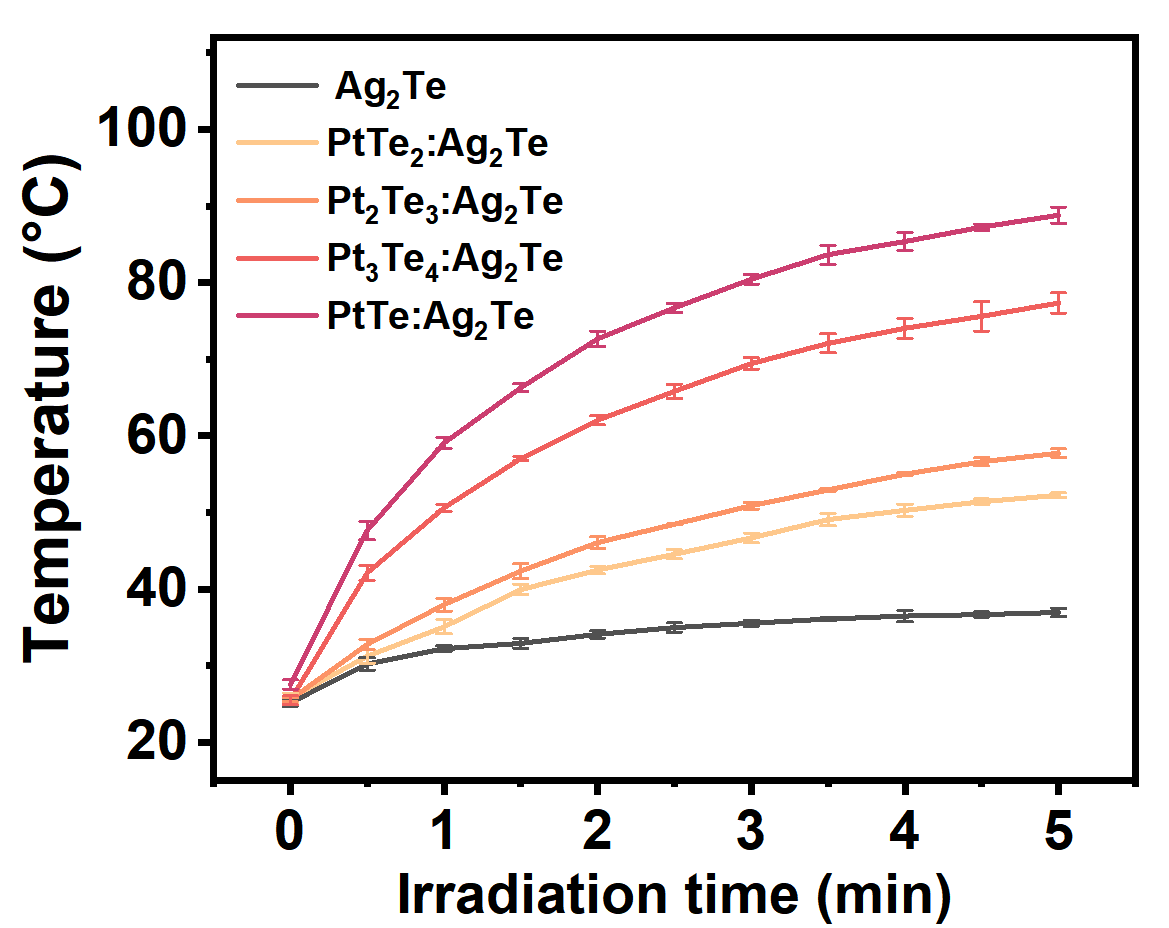


**Supplementary Fig. 9 |** Photothermal heating curves of Pt_x_Te_y_:Ag_2_Te (Ag₂Te, PtTe₂:Ag₂Te, Pt₂Te₃:Ag₂Te, Pt₃Te₄:Ag₂Te and PtTe:Ag₂Te) dispersions at a concentration of 3 mg/ mL under 808 nm laser irradiation (0.28 W/cm², 5 min). Data are presented as mean ± s.d. from three independent samples (n = 3).


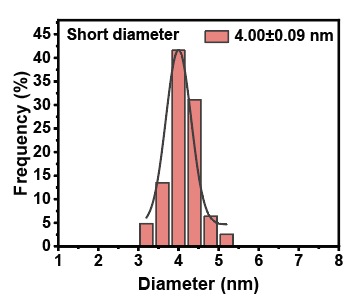


**Supplementary Fig. 10 |** Size distribution histogram of the short diameter of Pt_2_Te_3_:Ag_2_Te QDs measured from the TEM image shown in Fig. 3a. The particle diameters were measured and fitted with a Gaussian distribution, revealing an average size of 4.00 ± 0.09 nm (n≥300).


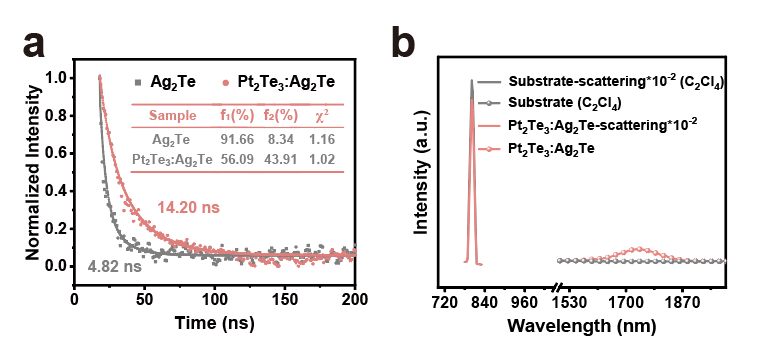


**Supplementary Fig. 11 |** Fluorescence lifetime and quantum yield of Pt_2_Te_3_:Ag_2_Te composite QD. **a**, the transient fluorescence spectra and fluorescence lifetime fitting curves of Ag_2_Te and Pt_2_Te_3_:Ag_2_Te composite QD. **b**, Absolute PLQY (Photoluminescence Quantum Yield) of Pt_2_Te_3_:Ag_2_Te composite QD.


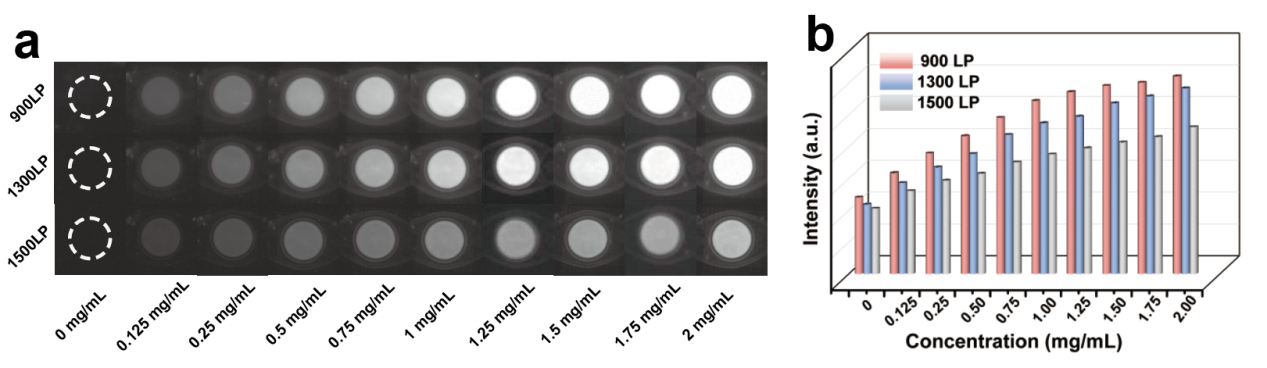


**Supplementary Fig. 12 |** NIR-II fluorescence imaging and intensity analysis of the Pt_2_Te_3_:Ag_2_Te-OIPA QDs at various concentrations under different long-pass (LP) filters. **a**, NIR-II fluorescence images of the Pt_2_Te_3_:Ag_2_Te-OIPA QDs at concentrations ranging from 0–2.0 mg/mL under three LP filters (900 nm, 1300 nm, and 1500 nm). **b**, Corresponding quantitative fluorescence intensity analysis, showing a concentration-dependent enhancement. Imaging was performed under excitation with an 808 nm laser at a power density of 80 mW/cm² and an exposure time of 50 ms for all groups.


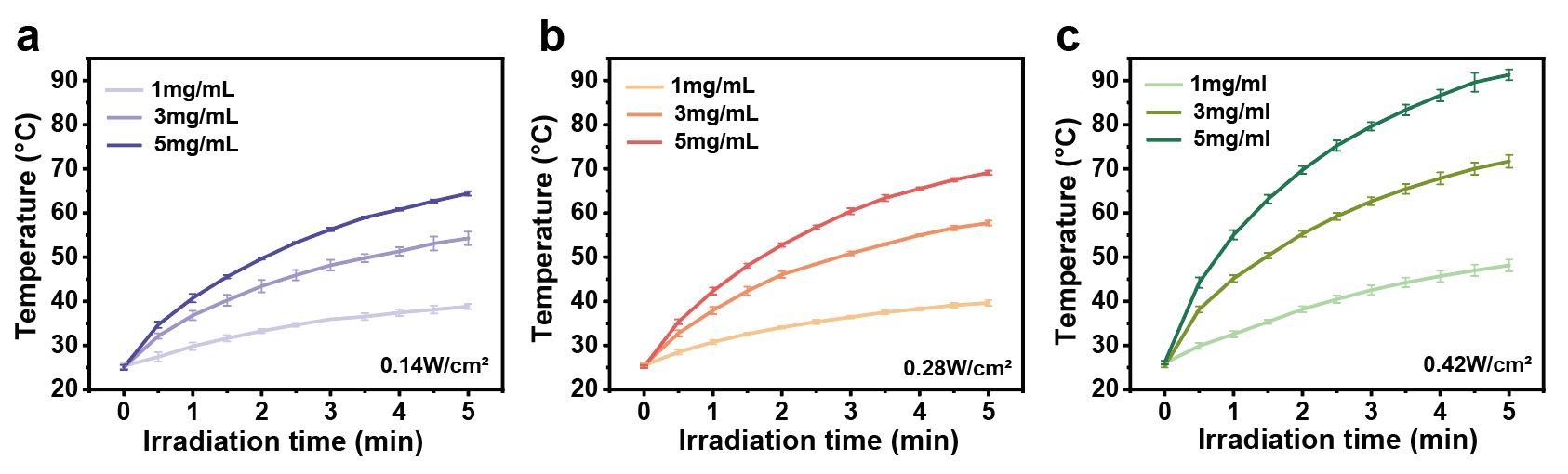


**Supplementary Fig. 13 |** Photothermal heating curves of Pt₂Te₃:Ag₂Te-OIPA QDs dispersions at varying concentrations (1, 3, and 5 mg/mL) under 808 nm laser irradiation at different power densities: **a**, 0.14 W/cm^2^; **b**, 0.28 W/cm²; and **c**, 0.42 W/cm². Data are presented as mean ± s.d. from three independent samples (n = 3).


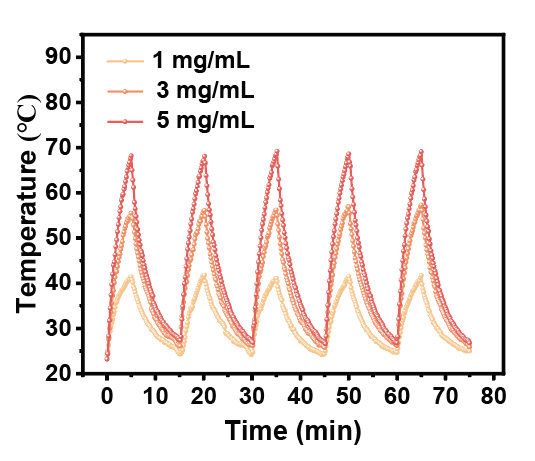


**Supplementary Fig. 14 |** Photothermal stability of Pt_2_Te_3_:Ag_2_Te-OIPA QDs at different concentrations (1, 3, and 5 mg/mL) under five laser on/off cycles. Each cycle consisted of 5 minutes of 808 nm laser irradiation followed by a cooling period. The power density was approximately 0.28 W/cm².


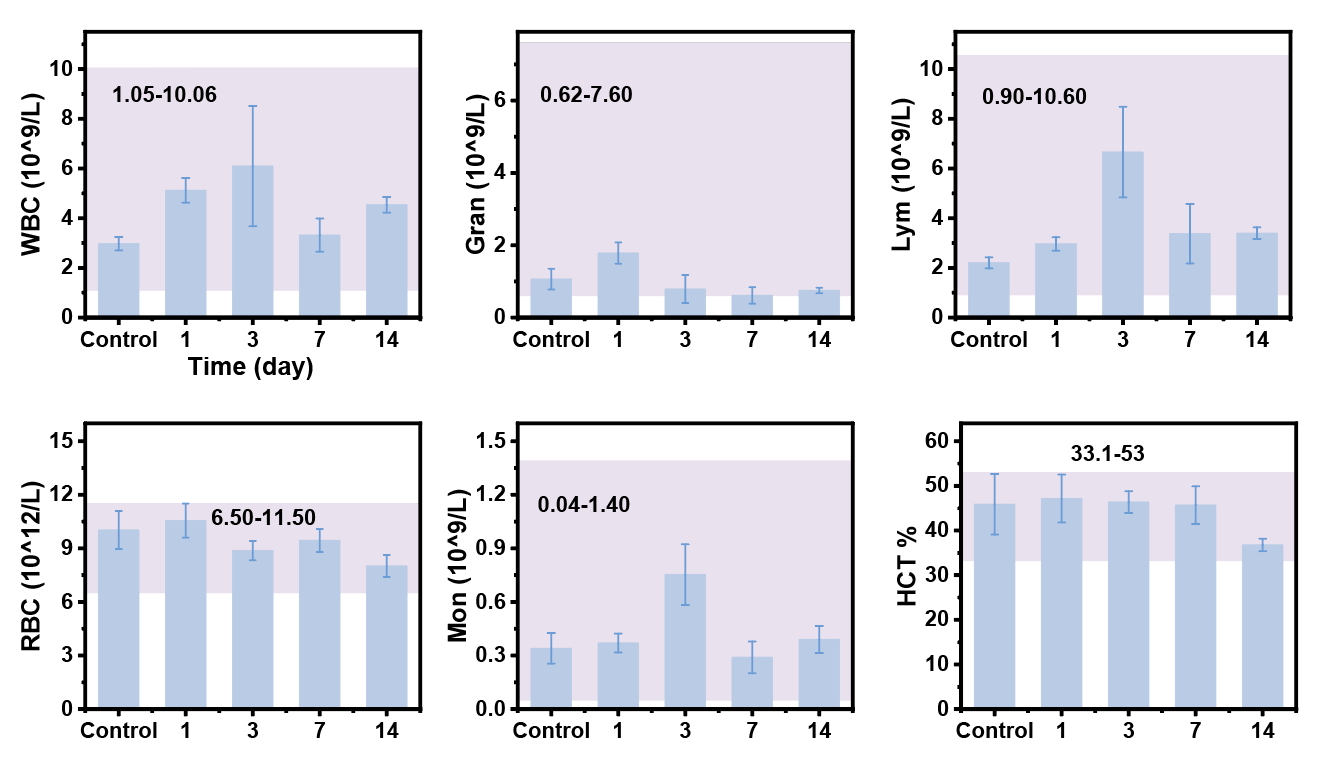


**Supplementary Fig. 15 | Hematological analysis after systemic administration of PAPD. Hematological parameters including (a) white blood cell (WBC), (b) granulocyte (Gran), (c) lymphocyte (Lym), (d) red blood cell (RBC), (e) monocyte (Mon), and (f) hematocrit (HCT) levels were assessed in C57BL/6 mice at day 1, 3, 7, and 14 after a single tail vein injection of 200 μL of Pt₂Te₃:Ag₂Te–OIPA QDs (5 mg/mL). The shaded regions represent the normal physiological ranges for C57BL/6 mice. Error bars represent mean ± s.d. (n = 3 per group).**


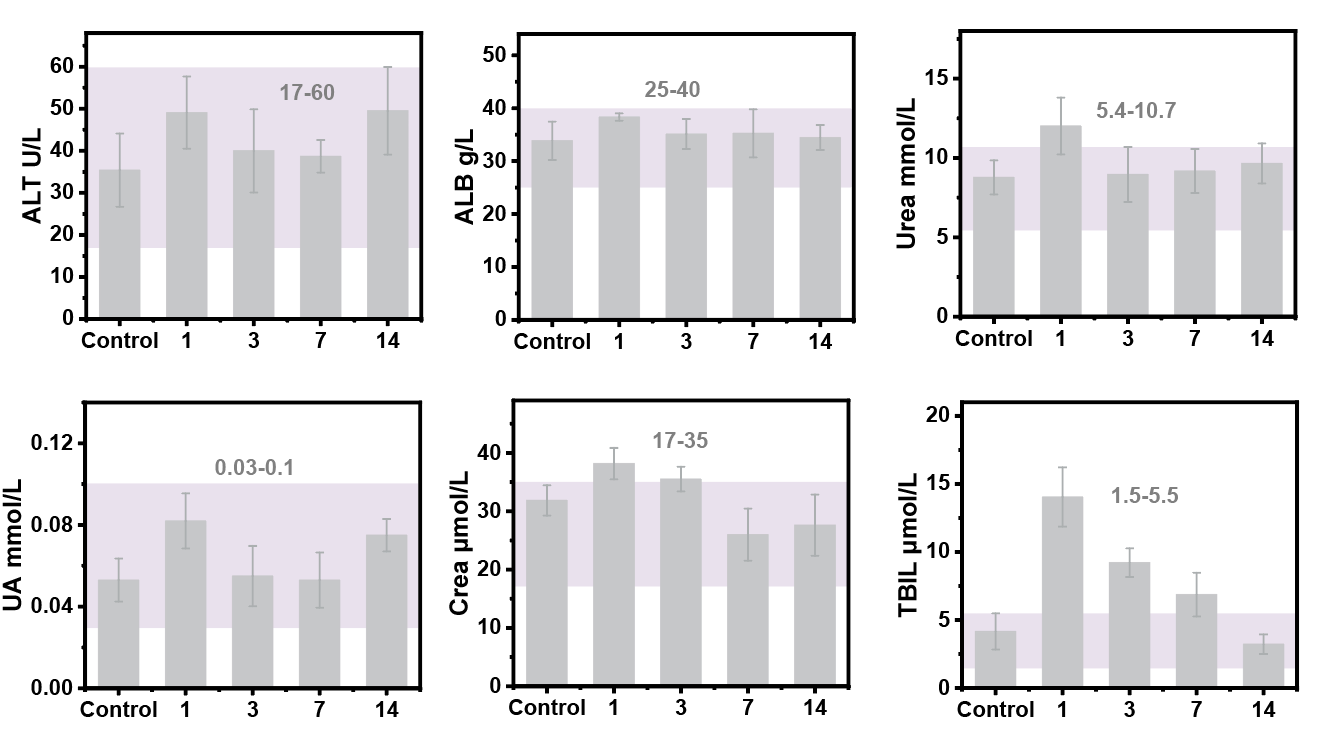


**Supplementary Fig. 16 | Blood biochemistry analysis after systemic administration of** Pt_2_Te_3_:Ag_2_Te-OIPA QDs**. Blood biochemical parameters including (a) alanine aminotransferase (ALT), (b) albumin (ALB), (c) urea, (d)uric acid (UA), (e) creatinine (Crea), and (f) total bilirubin (TBIL) were measured in C57BL/6 mice at day 1, 3, 7, and 14 following a single tail vein injection of 200 μL of** Pt_2_Te_3_:Ag_2_Te-OIPA QDs **(5 mg/mL). The shaded regions represent the normal physiological ranges for C57BL/6 mice. Error bars represent mean ± s.d. (n = 3 per group).**


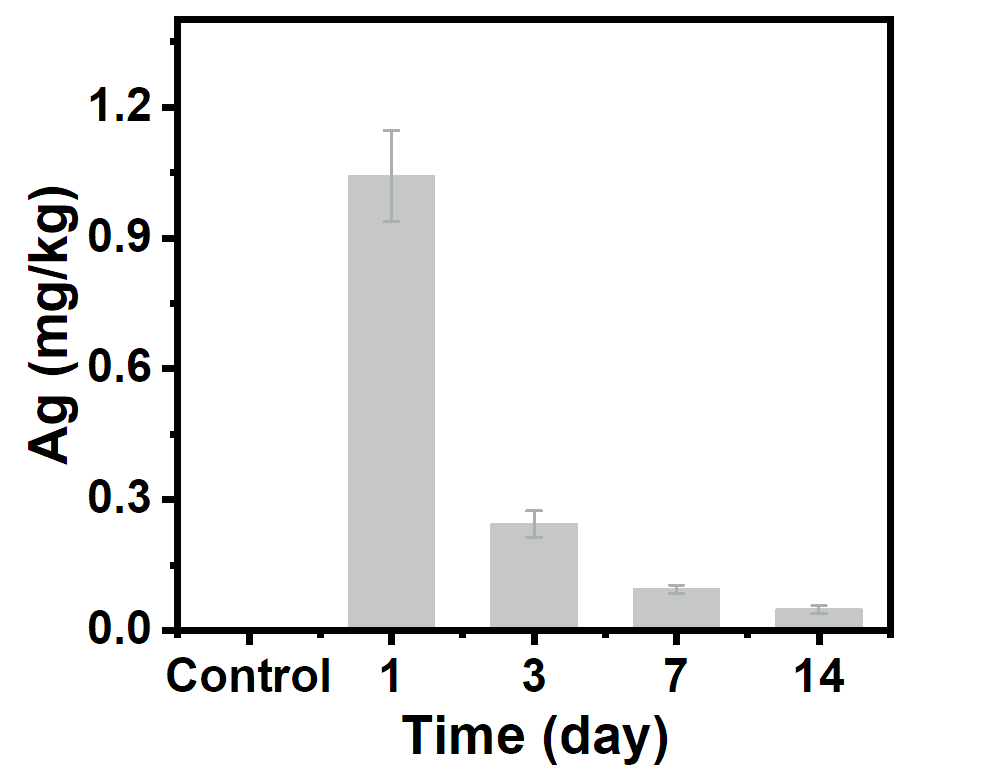


**Supplementary Fig. 17 |** Quantification of silver content in blood over time post-injection. The concentration of Ag in mouse blood was determined by inductively coupled plasma mass spectrometry (ICP-MS) at 1, 3, 7, and 14 days following a single intravenous injection of 200 μL Pt₂Te₃:Ag₂Te-OIPA QDs (5 mg/mL). Error bars represent mean ± s.d. (n = 3 per group).


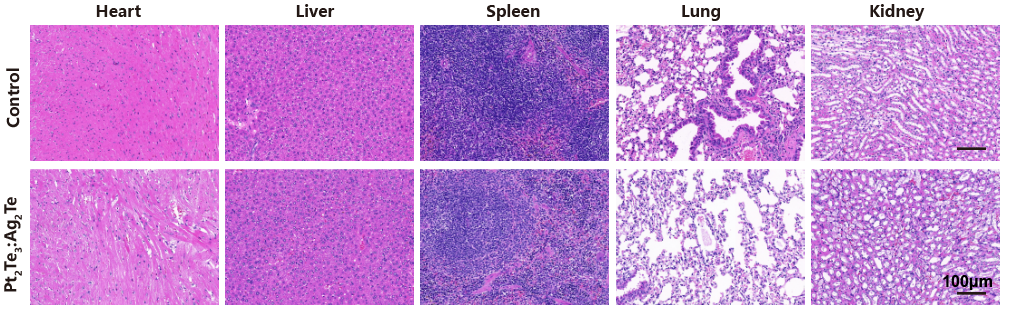


**Supplementary Fig. 18 |** H&E staining of major organs after treatment with Pt₂Te₃:Ag₂Te-OIPA QDs. RepresentativeH&E staining images of major organs including heart, liver, spleen, lung and kidney harvested on day 14 after intravenous injection of 200 μL Pt₂Te₃:Ag₂Te-OIPA QDs (5 mg/mL). No significant pathological changes or tissue damage were observed compared to the control group, indicating favorable biocompatibility. Scale bar, 100 μm.


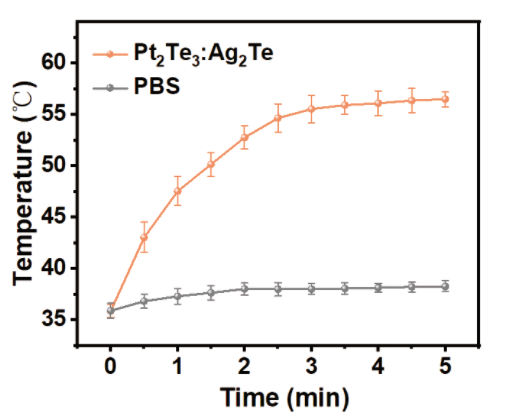


**Supplementary Fig. 19 |** Tumor site temperature profiles following NIR irradiation. BALB/c nude mice bearing subcutaneous tumors were intratumorally injected with 30 μL of Pt₂Te₃:Ag₂Te-OIPA QDs (3 mg/mL) or PBS (control group), followed by irradiation with an 808 nm laser at a power density of 0.28 W/cm² for 5 min. Temperature changes at the tumor sites were recorded using an infrared thermal camera. Error bars represent mean ± s.d. (n = 5 per group).


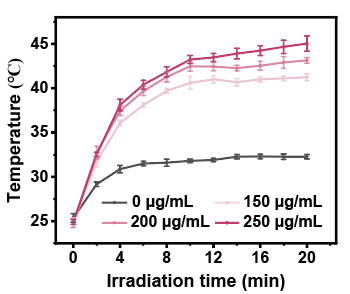


**Supplementary Fig. 20 |** Photothermal heating curves of Pt₂Te₃:Ag₂Te-OIPA QDs at varying concentrations under 808 nm laser irradiation. Temperature profiles of QD solutions (0, 150, 200, and 250 μg/mL) under continuous 808 nm laser exposure (0.72 W/cm², 20 min). Temperature changes at the tumor sites were recorded using an infrared thermal camera. Error bars represent mean ± s.d. (n = 3 per group).


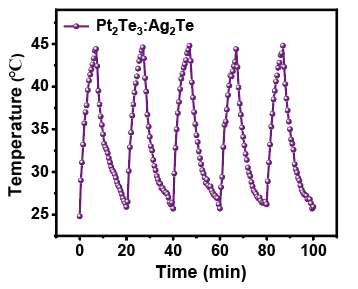


**Supplementary Fig. 21 | Photothermal cycling stability of Pt₂Te₃:Ag₂Te QDs under repeated laser on/off irradiation. Temperature variation of Pt₂Te₃:Ag₂Te-OIPA quantum dot solution (250 μg/mL, 0.2 mL) under five cycles of 808 nm laser irradiation (0.72 W/cm²) with alternating laser on/off intervals.**


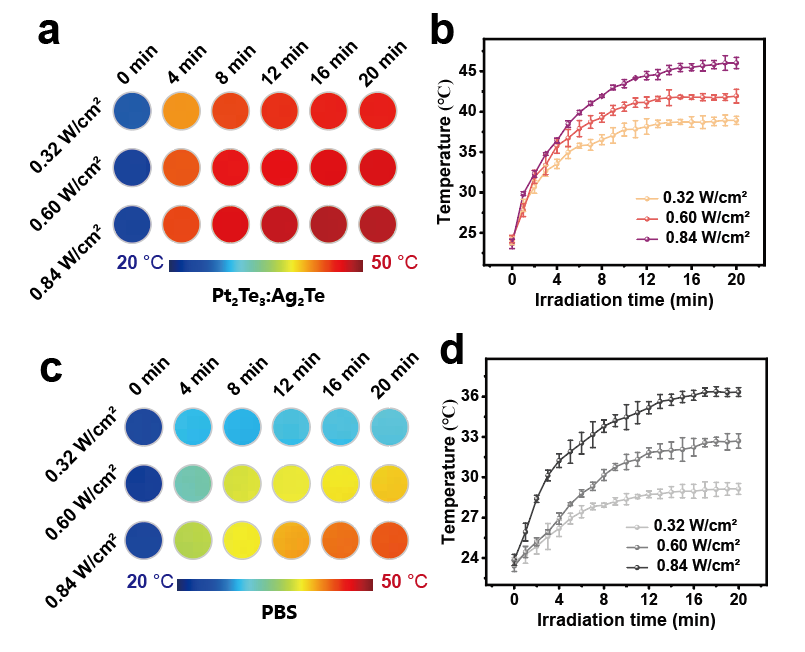


**Supplementary Fig. 22 |** Photothermal behavior of Pt₂Te₃:Ag₂Te-OIPA QDs and PBS under different laser power densities. **a**, Representative thermal images of Pt₂Te₃:Ag₂Te-OIPA QDs (250 μg/mL, 1 mL) irradiated by an 808 nm laser at different power densities (0.32, 0.60, and 0.84 W/cm²) for 0–20 min. **b**, Corresponding quantitative temperature profiles of samples. **c**, Thermal images of PBS under the same irradiation conditions as (a). **d**, Temperature profiles of PBS. Error bars represent mean ± s.d. (n = 3).


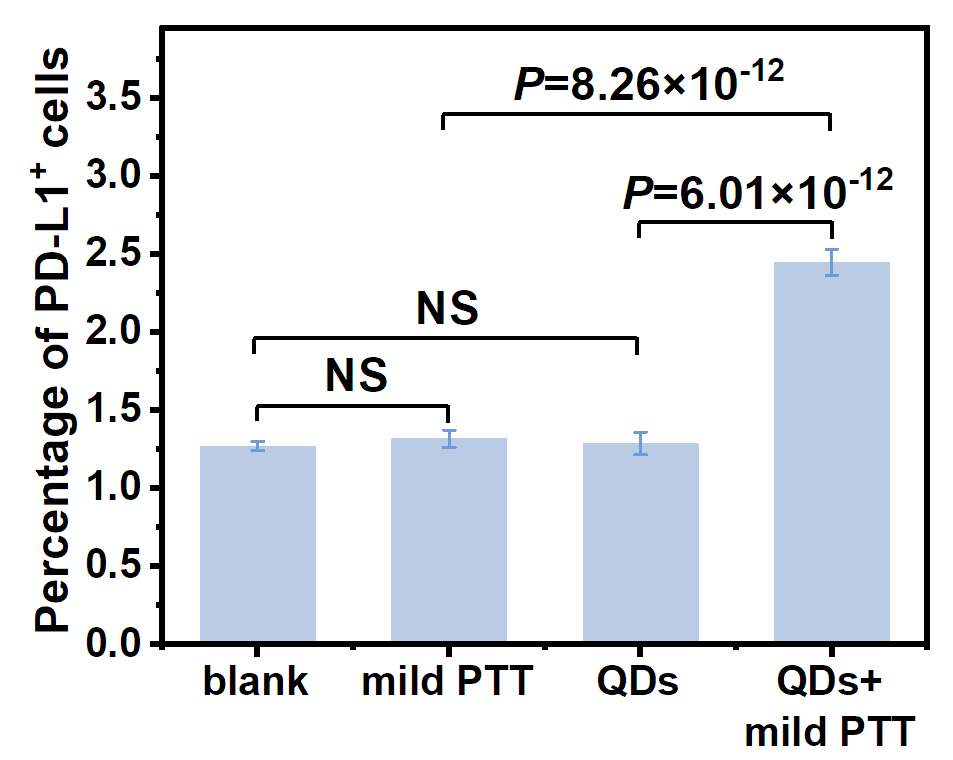


**Supplementary Fig. 23 |** Western blot quantification of PD-L1 protein expression. Band intensities were normalized to the loading control and summarized as mean ± s.d. (n = 4 per group). Statistical significance was determined using one-way ANOVA, which revealed a significant overall difference among groups (F (3, 12) = 338.8, P = 7.36 × 10⁻¹²). Post hoc analysis was performed using Tukey’s multiple comparison test, with no significant differences (NS, P＞0.05) among the blank, mild PTT and QDs groups, while the QDs + mild PTT group exhibited a significant increase compared with the mild PTT group (P =8.26 × 10⁻¹²) and the QDs group (P = 6.01 × 10⁻¹²).

**Supplementary Fig. 24 |** Flow cytometry analysis of fluorescence intensity in tumor cells across four groups. Data are presented as mean ± s.d. (n = 5 per group). One-way ANOVA revealed an extremely significant overall difference among groups (F (3,16) = 10138.04, P = 1.95 × 10⁻²⁶). Post hoc analysis using Tukey’s multiple comparison test showed no significant differences (NS, P＞0.05) among the blank, mild PTT and QDs groups, while the QDs+mild PTT group exhibited a extremely significant increase compared with mild PTT group (P = 2.13 × 10⁻^25^) and QDs group (P = 2.9 × 10⁻^25^).


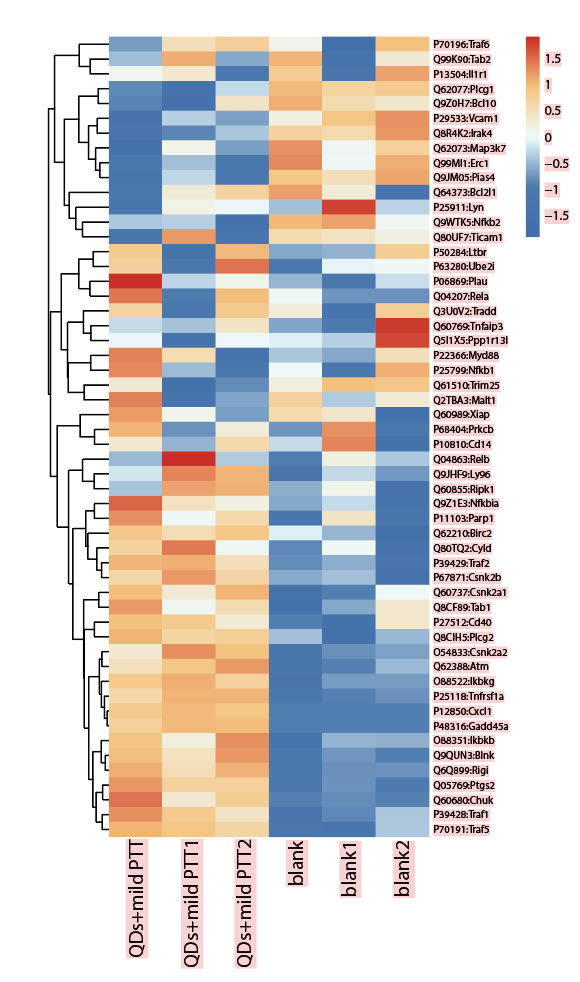


**Supplementary Fig. 25 | Heatmap of immune- and inflammation-related proteins following mild photothermal stimulation.** Heatmap showing the relative expression levels of selected proteins associated with immune signaling and inflammatory pathways in MOC-1 cells after mild photothermal treatment induced by Pt₂Te₃:Ag₂Te-OIPA quantum dots. Cells treated with QDs under mild photothermal conditions (QDs + mild PTT, n = 3) were compared with untreated control cells (blank, n = 3). Protein abundances were Z-score normalized across samples and hierarchically clustered based on Euclidean distance. The color scale represents relative expression levels, with red indicating higher and blue indicating lower abundance.


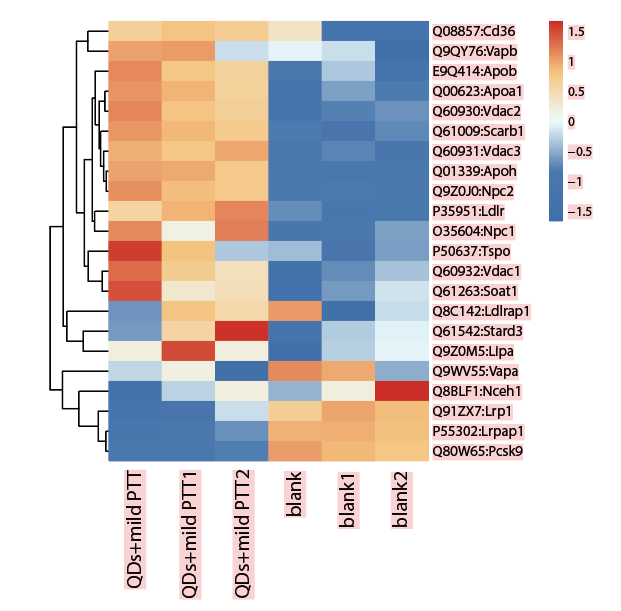


**Supplementary Fig. 26 | Heatmap of lipid metabolism and cholesterol transport-associated proteins following mild photothermal treatment.** Heatmap showing the relative expression profiles of selected proteins involved in lipid metabolism, cholesterol transport, and membrane trafficking in MOC-1 cells after mild photothermal stimulation induced by Pt₂Te₃:Ag₂Te-OIPA quantum dots. Cells treated with QDs under mild photothermal conditions (QDs + mild PTT, n = 3) were compared with untreated control cells (blank, n = 3). Protein abundances were Z-score normalized across samples and hierarchically clustered based on Euclidean distance. The color scale indicates relative expression levels, with red and blue representing higher and lower abundance, respectively.


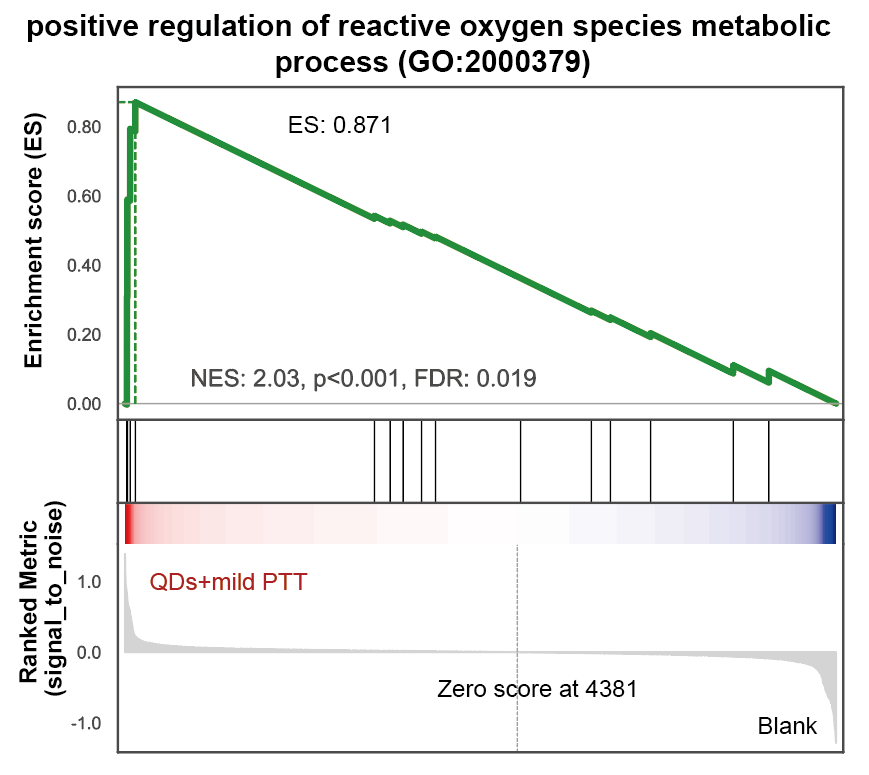


**Supplementary Fig. 27 |** Gene set enrichment analysis (GSEA) of ROS-related pathways following mild photothermal treatment. GSEA showing significant enrichment of the Gene Ontology term positive regulation of reactive oxygen species metabolic process (GO:2000379) in MOC-1 cells after mild photothermal treatment induced by Pt₂Te₃:Ag₂Te-OIPA quantum dots. The enrichment plot indicates coordinated upregulation of ROS-associated proteins in the treated group compared with untreated controls (QDs+mild PTT vs. Blank). Enrichment score (ES), normalized enrichment score (NES), nominal p value, and false discovery rate (FDR) are indicated.


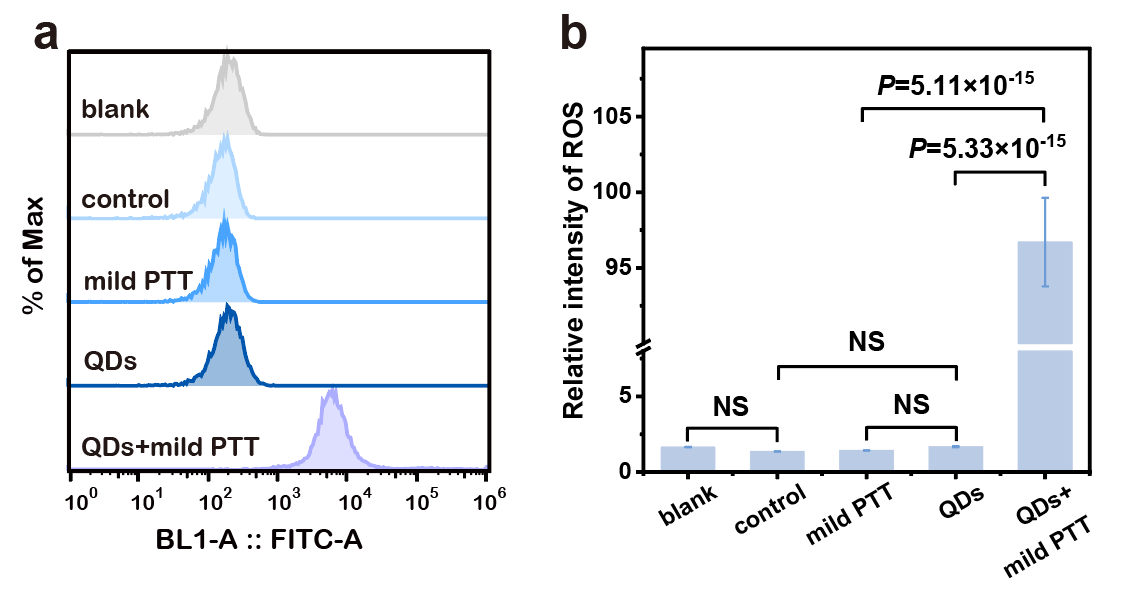


**Supplementary Fig. 28 | Flow cytometry analysis of intracellular ROS generation under different treatment conditions. a, representative flow cytometry histograms of ROS levels in cells treated with blank, control, mild photothermal treatment (mild PTT), QDs, and QDs + mild PTT. b, Quantitative analysis of ROS fluorescence intensity. Data are presented as mean ± s.d. (n = 3 per group). A broken y-axis is applied to visualize the large difference between groups. One-way ANOVA revealed an extremely significant overall difference among groups (F (4,10) = 3159.65, P = 1.85 × 10⁻¹⁵). Post hoc Tukey’s multiple comparison test showed no significant differences (NS, P＞ 0.05) among the blank, control, mild PTT and QDs groups, whereas the QDs + mild PTT group exhibited extremely significant increases compared with mild PTT group (P = 5.11 × 10⁻¹⁵) and QDs group (P = 5.33 × 10⁻¹⁵).**


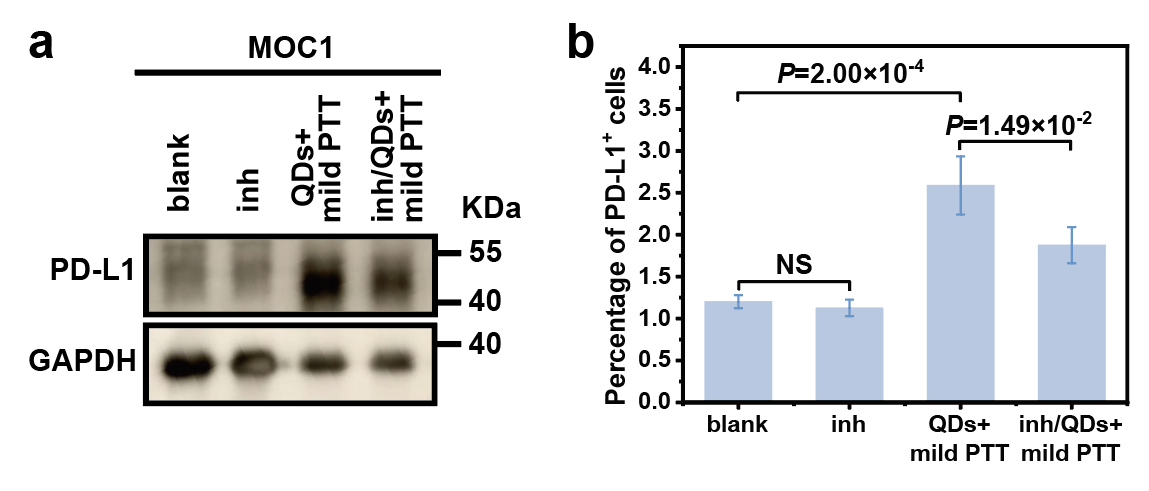


**Supplementary Fig. 29 |** **Western blot analysis of PD-L1 expression in MOC-1 cells with mild photothermal treatment and NF-κB inhibition. a, Western blot analysis of PD-L1 protein expression in MOC-1 cells following different treatments: untreated control (blank), NF-κB inhibitor (inh), Pt₂Te₃:Ag₂Te quantum dots combined with mild photothermal treatment (QDs + mild PTT), and inhibitor combined with QDs and mild photothermal treatment (inh/QDs + mild PTT). GAPDH was used as a loading control. Protein lysates were collected 48 h after treatment initiation. Mild photothermal stimulation was applied twice (at 0 h and 24 h) to evaluate its effect on PD-L1 expression. b, Quantification of PD-L1 expression based on band intensities normalized to GAPDH. Data are presented as mean ± s.d. (n = 3 per group). One-way ANOVA revealed a significant overall difference among groups (F (3, 8) = 30.52, P = 9.94 × 10⁻^5^). Tukey’s multiple comparisons test showed that PD-L1 expression was significantly increased in the QDs + mild PTT group compared with the inh/QDs + mild PTT group (P = 1.49 × 10⁻²) and blank group (P = 2.00 × 10⁻^4^). No significant difference was observed between the blank and inhibitor groups (NS, P＞ 0.05).**


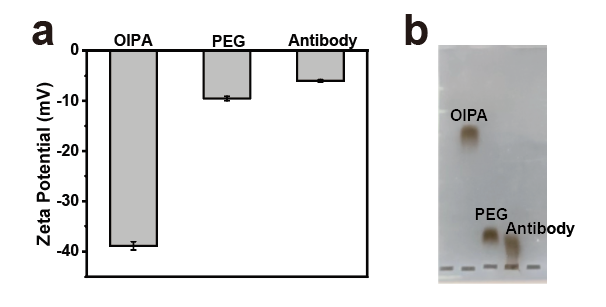


**Supplementary Fig. 30 |** Surface modification of Pt₂Te₃:Ag₂Te quantum dots during platform construction. **a**, Zeta potential variation of Pt₂Te₃:Ag₂Te QDs during stepwise surface modification. Error bars represent mean ± s.d. (n = 3 per group). **b**, Agarose gel electrophoresis validated the successful surface functionalization, showing decreased mobility with increasing surface neutrality from OIPA to PEG and antibody-modified QDs.


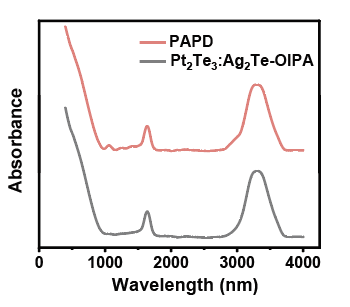


**Supplementary Fig. 31 |** FTIR spectra of Pt₂Te₃:Ag₂Te-OIPA QDs and PAPD platform. The emergence of new bands corresponds to the functional groups introduced by antibody conjugation and PEGylation during PAPD construction.


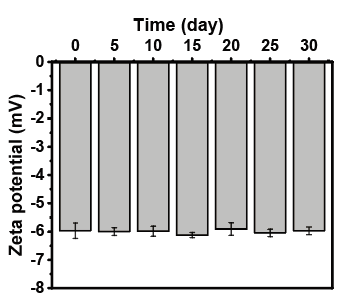


**Supplementary Fig. 32 |** Zeta potential stability of PAPD over time. Zeta potential values of the PAPD measured over a 30-day period, showing minimal variation and indicating good colloidal stability in aqueous solution. Error bars represent mean ± s.d. (n = 3).


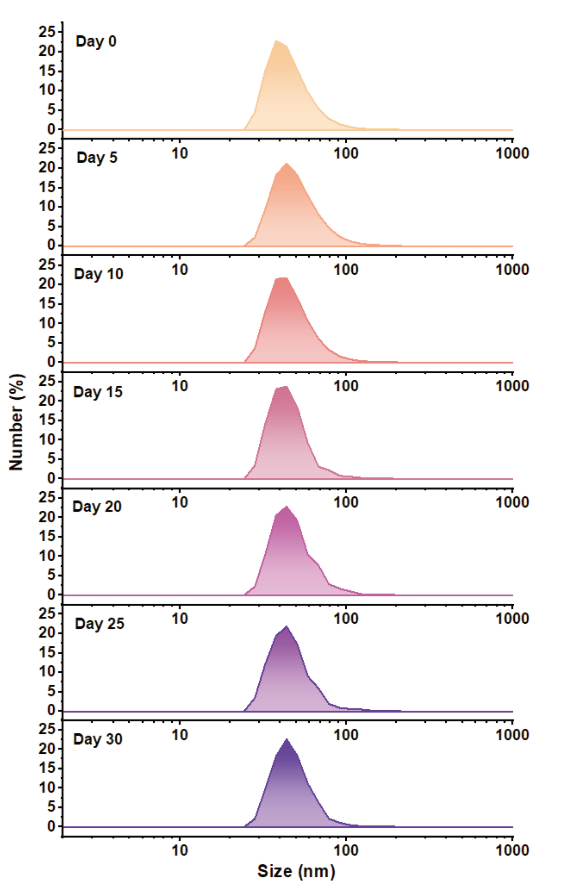


**Supplementary Fig. 33 |** Stability evaluation of PAPD based on hydrodynamic size over time. Dynamic light scattering (DLS) analysis was performed to monitor the particle size distribution of PAPD stored in PBS at 4℃ for 30 days. Measurements were taken at 5-day intervals.


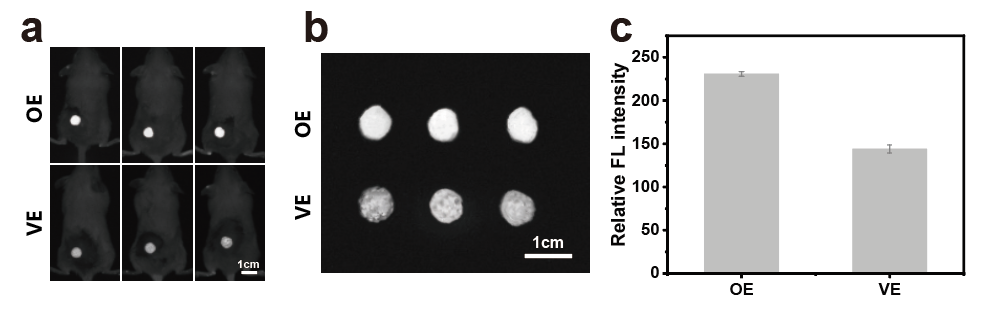


**Supplementary Fig. 34 |** In vivo and ex vivo fluorescence imaging to evaluate the tumor-targeting capability of PAPD in C57BL/6 mice bearing tumors. **a**, In vivo NIR-II fluorescence images of tumor-bearing mice (OE: **overexpressing**; VE: **vector-expressing**) at 4 h post-injection of PAPD (200 μL, 5 mg/mL) via tail vein. Imaging conditions: 808 nm laser excitation, 1300 nm LP filter, exposure time 200 ms, power density 80 mW/cm². **b**, Ex vivo fluorescence images of excised tumors from OE and VE groups (scale bar: 1 cm). **c**, Quantitative analysis of relative fluorescence intensity of tumors from OE and VE groups from (a). Data are presented as mean ± s.d. (n = 3).


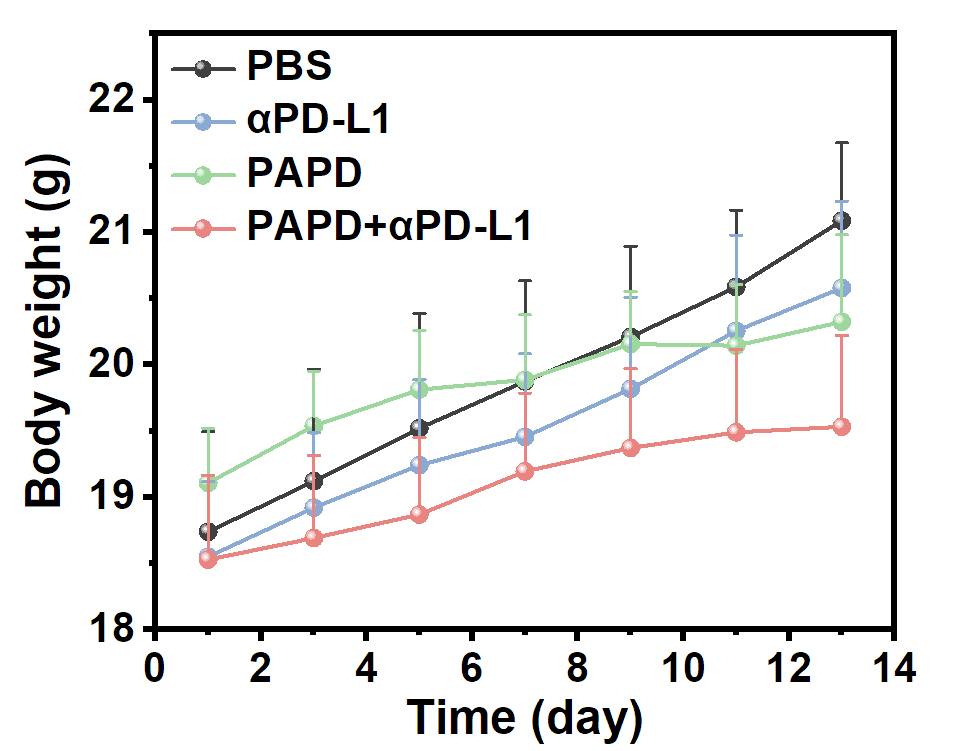


**Supplementary Fig. 35 |** Body weight variation in mice during different treatments. Body weights of tumor-bearing mice were recorded every two days over a 14-day treatment period. Error bars represent mean ± s.d. (n = 5 per group).


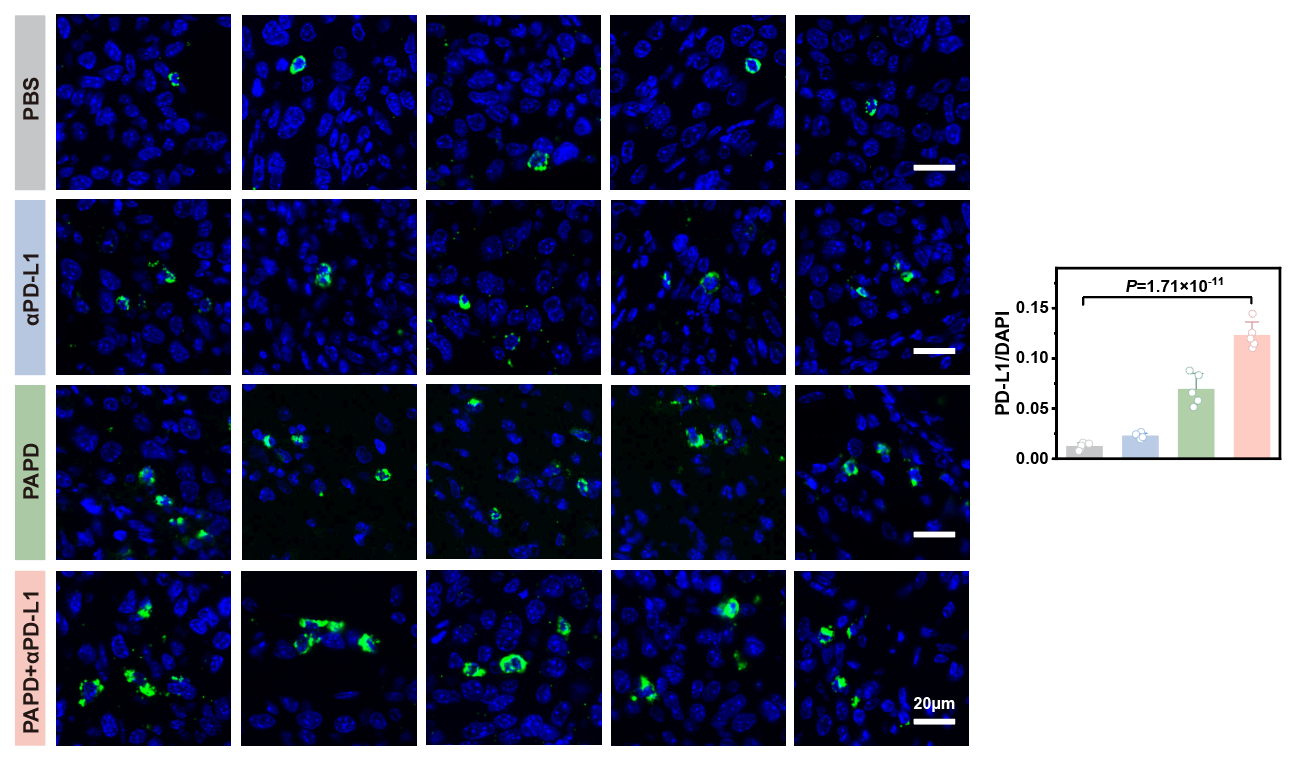


**Supplementary Fig. 36 |** Immunofluorescence staining of PD-L1 expression in tumor tissues after different treatments. Representative immunofluorescence images (green: PD-L1, blue: DAPI) of tumor slices from mice after treatment with PBS, αPD-L1, PAPD and PAPD + αPD-L1 combination. Scale bar = 20 μm. The right panel shows quantitative analysis of PD-L1/DAPI-positive cell ratios across different treatment groups based on five randomly selected fields (mean ± s.d., n = 5 per group). Statistical significance was determined using one-way ANOVA, which revealed a significant overall difference among groups (F (3, 16) = 114.7, P = 4.97 × 10⁻¹¹). Post hoc analysis was performed using Tukey’s multiple comparison test, with the most significant difference observed between PBS and PB+KT (P = 1.71 × 10⁻¹¹).


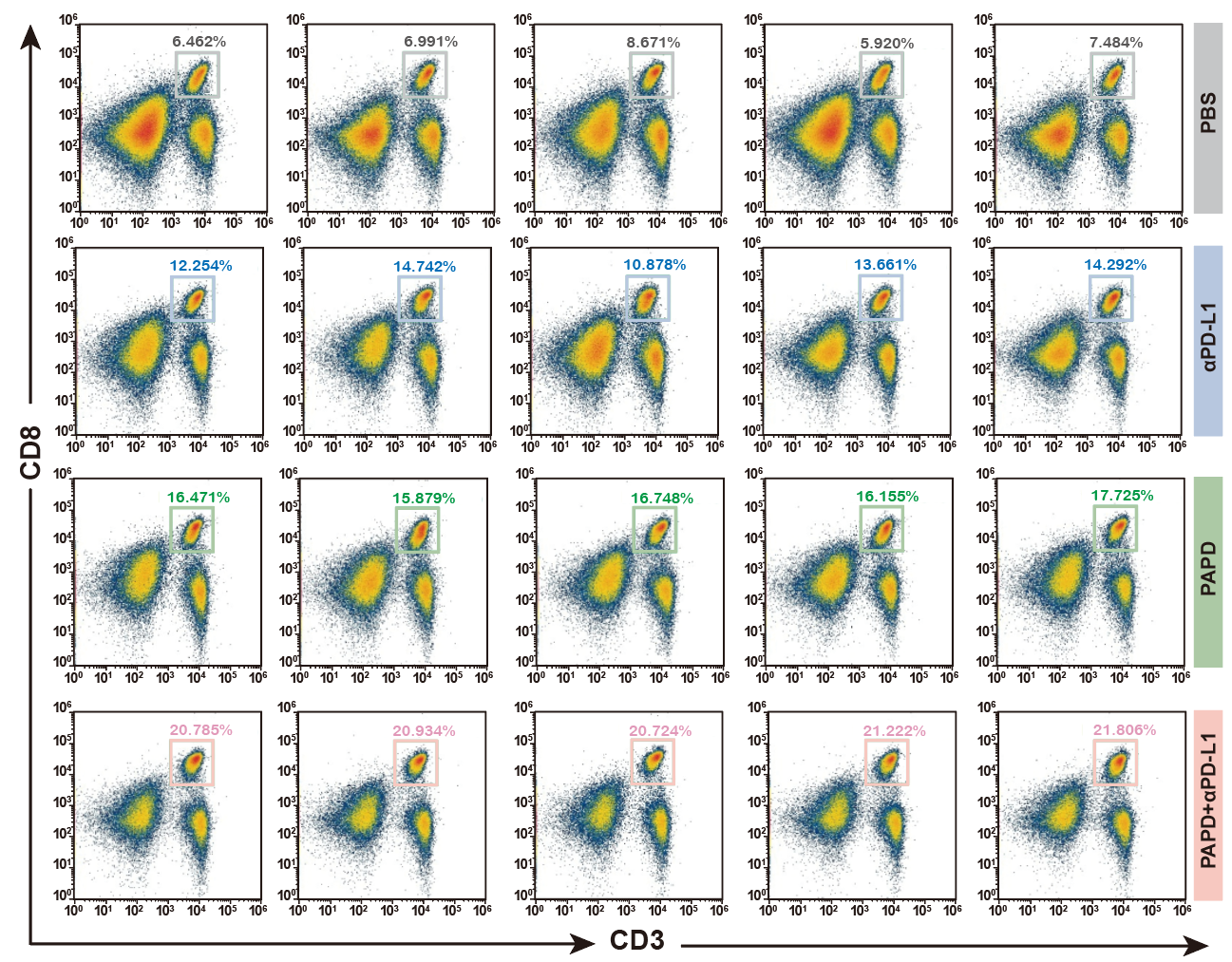


**Supplementary Fig. 37 | Flow cytometry analysis of tumor-infiltrating CD8⁺ T cells from different treatment groups.** Flow cytometry analysis of CD8⁺ T cell infiltration in draining lymph nodes harvested from each treatment group (PBS, αPD-L1, PAPD and PAPD + αPD-L1). Each row shows representative flow cytometry plots for five individual mice per group **(n = 5 per group)**. The percentage in each plot indicates the proportion of CD8⁺ T cells within the total CD3⁺ T cell population. These full datasets are provided here as supplementary material, the leftmost plots from each group have already been shown in Fig. 6h of the main text.


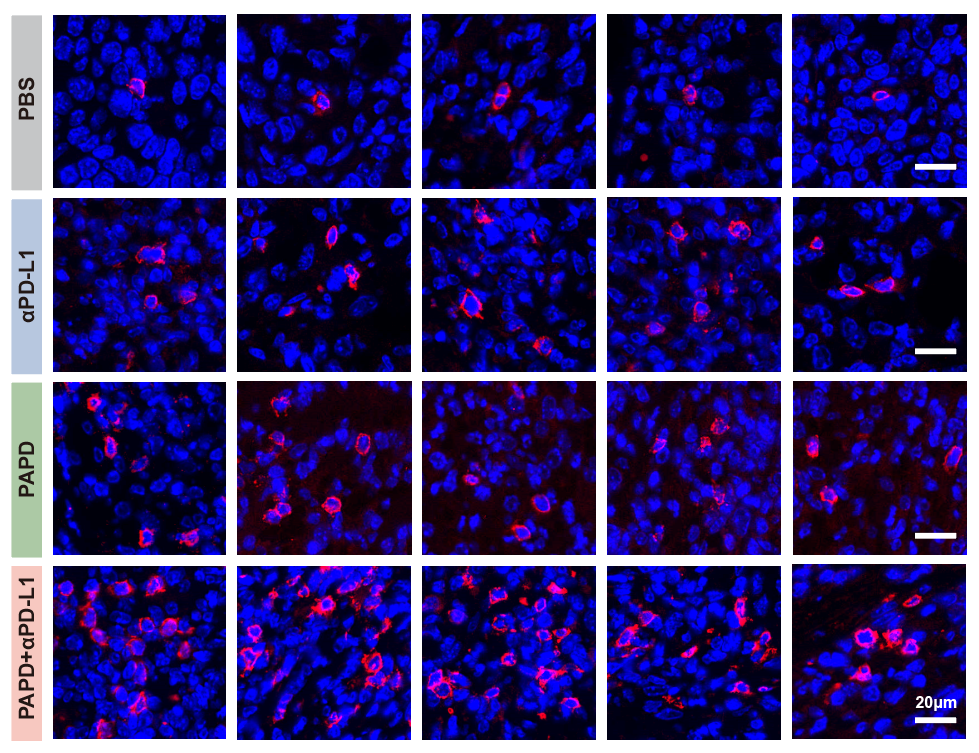


**Supplementary Fig. 38 | Representative immunofluorescence staining of CD8⁺ T cells in tumor tissues from different treatment groups. Tumor tissues collected after treatment with PBS,** α**PD-L1, PAPD and PAPD+**α**PD-L1 were subjected to immunofluorescence staining for CD8⁺ (red) and nuclei (DAPI, blue). Five representative fields were imaged for each group (n = 5 per group). Scale bars: 20 μm. The leftmost image in each group has been shown in the main manuscript as Fig. 6i, and the remaining images are provided here as supplementary data.**


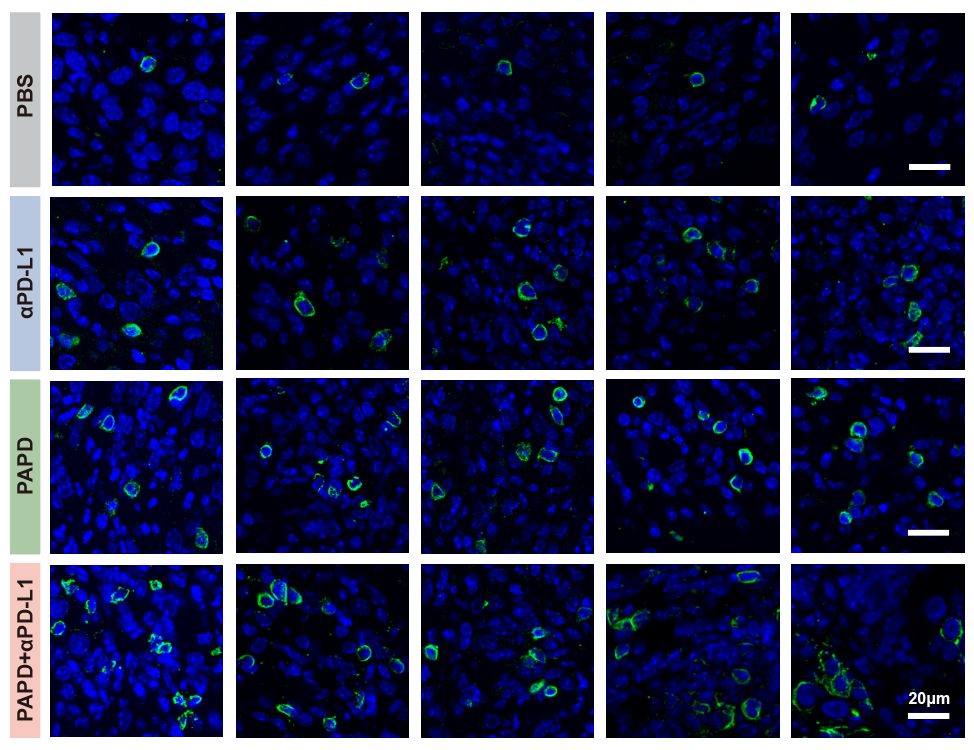


**Supplementary Fig. 39 | Representative immunofluorescence staining of NK1.1⁺ natural killer (NK) cells in tumor tissues from different treatment groups. Tumor tissues from mice treated with PBS,** α**PD-L1, PAPD, or PAPD+αPD-L1 were collected and subjected to immunofluorescence staining for NK1.1⁺ (green) and nuclei (DAPI, blue). Five representative fields were imaged for each group to demonstrate field-to-field consistency. Scale bars: 20 μm. The leftmost image from each group is included in the main manuscript as Fig. 6j, while the remaining fields are shown here in the supplementary data to provide a more complete overview of intratumoral NK1.1⁺ cell distribution and infiltration.**


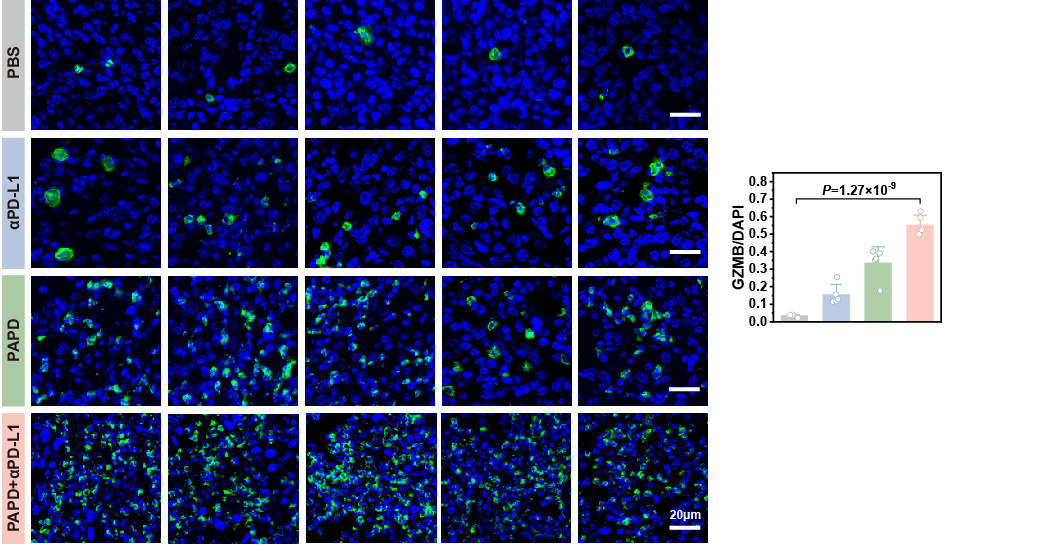


**Supplementary Fig. 40 | Immunofluorescence analysis of GZMB expression in tumor tissues after different treatments. Representative immunofluorescence images (green: GZMB, blue: DAPI) of tumor sections from mice treated with PBS, αPD-L1, PAPD, and PAPD + αPD-L1 combination. Scale bar = 20 μm. The right panel shows quantitative analysis of GZMB/DAPI-positive cell ratios across different treatment groups based on five randomly selected fields (mean ± s.d., n = 5 per group). One-way ANOVA revealed a significant overall difference among groups (F (3,16) = 68.26, P = 2.46 × 10⁻⁹). Tukey’s multiple comparisons test showed a highly significant difference between the PBS and PAPD + αPD-L1 groups (P = 1.27 × 10⁻⁹).**

**
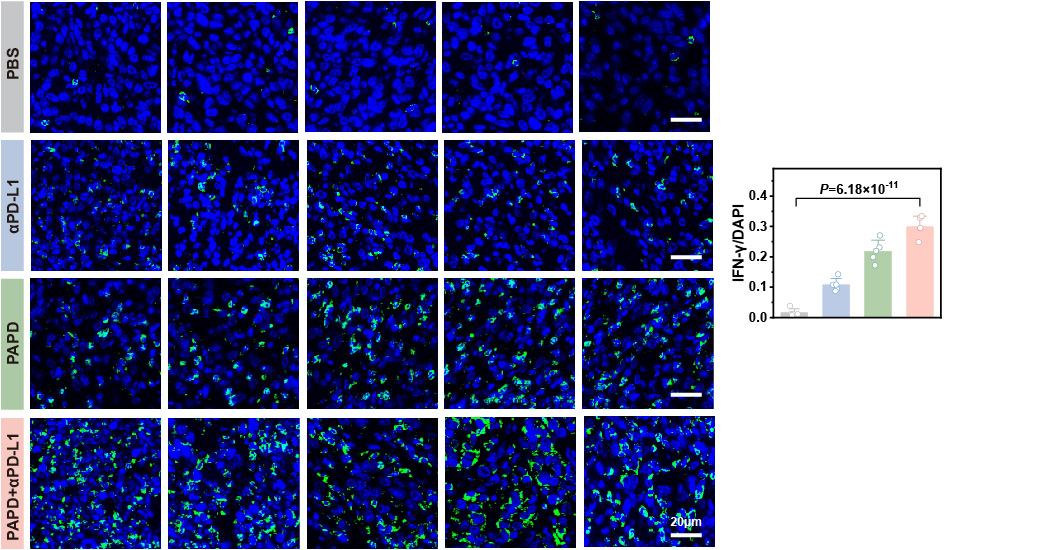
**

**Supplementary Fig. 41 | Immunofluorescence analysis of IFN-γ expression in tumor tissues after different treatments. Representative immunofluorescence images (green: IFN-γ, blue: DAPI) of tumor sections from mice treated with PBS, αPD-L1, PAPD, and PAPD + αPD-L1 combination. Scale bar = 20 μm. The right panel shows quantitative analysis of IFN-γ/DAPI-positive cell ratios across different treatment groups based on five randomly selected fields (mean ± s.d., n = 5 per group). One-way ANOVA revealed a significant overall difference among groups (F (3,16) = 98.88, P = 1.53 × 10⁻¹⁰). Tukey’s multiple comparisons test showed a highly significant difference between the PBS and PAPD + αPD-L1 groups (P = 6.18 × 10⁻¹¹).**

**
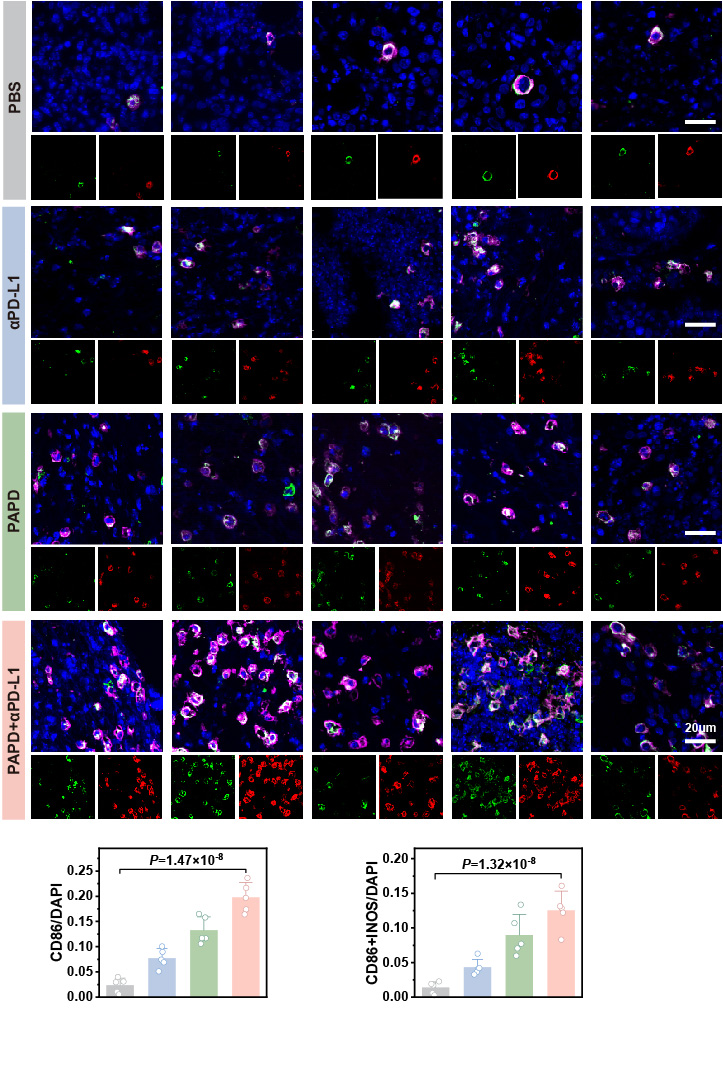
**

**Supplementary Fig. 42 | Immunofluorescence analysis of macrophage polarization in tumor tissues after different treatments. Representative immunofluorescence images of tumor sections from mice treated with PBS, αPD-L1, PAPD, and PAPD + αPD-L1 combination. CD86 (green), iNOS (red), and nuclei (blue, DAPI) staining are shown, with corresponding single-channel images presented below each merged image. Scale bar = 20 μm. The lower panels show quantitative analysis of CD86/DAPI and CD86+iNOS/DAPI-positive cell ratios across different treatment groups based on five randomly selected fields (mean ± s.d., n = 5 per group). One-way ANOVA revealed a significant overall difference among groups for both CD86 (F (3,16) = 51.17, P = 2.01 × 10⁻⁸) and CD86+iNOS (F (3,16) = 25.71, P = 2.01 × 10⁻⁶). Tukey’s multiple comparisons test showed significant differences among multiple groups, with the most pronounced increase observed in the PAPD + αPD-L1 group compared to the PBS group (CD86: P = 1.47 × 10⁻⁸; CD86+iNOS: P = 1.32 × 10⁻⁸).**

**Supplementary Tables**

**Supplementary Table 1. Experimental details of Pt_x_Te_y_:Ag_2_Te QDs.**

| **Samples** | **Inventory rating**  **Pt: Ag (mol)** | **TEM size (nm)** | **PL (nm)** |
| --- | --- | --- | --- |
| Ag_2_Te | 0 | 3.31 | 1550 |
| PtTe_2_:Ag_2_Te | 0.4 | 4.25 | 1650 |
| Pt_2_Te_3_:Ag_2_Te | 0.8 | 5.09 | 1730 |
| Pt_3_Te_4_:Ag_2_Te | 2.2 | 7.10 | 1970 |
| PtTe:Ag_2_Te | 3.0 | 8.06 | 2200 |

**Supplementary Table 2. Experimental details of Pt_x_Te_y_:Ag_2_Te QDs.**

| **Samples** | **Element** | **Atomic %** | **Pt:Ag (mol)** |
| --- | --- | --- | --- |
| Ag_2_Te | Ag | 74.1 | 0 |
|  | Te | 25.9 |  |
| PtTe_2_:Ag_2_Te | Ag | 64.1 | 4.8% |
|  | Te | 32.8 |  |
|  | Pt | 3.1 |  |
| Pt_2_Te_3_:Ag_2_Te | Ag | 62.8 | 6.2% |
|  | Te | 33.3 |  |
|  | Pt | 3.9 |  |
| Pt_3_Te_4_:Ag_2_Te | Ag | 59 | 11.7% |
|  | Te | 34.1 |  |
|  | Pt | 6.9 |  |
| PtTe:Ag_2_Te | Ag | 54.4 | 15.6% |
|  | Te | 37.1 |  |
|  | Pt | 8.5 |  |

Supplementary Table 3. The fitting of transient fluorescence spectra for Ag_2_Te and Pt_2_Te_3_:Ag_2_Te QDs

| **Samples** | **τ (ns)** | **τ_1_ (ns)** | **f_1_ (%)** | **τ_2_ (ns)** | **f_2_ (%)** | **χ^2^** |
| --- | --- | --- | --- | --- | --- | --- |
| Ag_2_Te | 4.82 | 4.13 | 91.66 | 12.40 | 8.34 | 1.16 |
| Pt_2_Te_3_:Ag_2_Te | 14.20 | 8.50 | 56.09 | 21.48 | 43.91 | 1.02 |

**Supplementary Table 4. Comparison of photothermal conversion efficiency (PCE) of representative theranostic nanomaterials**

| Materials | System type | PCE (%) | Reference |
| --- | --- | --- | --- |
| AuNRs | Gold nanorods | 36.8 | [12] |
| PtCu | Nanosheets | 20.4 | [13] |
| Ta_2_NiS_5_-P | 2D materials | 35.0 | [14] |
| O-CDs | Carbon dots | 58.0 | [15] |
| PTC-CDs | Carbon dots | 62.4 | [16] |
| CuInSe_2_ | Quantum dot hydrogels | 43.3 | [17] |
| AMQDs | Quantum dots | 45.5 | [18] |
| Ag_2_Te | Quantum dots | 50.5 | [19] |
| Pt_2_Te_3_:Ag_2_Te | Quantum dots | 67.9 | **This work** |

**Supplementary References**

[1] Z. Y. Liu, A. A. Liu, H. Fu, Q. Y. Cheng, M. Y. Zhang, M. M. Pan, L. P. Liu, M. Y. Luo, B. Tang, W. Zhao, J. Kong, X. Shao, D. W. Pang, *J. Am. Chem. Soc.* **2021**, *143*, 12867.

[2] R. C. Castro, M. L. M. F. S. Saraiva, J. L. M. Santos, D. S. M. Ribeiro, *Coord. Chem. Rev.* **2021**, *448*, 214181.

[3] Y. Cheng, H. Zhang, X. Qu, *Acc. Mater. Res*. **2021**, *2*, 764.

[4] J. C. Scimeca, E. Verron, *Mater. Today Adv.* **2022**, *15*, 100260.

[5] Z. Sun, C. Liu, H. Yang, X. Yang, Y. Zhang, H. Lin, Y. Li, Q. Wang, *Nano Res*. **2022**, *15*, 8555.

[6] C. Würth, M. Grabolle, J. Pauli, M. Spieles, U. Resch-genger, *Nat. Protoc*. **2013**, *8*, 1535.

[7] Z. Wang, Y. Liu, C. He, X. Zhang, X. Li, Y. Li, Y. Tang, X. Lu, Q. Fan, *Small*. **2024**, *20*, 2307829.

[8] H. Yang, H. Huang, X. Ma, Y. Zhang, X. Yang, M. Yu, Z. Sun, C. Li, F. Wu, Q. Wang, *Adv. Mater.* **2021**, *33*, 2103953.

[9] M. Yu, X. Yang, Y. Zhang, H. Yang, H. Huang, Z. Wang, J. Dong, R. Zhang, Z. Sun, C. Li, Q. Wang, *Small.* **2021**, *17*, 2006111.

[10] E. S. Welter, S. Garg, R. Gläser, M. Goepel, *ChemPhotoChem*. **2023**, *7*, e202300001.

[11] M. Wamsley, P. Wathudura, J. Hu, D. Zhang, *Anal. Chem*. **2022**, *94*, 11610.

[12] N. Alifu, X. Zhang, *ACS Appl. Nano Mater*. **2021**, *4*, 13060−13070

[13] Y. Zhang, C. Shen, J. Zhang, Q. Shen, F. Xu, S. Wang, J. Hu, F. Saleem, F. Huang, Z. Luo, *Chinese Chem. Lett.* **2025**, *36*, 111059.

[14] H. Zhu, Z. Lai, Y. Fang, X. Zhen, C. Tan, X. Qi, D. Ding, P. Chen, H. Zhang, K. Pu, *Small*. **2017**, *13*, 1604139.

[15] S. Deb, R. Acharya, K. Lim, J. Kim, *Colloids Surfaces A Physicochem. Eng. Asp.* **2024**, *696*, 134266.

[16] G. Liu, B. Li, J. Li, J. Dong, V. E. Baulin, Y. Feng, D. Jia, Y. V Petrov, A. Y. Tsivadze, Y. Zhou, *ACS Appl. Mater. Interfaces*. **2023**, *15*, 55335.

[17] H. Gao, Y. Liu, W. Lian, P. Hu, X. Shang, M. Chen, *Nano Today.* **2024**, *58*, 102436.

[18] W. Tao, X. Ji, X. Xu, M. A. Islam, Z. Li, S. Chen, P. E. Saw, H. Zhang, Z. Bharwani, Z. Guo, J. Shi, O. C. Farokhzad, *Angew. Chem. Int. Ed*. **2017**, *129*, 12058.

[19] L. Dong, W. Li, L. Yu, L. Sun, Y. Chen, G. Hong, *ACS Appl. Mater. Interfaces*. **2020**, *12*, 42558.
